# Predicting protein function by machine learning on amino acid sequences – a critical evaluation

**DOI:** 10.1186/1471-2164-8-78

**Published:** 2007-03-20

**Authors:** Ali Al-Shahib, Rainer Breitling, David R Gilbert

**Affiliations:** 1 Biomedical Informatics Signals and Systems Research Laboratory, Department of Electronic, Electrical and Computer Engineering, The University of Birmingham, Birmingham, UK; 2 Bioinformatics Research Centre, Department of Computing Science, University of Glasgow, Glasgow, UK; 3 Groningen Bioinformatics Centre, Groningen Biomolecular Sciences and Biotechnology Institute, University of Groningen, 9751 NN Haren, The Netherlands

## Abstract

**Background:**

Predicting the function of newly discovered proteins by simply inspecting their amino acid sequence is one of the major challenges of post-genomic computational biology, especially when done without recourse to experimentation or homology information. Machine learning classifiers are able to discriminate between proteins belonging to different functional classes. Until now, however, it has been unclear if this ability would be transferable to proteins of unknown function, which may show distinct biases compared to experimentally more tractable proteins.

**Results:**

Here we show that proteins with known and unknown function do indeed differ significantly. We then show that proteins from different bacterial species also differ to an even larger and very surprising extent, but that functional classifiers nonetheless generalize successfully across species boundaries. We also show that in the case of highly specialized proteomes classifiers from a different, but more conventional, species may in fact outperform the endogenous species-specific classifier.

**Conclusion:**

We conclude that there is very good prospect of successfully predicting the function of yet uncharacterized proteins using machine learning classifiers trained on proteins of known function.

## Background

Genome sequencing projects continue to produce unprecedented amounts of novel protein sequence information, and large-scale experimental efforts are underway to determine the function of the newly discovered proteins [1-6]. For a majority of proteins it is already possible to predict their approximate function with reasonable accuracy based on their evolutionary relationship or sequence similarity to proteins with known functions [7-9]. For most recently sequenced bacterial genomes about three quarters of open reading frames can be assigned a possible function in this way. However, a significant number of predicted proteins in each newly sequenced genome have turned out to defy this approach. These proteins, which in extreme cases may constitute up to 50% of open reading frames, show no similarity to proteins of known function. This may be due to missing experimental data, or the proteins are evolving too rapidly or are even unique to a small clade of species.

It would be very useful if one could obtain at least a general idea of the function of such proteins based on their amino acid sequence alone. Of course this is an extremely challenging task, and one that will only be of limited usefulness without combining it with additional information (e.g. structure models, phylogenetic profiles, or genomic context), but nonetheless several techniques to address this issue have been proposed recently [10-16]. These publications show that using machine learning classifiers it is possible to predict the function of well-characterized proteins based on features of their amino acid sequence, without using homology information [17]. However, it is unclear if and how well such classifiers would transfer to proteins of unknown function. There are many reasons to assume that these 'unknown' proteins are special and differ from well-characterized proteins in significant ways: They may be evolving at a faster pace, they may function in unconventional ways, they may have unusual physico-chemical properties that have made them less accessible to experimentation. If 'unknown' proteins are not just a random subset of the proteome, but are biased in such a systematic fashion, classifiers trained and tested on proteins of known function may generalize poorly and will be unable to predict the function of the real proteins of interest.

A direct test of the predictive performance on proteins of unknown function is rarely possible, although a recent retrospective study [18] made some first steps in that direction. Thus a critical systematic assessment of the general prospect of successful classifier transfer is of great interest.

Here we show that proteins of known and unknown function do indeed differ significantly. We go on to show that, surprisingly, proteins from different species do also differ, to an even larger extent. We then demonstrate that classifiers do nonetheless generalize across species boundaries and use this to provide the first critical estimate of predictive performance on proteins of unknown function.

## Results and discussion

We based our analysis on the completed and annotated proteomes of seven bacterial pathogens which cause sexually transmitted diseases in humans (Table 1). These species cover a wide range of phylogenetic relationships, from closely related species (two mycoplasma species) to very divergent ones (*Treponema, Chlamydia*). On the other hand, they all share the same general ecological niche, thus minimizing confounding effects of divergent evolutionary adaptation.

### Prediction of known protein functions

In this paper we are not interested in optimizing a method of predicting protein functions, but rather in evaluating an aspect of function prediction that has been somewhat neglected previously, namely whether classifiers trained on proteins of known functions can be expected to transfer successfully to proteins of unknown function. Even an optimal classifier would be useless if it could not be applied reliably to the real proteins of interest, i.e. those for which no function is known at present.

**Figure 1:**
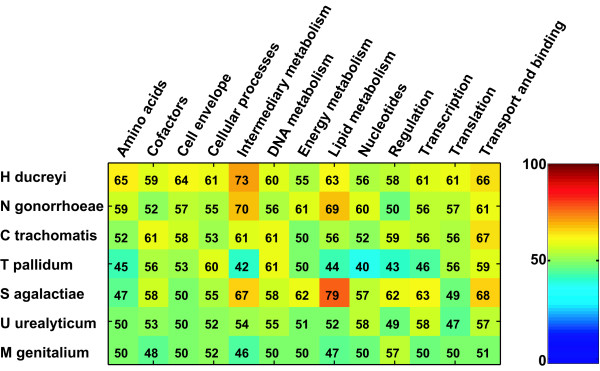
**Function prediction classifier performance**. Performance for seven human pathogens and thirteen functional classes is shown as % AUC, and values larger than 50 indicate a better than random classifier. One can see that in four of the seven species prediction results are significantly better than random across all classes. Only on three small genomes (*T. pallidum, U. urealyticum *and *M. genitalium*) performance is much weaker. Specific classes (co-factor metabolism, cellular processes, DNA metabolism) show particularly good performance. The functional class that is most easily distinguished from the others contains 'transport and binding proteins' – this good performance is probably due to the characteristic hydrophobic motifs in the transmembrane and binding regions of these proteins. Colors indicate the AUC values, ranging from 0 (dark blue) to 100% (dark red). The same color scale is used for all figures in this paper.

However, as a baseline for our study, we first showed that we are able to correctly predict the function of proteins with known function using a Support Vector Machine classifier based on features derived from their amino acid sequence alone (see Methods for details of feature definition and selection and the machine learning technique). Confirming previous results [10,12,16] we found that this is indeed possible, although with varying performance for each class and species (Figure 1). Only in three highly specialized bacterial species (*Treponema *and the two mycoplasmas) overall performance was hardly better than random, and we will show below how the results of the present work indicate a way to overcome this problem. The observed median AUC is 56% averaged across all species and functional classes, and is higher for some important functional classes such as intermediary metabolism, DNA metabolism, and transport and binding proteins. These results are equivalent to previously reported accuracies [10,12,16]. The generally good performance on such small bacterial genomes is encouraging, especially as it does not rely on the use of posttranslational modification and localization predictions, which are very informative features for eukaryotic proteins [13-15].

### Discrimination between 'known' and 'unknown' proteins

**Figure 2:**
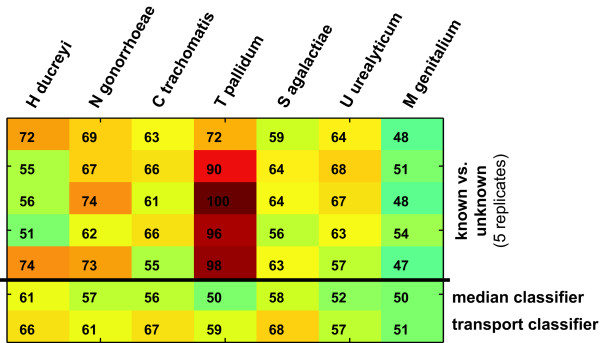
**Discrimination between proteins of known and unknown function**. The results of five random splits of test and training set are shown, and for comparison the lower two rows show the median performance of the function prediction classifier and the 'transport and binding' classifier for each species. For each species, except *M. genitalium *the average performance on the known-vs.-unknown task is better than on the function prediction task. In the case of *T. pallidum*, known and unknown proteins can be distinguished with almost perfect performance.

Previous studies in general stopped at this point and assumed that predictive performance would be maintained when the classifier were applied to proteins of unknown function. We wanted to know if that is a reasonable assumption. To determine the overall similarity of known and unknown proteins in the feature space used for function prediction, we trained another set of SVM classifiers to try to distinguish between these two sets of proteins. Not unexpectedly we found that they do indeed differ significantly (Figure 2). The possible reason why this is the case lies in the type of unknown proteins. A set of unknown proteins in a species typically contains:

• **Unique proteins **– Proteins without known homologs

• **Hypothetical proteins **– Proteins with homologs of unknown function. No experimental evidence exists for the function or existence of the protein product.

• **Wrongly predicted proteins **– Open reading frames that are not actually expressed (transcribed/translated), but are only the result of genome misannotation.

• **Special proteins **– Proteins that have a special feature (e.g. an unusual size or extreme amount of charged amino acids) making them different from known proteins, but which do have a biological function.

From the above list, one can see that the set of unknown proteins will contain some members that actually will have a function and others that are probably genome annotation artifacts. The ability to distinguish between known and unknown proteins is most likely due to the difference between unusual unknown proteins (categories 3 and 4) and normal known proteins. It is expected that homology-less function prediction will be possible for the 'normal' and unknown proteins (categories 1 and 2), while being much more difficult for the special proteins (category 4) and meaningless for wrongly predicted proteins (category 3). Therefore, it would be interesting to use the clasification information to estimate the fraction of 'predictable' and 'unpredictable' proteins in the set of unknown proteins. An exact estimate is not possible, because there is no exact definition of a normal protein available, but we can use the performance of the SVM classifier to obtain a rough estimate. The median AUC for the discrimination between proteins of known and unknown function is 63%. If we assume that 'predictable' unknown proteins are indistinguishable from known proteins, we can calculate the lower bound estimate of the fraction of unpredictable proteins to be 26% (=(63%–50%)/50%).

### Discrimination between proteins from different bacteria

**Figure 3:**
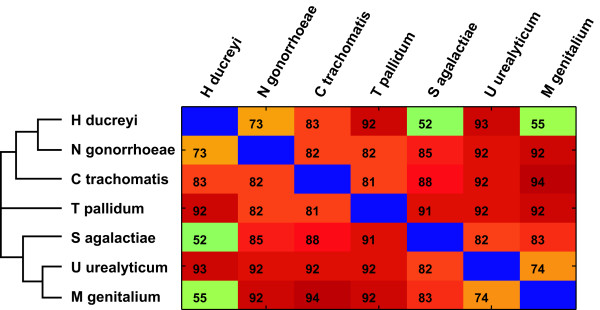
**Species-species discrimination**. The AUC for classifiers trained to distinguish between proteins from each species pair is shown (median of five replicates). With the exception of *H. ducreyi *vs. *S. agalactiae *and *H. ducreyi *vs. *M. genitalium*, all comparisons yield excellent classification performance. This means that proteins from different source organisms can be distinguished with surprising accuracy based solely on amino acid sequence features. The unrooted tree to the left shows the phylogenetic relationships of the seven bacterial species, based on 16S rRNA analysis.

To determine if the effect of protein set dissimilarity will be as deleterious as one might fear, we argued that proteins from different species will also show some level of dissimilarity and, hence, one could use the performance of classifers across species boundaries to estimate the transferability from known to unknown proteins. Using our taxonomically diverse sample of bacterial species we trained a new set of SVM classifiers to try to distinguish between each pair of species. To our great surprise we found that this task is far easier than function prediction or the discrimination of known and unknown proteins (Figure 3). The median AUC across all species pairs is an astonishing 85%. This means that given any randomly picked pair of proteins from species A and B, we will be able to assign them to their correct species of origin in 85% of cases. This finding is entirely unexpected, given that the bacteria in our dataset all share the same highly stable ecological niche, the human urogenital tract. While they naturally differ widely in their exact pathophysiology, they would still be expected to carry out the same general biological processes using very similar molecular machinery. The fact that the SVM classifiers are nonetheless able to find generally valid species-specific "sequence signatures" is of course of great biological interest.

One possible explanation for this high accuracy in discriminating proteins from different species lies in the varying levels of guanine-cytosin (GC) content in each species(Table 1). Variations in genomic GC content from 25% to 75% have been shown to be common in prokaryotes [19]. Variations in GC content in coding sequences will be reflected in differences of amino acid composition, as GC rich codons will be depleted in low-GC species and vice versa. Even if this variation is subtle, it will influence classifier performance.

### Classifier transfer between species

**Figure 4:**
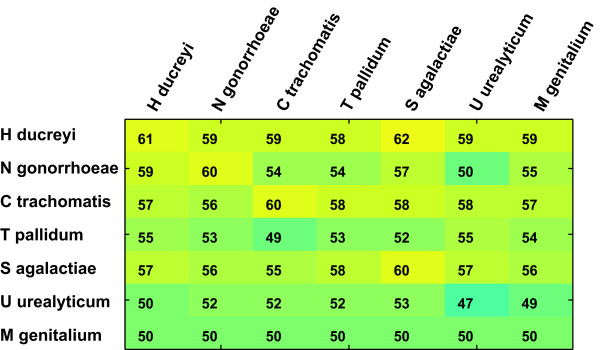
**Classifier transfer across species boundaries**. The median AUC for 13 functional classes is shown. The 'training species' is shown in the rows, the 'test' species in the columns. It can be seen that classifiers perform almost as well on a 'foreign' species as they do on the species they were originally trained on (diagonal). Performance is worst for the classifiers from *T. pallidum, U. urealyticum *and *M. genitalium*, and in these three cases the classifiers from the other four species give significantly better performance than those from the original species (sign test, *p *< 0.001).

**Figure 5:**
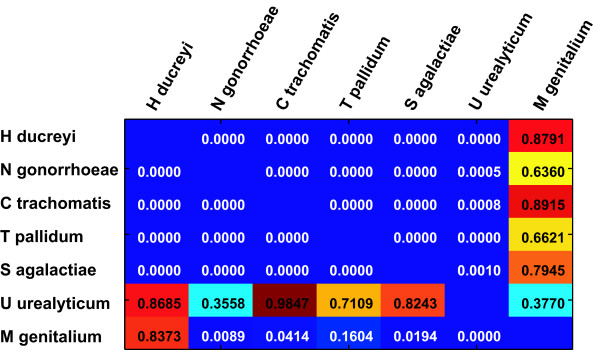
**Feature concordance between species**. The feature lists selected for function prediction in each species using the Wilcoxon filter as described were analyzed for concordance. The feature selection procedure generates sorted lists of features. The agreement between these lists can be calculated using a rank correlation method, for example Kendall's Coefficient of Concordance. A good correlation (reflected in a small *p*-value) indicates that the same features are high in the list of selected features. The *p*-values of Kendall's Coefficient of Concordance for each pairwise comparison are shown. The feature lists for the first five species show high correlation, while those of the two mycoplasmal species differ significantly. This may explain the difference in performance on these two species. Note that the matrix is not symmetrical, because different features will be removed by the redundancy filtering step depending on which species is used as a reference

How then does this high level of dissimilarity between species affect the performance of function prediction classifiers? Figure 4 shows that classifiers transfer across species boundaries with surprisingly little loss of accuracy. Classifiers that perform well on their species of origin do almost as well on each of the other species. High levels of protein set dissimilarity are apparently tolerated without decreasing performance. A case of special interest is represented by the mycoplasma genomes, where classifiers perform poorly if they are trained on the species itself, and functions are predicted with higher accuracy if the classifier comes from one of the non-mycoplasma species. Mycoplasma species are highly derived organisms with extremely reduced minimal genomes and their proteomes may be specifically adapted, e.g. the features used for their SVM classifiers differ entirely from those of the other species (Figure 5), but the paradoxical cross-species performance is still difficult to explain by this fact alone.

## Conclusion

**Figure 6:**
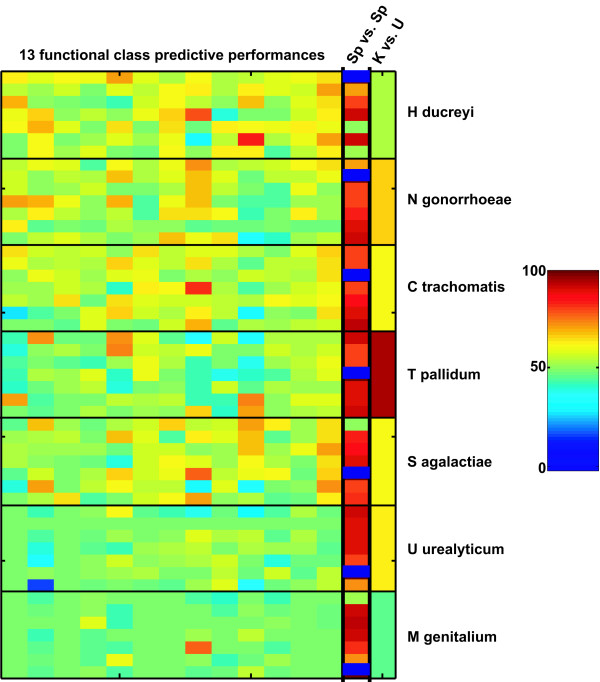
**Summary of predictive performance and expected performance on proteins of unknown function**. The first 13 columns show the AUCs for each functional class in each of the 7 × 7 species-species transfers. The order of functional classes and species is the same as in figure 1. The 14th column shows the corresponding species-species discrimination AUCs (from Figure 3) and the 15th column the distinction between known and unknown proteins for the species from which the classifier is derived (from Figure 2). To predict the expected performance on proteins of unknown function, find the species-species contrast that corresponds most closely to the known-unknown contrast of interest. The corresponding function prediction AUCs should give a reasonable estimate for the expected performance. It can be seen here that functional classes that are easily distinguished within a species will also successfully transfer between species, and such predictors (e.g. 'transport and binding', column 13) will also yield reliable results on the proteins of unknown function.

In conclusion, we find that proteins with known and unknown function differ significantly, but we also find that classifiers transfer very well between different bacterial species which differ even more. Viewed optimistically, this means that there is a distinct possibility that function prediction classifiers will generalize successfully to predict the function of proteins of unknown function. Figure 6 summarizes the results and can also be used to estimate the performance of classifiers for unknown proteins. In most cases, this performance will be almost as good as that on the known proteins. Our findings also indicate that, especially in the case of "unusual" proteomes, such as the mycoplasmal examples, it may be a promising strategy to train classifiers on related but more conventional species to achieve the highest predictive performance.

## Methods

### Protein dataset and annotation

Protein sequences for seven bacterial pathogens causing sexually transmitted diseases in humans (Table 1) were obtained from the Los Alamos National Laboratory Bioscience Division STD Sequence Databases [20]. For each functionally characterized protein its classfication in one of 13 functional classes based on a modified version of the Riley scheme [21] was obtained from the same source.

### Definition of protein sequence features

For every protein we calculated the frequency and total number of each amino acid, as well as of certain sets of amino acids (e.g. hydrophobic, charged, polar). To encode distributional features we also determined the number and size of continuous stretches of each amino acid or amino acid set. We also subdivided every protein into four equally sized fragments and calculated the same feature values for each fragment and combination of fragments. In addition, we predicted the secondary structure using Prof [22], the position of putative transmembrane helices using TMHMM [23] and of disordered regions using DisEMBL [24], and treated the obtained predictions in the same way as the amino acids. A small number of global features (e.g. isoelectric point and molecular weight) were also included. The total number of features extracted for every protein is 2579. The full feature set is described in Additional File 1.

### Standardization of feature values

Since the original features generated in this way are very heterogeneously scaled linear normalization (standardization) was performed to rescale each feature by its mean and variance. After standardization, each of the 2579 features has a mean of 0 and a standard deviation of 1.

### Homology-corrected generation of test and training sets

The entire dataset was subdivided randomly five times into test and training sets (size ratio 1:4). To prevent inflation of the prediction accuracies by predictions on homologous sequences in the test set, we applied a recursive Blast strategy to assign proteins that show significant sequence similarity to each other to the same set (either test or training). For this purpose every protein that was added to the test set was searched in three PSI-Blast iterations [25] against the non-redundant database of protein sequences at NCBI [26] using default settings. The obtained position-specific sequence profile was then run against the bacterial proteins and every protein generating a hit at *E *< 0.001 was also added to the test set, and the procedure repeated recursively until no new potential homologues were detected. Then the next randomly chosen protein would be added to the test set until the required test set size was exceeded.

### Feature selection

For every training set, species and task, we selected discriminatory features using a simple filter approach which in previous work performed as well as classical wrapper approaches (data not shown). Briefly, for every feature we performed a Wilcoxon signed-rank test for every comparison of functional classes. Features were retained if for at least one comparison of classes they had a Wilcoxon *p*-value less than 0.02, indicating that they contribute potentially discriminating information. A second step of filtering removed highly redundant features, so that the remaining features had a pairwise absolute correlation coefficient of less than 0.95. For the known-unknown and species-species discrimination tasks the same procedure was applied using the Wilcoxon results for feature values in the various species or in 'known' vs. 'unknown' proteins, respectively.

### Classifier generation

Classification was done using Support Vector Machine classifiers as implemented in the WEKA machine learning package [27]. As the datasets are highly imbalanced the negative class was undersampled to equal the positive class [28]. A simple polynomial kernel with order 3 was used, as it had shown good performance in previous related studies [28]. Other parameters were used in default settings (complexity constant = 1, size of the kernel cache = 1 × 10^4^, tolerance parameter = 1.03 × 10^-03^) to avoid introducing bias by fine tuning to the present data. For the functional class prediction, one-against-all classifiers were generated for each class. For example, for predicting the transport and binding proteins functional class, we labeled all the other 12 functional classes as 'not transport and binding proteins' and performed a binary classification of transport and binding proteins against 'not transport and binding proteins'. We could then assess how well the features discriminate between the transport and binding functional class and all other functional classes.

### Classifier performance evaluation

Classifier performances were evaluated using the Area Under the Receiver Operating Characteristic curve (AUC) on the test set. The median over the five splits of the test and training sets is generally reported. This value is a non-parametric estimate of the discriminating ability of the classifier. A value of 50% corresponds to a random classifier, a value of 100% indicates perfect performance [29,30]. Using the AUC as a descriptor of classifier performance has the important advantage that it is independent of the class distribution in the test set. This is very important for our protein function prediction task: It is highly unlikely that the distribution of functions among the 'unknown' proteins is the same as that of the 'known' proteins, and the AUC provides the most unbiased performance estimate in this situation.

## Authors' contributions

AA collected the protein sequence data, generated the feature database and performed all experiments. RB designed the study, implemented the feature selection algorithm, and drafted the manuscript. DRG supervised the project and provided critical input. All authors read and approved the final manuscript.

## Supplementary Material

Additional file 1 **Full list of protein sequence features**. PDF file defining the 2579 protein sequence features used for as input for the feature selection and classification process. Click here for file

## Figures and Tables

**Table 1:** Bacterial species used in the analysis.

Species	Total # of proteins	# of 'unknowns'	% 'unknowns'	% Average GC content
*Haemophilus ducreyi*	1830	381	21	38.22
*Neisseria gonorrhoeae*	2188	667	30	52.69
*Chlamydia trachomatis*	902	318	35	41.31
*Treponema pallidum*	1051	28	5	52.77
*Streptococcus agalactiae*	2177	567	26	35.65
*Ureaplasma urealyticum*	614	275	48	25.50
*Mycoplasma genitalium*	485	158	33	31.69
