# Supplementary material for: Predicting protein function by machine learning on amino acid sequences – a critical evaluation
Source: BMC Genomics. 2007 Mar 20;8:78. doi: 10.1186/1471-2164-8-78 (PMC1847686; doi:10.1186/1471-2164-8-78)
Supplement: Additional file 1 — Full list of protein sequence features. PDF file defining the 2579 protein sequence features used for as input for the feature selection and classification process. [file 1471-2164-8-78-S1.pdf]

Amino Acid Sequence Features Used for Predicting Protein Function by Machine Learning

April 28, 2005

**1**	**2**	**3**	**4**	**5**	**6**	**7**	**8**	**9**	**10**	**11**	**12**	**13**
Ala	-	X	X	-	-	X	-	-	-	-	-	-
Cys	-	X	X	-	-	-	-	-	-	-	-	-
Asp	X	-	-	-	-	-	-	X	-	X	X	-
Glu	X	-	-	-	-	-	-	X	-	X	X	-
Phe	-	X	X	-	X	-	-	-	-	-	-	-
Gly	-	X	X	-	-	X	-	-	-	-	-	-
His	-	X	-	-	X	-	-	X	X	-	X	-
Lys	X	-	X	-	-	-	-	X	X	-	X	-
Ile	-	X	X	X	-	-	-	-	-	-	-	-
Leu	-	X	X	X	-	-	-	-	-	-	-	-
Met	-	X	X	-	-	-	X	-	-	-	-	-
Asn	X	-	-	-	-	-	X	-	-	-	X	X
Pro	X	-	-	-	-	-	X	-	-	-	-	-
Gln	X	-	-	-	-	-	X	-	-	-	X	X
Arg	X	-	-	-	-	-	-	X	X	-	X	-
Ser	X	-	X	-	-	X	-	-	-	-	X	-
Thr	-	X	-	-	-	-	X	-	-	-	X	-
Val	-	X	X	X	-	-	-	-	-	-	-	-
Trp	-	X	X	-	X	-	-	-	-	-	X	-
Tyr	-	X	-	-	X	-	-	-	-	-	X	-

Table 1: Definition of amino acid sets used in the description of sequence features. 1 = Amino Acids, 2 = Hydrophilic, 3 = Hydrophobic, 4 = Large Hydrophobic, 5 = Aliphatic, 6 = Aromatic, 7 = Tiny, 8 = Diverse, 9 = Charged, 10 = Positive, 11 = Negative, 12 = Polar, 13 = Amides.

1. The protein length
2. The molecular weight of the protein
3. The isoelectric point of the protein
4. The aliphatic index of the protein
5. The Grand Average of Hydropathicity (GRAVY) of the protein

6. The total number of hydrophilic residues in the sequence
7. The total number of hydrophilic residues divided by the length of the protein
8. The total number of hydrophilic residues in the first quarter of the sequence
9. The total number of hydrophilic residues in the second quarter of the sequence
10. The total number of hydrophilic residues in the third quarter of the sequence
11. The total number of hydrophilic residues in the fourth quarter of the sequence
12. The total number of hydrophilic residues in the first quarter of the sequence divided by the length of the protein
13. The total number of hydrophilic residues in the second quarter of the sequence divided by the length of the protein
14. The total number of hydrophilic residues in the third quarter of the sequence divided by the length of the protein
15. The total number of hydrophilic residues in the fourth quarter of the sequence divided by the length of the protein
16. The total number of hydrophilic residues in the first quarter of the sequence divided by the total number of hydrophilic blocks
17. The total number of hydrophilic residues in the first quarter of the sequence divided by the total number of hydrophilic blocks
18. The total number of hydrophilic residues in the first quarter of the sequence divided by the total number of hydrophilic blocks
19. The total number of hydrophilic residues in the first quarter of the sequence divided by the total number of hydrophilic blocks
20. The total number of hydrophilic residues in the first half of the protein
21. The total number of hydrophilic residues in the three-quarter region of the protein
22. The total number of hydrophilic residues from position 25% to 75% of the protein
23. The total number of hydrophilic residues in the second half of the protein
24. The total number of hydrophilic residues in the first half of the protein divided by the protein length
25. The total number of hydrophilic residues in the three-quarter region of the protein divided by the protein length

26. The total number of hydrophilic residues from position 25% to 75% of the protein divided by the protein length
27. The total number of hydrophilic residues in the second half of the protein divided by the protein length
28. The total number of hydrophilic residues in the first half of the protein divided by the total number of hydrophilic residues in the protein
29. The total number of hydrophilic residues in the three-quarter region of the protein divided by the total number of hydrophilic residues in the protein
30. The total number of hydrophilic residues from position 25% to 75% of the protein divided by the total number of hydrophilic residues in the protein
31. The total number of hydrophilic residues in the second half of the protein divided by the total number of hydrophilic residues in the protein
32. The total number of hydrophilic residue blocks in the protein
33. The total number of hydrophilic residue blocks in the first quarter of the protein
34. The total number of hydrophilic residue blocks in the second quarter of the protein
35. The total number of hydrophilic residue blocks in the third quarter of the protein
36. The total number of hydrophilic residue blocks in the fourth quarter of the protein
37. The total number of hydrophilic residue blocks in the first quarter of the protein divided by the protein length
38. The total number of hydrophilic residue blocks in the second quarter of the protein divided by the protein length
39. The total number of hydrophilic residue blocks in the third quarter of the protein divided by the protein length
40. The total number of hydrophilic residue blocks in the fourth quarter of the protein divided by the protein length
41. The total number of hydrophilic residue blocks in the first quarter of the protein divided by the total number of hydrophilic blocks of the protein
42. The total number of hydrophilic residue blocks in the second quarter of the protein divided by the total number of hydrophilic blocks of the protein
43. The total number of hydrophilic residue blocks in the third quarter of the protein divided by the total number of hydrophilic blocks of the protein
44. The total number of hydrophilic residue blocks in the fourth quarter of the protein divided by the total number of hydrophilic blocks of the protein

45. The total number of hydrophilic residue blocks in the first half of the protein
46. The total number of hydrophilic residue blocks in the three-quarter region of the protein
47. The total number of hydrophilic residue blocks from position 25% to 75% of the protein
48. The total number of hydrophilic residue blocks in the second half of the protein
49. The total number of hydrophilic residue blocks in the first half of the protein divided by the protein length
50. The total number of hydrophilic residue blocks in the three-quarter region of the protein divided by the protein length
51. The total number of hydrophilic residue blocks from position 25% to 75% of the protein divided by the protein length
52. The total number of hydrophilic residue blocks in the second half of the protein divided by the protein length
53. The total number of hydrophilic residue blocks in the first half of the protein divided by the total number of hydrophilic blocks of the protein
54. The total number of hydrophilic residue blocks in the three-quarter region of the protein divided by the total number of hydrophilic blocks of the protein
55. The total number of hydrophilic residue blocks from position 25% to 75% of the protein divided by the total number of hydrophilic blocks of the protein
56. The total number of hydrophilic residue blocks in the second half of the protein divided by the total number of hydrophilic blocks of the protein
57. The length of the maximum hydrophilic block in the first quarter of the protein
58. The median of the hydrophilic blocks in the first quarter of the protein
59. The mean of the hydrophilic blocks in the first quarter of the protein
60. The length of the maximum hydrophilic block in the second quarter of the protein
61. The median of the hydrophilic blocks in the second quarter of the protein
62. The mean of the hydrophilic blocks in the second quarter of the protein
63. The length of the maximum hydrophilic block in the third quarter of the protein
64. The median of the hydrophilic blocks in the third quarter of the protein

65. The mean of the hydrophilic blocks in the third quarter of the protein
66. The length of the maximum hydrophilic block in the fourth quarter of the protein
67. The median of the hydrophilic blocks in the fourth quarter of the protein
68. The mean of the hydrophilic blocks in the fourth quarter of the protein
69. The length of the maximum hydrophilic block of the protein
70. The median of the hydrophilic blocks of the protein
71. The mean of the hydrophilic blocks of the protein
72. The total number of hydrophobic residues in the sequence
73. The total number of hydrophobic residues divided by the length of the protein
74. The total number of hydrophobic residues in the first quarter of the sequence
75. The total number of hydrophobic residues in the second quarter of the sequence
76. The total number of hydrophobic residues in the third quarter of the sequence
77. The total number of hydrophobic residues in the fourth quarter of the sequence
78. The total number of hydrophobic residues in the first quarter of the sequence divided by the length of the protein
79. The total number of hydrophobic residues in the second quarter of the sequence divided by the length of the protein
80. The total number of hydrophobic residues in the third quarter of the sequence divided by the length of the protein
81. The total number of hydrophobic residues in the fourth quarter of the sequence divided by the length of the protein
82. The total number of hydrophobic residues in the first quarter of the sequence divided by the total number of hydrophobic blocks
83. The total number of hydrophobic residues in the first quarter of the sequence divided by the total number of hydrophobic blocks
84. The total number of hydrophobic residues in the first quarter of the sequence divided by the total number of hydrophobic blocks
85. The total number of hydrophobic residues in the first quarter of the sequence divided by the total number of hydrophobic blocks
86. The total number of hydrophobic residues in the first half of the protein

87. The total number of hydrophobic residues in the three-quarter region of the protein
88. The total number of hydrophobic residues from position 25% to 75% of the protein
89. The total number of hydrophobic residues in the second half of the protein
90. The total number of hydrophobic residues in the first half of the protein divided by the protein length
91. The total number of hydrophobic residues in the three-quarter region of the protein divided by the protein length
92. The total number of hydrophobic residues from position 25% to 75% of the protein divided by the protein length
93. The total number of hydrophobic residues in the second half of the protein divided by the protein length
94. The total number of hydrophobic residues in the first half of the protein divided by the total number of hydrophobic residues in the protein
95. The total number of hydrophobic residues in the three-quarter region of the protein divided by the total number of hydrophobic residues in the protein
96. The total number of hydrophobic residues from position 25% to 75% of the protein divided by the total number of hydrophobic residues in the protein
97. The total number of hydrophobic residues in the second half of the protein divided by the total number of hydrophobic residues in the protein
98. The total number of hydrophobic residue blocks in the protein
99. The total number of hydrophobic residue blocks in the first quarter of the protein
100. The total number of hydrophobic residue blocks in the second quarter of the protein
101. The total number of hydrophobic residue blocks in the third quarter of the protein
102. The total number of hydrophobic residue blocks in the fourth quarter of the protein
103. The total number of hydrophobic residue blocks in the first quarter of the protein divided by the protein length
104. The total number of hydrophobic residue blocks in the second quarter of the protein divided by the protein length
105. The total number of hydrophobic residue blocks in the third quarter of the protein divided by the protein length

106. The total number of hydrophobic residue blocks in the fourth quarter of the protein divided by the protein length
107. The total number of hydrophobic residue blocks in the first quarter of the protein divided by the total number of hydrophobic blocks of the protein
108. The total number of hydrophobic residue blocks in the second quarter of the protein divided by the total number of hydrophobic blocks of the protein
109. The total number of hydrophobic residue blocks in the third quarter of the protein divided by the total number of hydrophobic blocks of the protein
110. The total number of hydrophobic residue blocks in the fourth quarter of the protein divided by the total number of hydrophobic blocks of the protein
111. The total number of hydrophobic residue blocks in the first half of the protein
112. The total number of hydrophobic residue blocks in the three-quarter region of the protein
113. The total number of hydrophobic residue blocks from position 25% to 75% of the protein
114. The total number of hydrophobic residue blocks in the second half of the protein
115. The total number of hydrophobic residue blocks in the first half of the protein divided by the protein length
116. The total number of hydrophobic residue blocks in the three-quarter region of the protein divided by the protein length
117. The total number of hydrophobic residue blocks from position 25% to 75% of the protein divided by the protein length
118. The total number of hydrophobic residue blocks in the second half of the protein divided by the protein length
119. The total number of hydrophobic residue blocks in the first half of the protein divided by the total number of hydrophobic blocks of the protein
120. The total number of hydrophobic residue blocks in the three-quarter region of the protein divided by the total number of hydrophobic blocks of the protein
121. The total number of hydrophobic residue blocks from position 25% to 75% of the protein divided by the total number of hydrophobic blocks of the protein
122. The total number of hydrophobic residue blocks in the second half of the protein divided by the total number of hydrophobic blocks of the protein

123. The length of the maximum hydrophobic block in the first quarter of the protein
124. The median of the hydrophobic blocks in the first quarter of the protein
125. The mean of the hydrophobic blocks in the first quarter of the protein
126. The length of the maximum hydrophobic block in the second quarter of the protein
127. The median of the hydrophobic blocks in the second quarter of the protein
128. The mean of the hydrophobic blocks in the second quarter of the protein
129. The length of the maximum hydrophobic block in the third quarter of the protein
130. The median of the hydrophobic blocks in the third quarter of the protein
131. The mean of the hydrophobic blocks in the third quarter of the protein
132. The length of the maximum hydrophobic block in the fourth quarter of the protein
133. The median of the hydrophobic blocks in the fourth quarter of the protein
134. The mean of the hydrophobic blocks in the fourth quarter of the protein
135. The length of the maximum hydrophobic block of the protein
136. The median of the hydrophobic blocks of the protein
137. The mean of the hydrophobic blocks of the protein
138. The total number of large hydrophobic residues in the sequence
139. The total number of large hydrophobic residues divided by the length of the protein
140. The total number of large hydrophobic residues in the first quarter of the sequence
141. The total number of large hydrophobic residues in the second quarter of the sequence
142. The total number of large hydrophobic residues in the third quarter of the sequence
143. The total number of large hydrophobic residues in the fourth quarter of the sequence
144. The total number of large hydrophobic residues in the first quarter of the sequence divided by the length of the protein
145. The total number of large hydrophobic residues in the second quarter of the sequence divided by the length of the protein

146. The total number of large hydrophobic residues in the third quarter of the sequence divided by the length of the protein

147. The total number of large hydrophobic residues in the fourth quarter of the sequence divided by the length of the protein

148. The total number of large hydrophobic residues in the first quarter of the sequence divided by the total number of large hydrophobic blocks

149. The total number of large hydrophobic residues in the first quarter of the sequence divided by the total number of large hydrophobic blocks

150. The total number of large hydrophobic residues in the first quarter of the sequence divided by the total number of large hydrophobic blocks

151. The total number of large hydrophobic residues in the first quarter of the sequence divided by the total number of large hydrophobic blocks

152. The total number of large hydrophobic residues in the first half of the protein

153. The total number of large hydrophobic residues in the three-quarter region of the protein

154. The total number of large hydrophobic residues from position 25% to 75% of the protein

155. The total number of large hydrophobic residues in the second half of the protein

156. The total number of large hydrophobic residues in the first half of the protein divided by the protein length

157. The total number of large hydrophobic residues in the three-quarter region of the protein divided by the protein length

158. The total number of large hydrophobic residues from position 25% to 75% of the protein divided by the protein length

159. The total number of large hydrophobic residues in the second half of the protein divided by the protein length

160. The total number of large hydrophobic residues in the first half of the protein divided by the total number of large hydrophobic residues in the protein

161. The total number of large hydrophobic residues in the three-quarter region of the protein divided by the total number of large hydrophobic residues in the protein

162. The total number of large hydrophobic residues from position 25% to 75% of the protein divided by the total number of large hydrophobic residues in the protein

163. The total number of large hydrophobic residues in the second half of the protein divided by the total number of large hydrophobic residues in the protein

164. The total number of large hydrophobic residue blocks in the protein

165. The total number of large hydrophobic residue blocks in the first quarter of the protein

166. The total number of large hydrophobic residue blocks in the second quarter of the protein

167. The total number of large hydrophobic residue blocks in the third quarter of the protein

168. The total number of large hydrophobic residue blocks in the fourth quarter of the protein

169. The total number of large hydrophobic residue blocks in the first quarter of the protein divided by the protein length

170. The total number of large hydrophobic residue blocks in the second quarter of the protein divided by the protein length

171. The total number of large hydrophobic residue blocks in the third quarter of the protein divided by the protein length

172. The total number of large hydrophobic residue blocks in the fourth quarter of the protein divided by the protein length

173. The total number of large hydrophobic residue blocks in the first quarter of the protein divided by the total number of large hydrophobic blocks of the protein

174. The total number of large hydrophobic residue blocks in the second quarter of the protein divided by the total number of large hydrophobic blocks of the protein

175. The total number of large hydrophobic residue blocks in the third quarter of the protein divided by the total number of large hydrophobic blocks of the protein

176. The total number of large hydrophobic residue blocks in the fourth quarter of the protein divided by the total number of large hydrophobic blocks of the protein

177. The total number of large hydrophobic residue blocks in the first half of the protein

178. The total number of large hydrophobic residue blocks in the three-quarter region of the protein

179. The total number of large hydrophobic residue blocks from position 25% to 75% of the protein

180. The total number of large hydrophobic residue blocks in the second half of the protein

181. The total number of large hydrophobic residue blocks in the first half of the protein divided by the protein length

182. The total number of large hydrophobic residue blocks in the three-quarter region of the protein divided by the protein length

183. The total number of large hydrophobic residue blocks from position 25% to 75% of the protein divided by the protein length

184. The total number of large hydrophobic residue blocks in the second half of the protein divided by the protein length

185. The total number of large hydrophobic residue blocks in the first half of the protein divided by the total number of large hydrophobic blocks of the protein

186. The total number of large hydrophobic residue blocks in the three-quarter region of the protein divided by the total number of large hydrophobic blocks of the protein

187. The total number of large hydrophobic residue blocks from position 25% to 75% of the protein divided by the total number of large hydrophobic blocks of the protein

188. The total number of large hydrophobic residue blocks in the second half of the protein divided by the total number of large hydrophobic blocks of the protein

189. The length of the maximum large hydrophobic block in the first quarter of the protein

190. The median of the large hydrophobic blocks in the first quarter of the protein

191. The mean of the large hydrophobic blocks in the first quarter of the protein

192. The length of the maximum large hydrophobic block in the second quarter of the protein

193. The median of the large hydrophobic blocks in the second quarter of the protein

194. The mean of the large hydrophobic blocks in the second quarter of the protein

195. The length of the maximum large hydrophobic block in the third quarter of the protein

196. The median of the large hydrophobic blocks in the third quarter of the protein

197. The mean of the large hydrophobic blocks in the third quarter of the protein

198. The length of the maximum large hydrophobic block in the fourth quarter of the protein

199. The median of the large hydrophobic blocks in the fourth quarter of the protein

200. The mean of the large hydrophobic blocks in the fourth quarter of the protein

201. The length of the maximum large hydrophobic block of the protein

202. The median of the large hydrophobic blocks of the protein

203. The mean of the large hydrophobic blocks of the protein

204. The total number of aliphatic residues in the sequence

205. The total number of aliphatic residues divided by the length of the protein

206. The total number of aliphatic residues in the first quarter of the sequence

207. The total number of aliphatic residues in the second quarter of the sequence

208. The total number of aliphatic residues in the third quarter of the sequence

209. The total number of aliphatic residues in the fourth quarter of the sequence

210. The total number of aliphatic residues in the first quarter of the sequence divided by the length of the protein

211. The total number of aliphatic residues in the second quarter of the sequence divided by the length of the protein

212. The total number of aliphatic residues in the third quarter of the sequence divided by the length of the protein

213. The total number of aliphatic residues in the fourth quarter of the sequence divided by the length of the protein

214. The total number of aliphatic residues in the first quarter of the sequence divided by the total number of aliphatic blocks

215. The total number of aliphatic residues in the first quarter of the sequence divided by the total number of aliphatic blocks

216. The total number of aliphatic residues in the first quarter of the sequence divided by the total number of aliphatic blocks

217. The total number of aliphatic residues in the first quarter of the sequence divided by the total number of aliphatic blocks

218. The total number of aliphatic residues in the first half of the protein

219. The total number of aliphatic residues in the three-quarter region of the protein

220. The total number of aliphatic residues from position 25% to 75% of the protein
221. The total number of aliphatic residues in the second half of the protein
222. The total number of aliphatic residues in the first half of the protein divided by the protein length
223. The total number of aliphatic residues in the three-quarter region of the protein divided by the protein length
224. The total number of aliphatic residues from position 25% to 75% of the protein divided by the protein length
225. The total number of aliphatic residues in the second half of the protein divided by the protein length
226. The total number of aliphatic residues in the first half of the protein divided by the total number of aliphatic residues in the protein
227. The total number of aliphatic residues in the three-quarter region of the protein divided by the total number of aliphatic residues in the protein
228. The total number of aliphatic residues from position 25% to 75% of the protein divided by the total number of aliphatic residues in the protein
229. The total number of aliphatic residues in the second half of the protein divided by the total number of aliphatic residues in the protein
230. The total number of aliphatic residue blocks in the protein
231. The total number of aliphatic residue blocks in the first quarter of the protein
232. The total number of aliphatic residue blocks in the second quarter of the protein
233. The total number of aliphatic residue blocks in the third quarter of the protein
234. The total number of aliphatic residue blocks in the fourth quarter of the protein
235. The total number of aliphatic residue blocks in the first quarter of the protein divided by the protein length
236. The total number of aliphatic residue blocks in the second quarter of the protein divided by the protein length
237. The total number of aliphatic residue blocks in the third quarter of the protein divided by the protein length
238. The total number of aliphatic residue blocks in the fourth quarter of the protein divided by the protein length
239. The total number of aliphatic residue blocks in the first quarter of the protein divided by the total number of aliphatic blocks of the protein

240. The total number of aliphatic residue blocks in the second quarter of the protein divided by the total number of aliphatic blocks of the protein

241. The total number of aliphatic residue blocks in the third quarter of the protein divided by the total number of aliphatic blocks of the protein

242. The total number of aliphatic residue blocks in the fourth quarter of the protein divided by the total number of aliphatic blocks of the protein

243. The total number of aliphatic residue blocks in the first half of the protein

244. The total number of aliphatic residue blocks in the three-quarter region of the protein

245. The total number of aliphatic residue blocks from position 25% to 75% of the protein

246. The total number of aliphatic residue blocks in the second half of the protein

247. The total number of aliphatic residue blocks in the first half of the protein divided by the protein length

248. The total number of aliphatic residue blocks in the three-quarter region of the protein divided by the protein length

249. The total number of aliphatic residue blocks from position 25% to 75% of the protein divided by the protein length

250. The total number of aliphatic residue blocks in the second half of the protein divided by the protein length

251. The total number of aliphatic residue blocks in the first half of the protein divided by the total number of aliphatic blocks of the protein

252. The total number of aliphatic residue blocks in the three-quarter region of the protein divided by the total number of aliphatic blocks of the protein

253. The total number of aliphatic residue blocks from position 25% to 75% of the protein divided by the total number of aliphatic blocks of the protein

254. The total number of aliphatic residue blocks in the second half of the protein divided by the total number of aliphatic blocks of the protein

255. The length of the maximum aliphatic block in the first quarter of the protein

256. The median of the aliphatic blocks in the first quarter of the protein

257. The mean of the aliphatic blocks in the first quarter of the protein

258. The length of the maximum aliphatic block in the second quarter of the protein

259. The median of the aliphatic blocks in the second quarter of the protein

260. The mean of the aliphatic blocks in the second quarter of the protein

261. The length of the maximum aliphatic block in the third quarter of the protein
262. The median of the aliphatic blocks in the third quarter of the protein
263. The mean of the aliphatic blocks in the third quarter of the protein
264. The length of the maximum aliphatic block in the fourth quarter of the protein
265. The median of the aliphatic blocks in the fourth quarter of the protein
266. The mean of the aliphatic blocks in the fourth quarter of the protein
267. The length of the maximum aliphatic block of the protein
268. The median of the aliphatic blocks of the protein
269. The mean of the aliphatic blocks of the protein
270. The total number of aromatic residues in the sequence
271. The total number of aromatic residues divided by the length of the protein
272. The total number of aromatic residues in the first quarter of the sequence
273. The total number of aromatic residues in the second quarter of the sequence
274. The total number of aromatic residues in the third quarter of the sequence
275. The total number of aromatic residues in the fourth quarter of the sequence
276. The total number of aromatic residues in the first quarter of the sequence divided by the length of the protein
277. The total number of aromatic residues in the second quarter of the sequence divided by the length of the protein
278. The total number of aromatic residues in the third quarter of the sequence divided by the length of the protein
279. The total number of aromatic residues in the fourth quarter of the sequence divided by the length of the protein
280. The total number of aromatic residues in the first quarter of the sequence divided by the total number of aromatic blocks
281. The total number of aromatic residues in the first quarter of the sequence divided by the total number of aromatic blocks
282. The total number of aromatic residues in the first quarter of the sequence divided by the total number of aromatic blocks
283. The total number of aromatic residues in the first quarter of the sequence divided by the total number of aromatic blocks
284. The total number of aromatic residues in the first half of the protein

285. The total number of aromatic residues in the three-quarter region of the protein

286. The total number of aromatic residues from position 25% to 75% of the protein

287. The total number of aromatic residues in the second half of the protein

288. The total number of aromatic residues in the first half of the protein divided by the protein length

289. The total number of aromatic residues in the three-quarter region of the protein divided by the protein length

290. The total number of aromatic residues from position 25% to 75% of the protein divided by the protein length

291. The total number of aromatic residues in the second half of the protein divided by the protein length

292. The total number of aromatic residues in the first half of the protein divided by the total number of aromatic residues in the protein

293. The total number of aromatic residues in the three-quarter region of the protein divided by the total number of aromatic residues in the protein

294. The total number of aromatic residues from position 25% to 75% of the protein divided by the total number of aromatic residues in the protein

295. The total number of aromatic residues in the second half of the protein divided by the total number of aromatic residues in the protein

296. The total number of aromatic residue blocks in the protein

297. The total number of aromatic residue blocks in the first quarter of the protein

298. The total number of aromatic residue blocks in the second quarter of the protein

299. The total number of aromatic residue blocks in the third quarter of the protein

300. The total number of aromatic residue blocks in the fourth quarter of the protein

301. The total number of aromatic residue blocks in the first quarter of the protein divided by the protein length

302. The total number of aromatic residue blocks in the second quarter of the protein divided by the protein length

303. The total number of aromatic residue blocks in the third quarter of the protein divided by the protein length

304. The total number of aromatic residue blocks in the fourth quarter of the protein divided by the protein length

305. The total number of aromatic residue blocks in the first quarter of the protein divided by the total number of aromatic blocks of the protein

306. The total number of aromatic residue blocks in the second quarter of the protein divided by the total number of aromatic blocks of the protein

307. The total number of aromatic residue blocks in the third quarter of the protein divided by the total number of aromatic blocks of the protein

308. The total number of aromatic residue blocks in the fourth quarter of the protein divided by the total number of aromatic blocks of the protein

309. The total number of aromatic residue blocks in the first half of the protein

310. The total number of aromatic residue blocks in the three-quarter region of the protein

311. The total number of aromatic residue blocks from position 25% to 75% of the protein

312. The total number of aromatic residue blocks in the second half of the protein

313. The total number of aromatic residue blocks in the first half of the protein divided by the protein length

314. The total number of aromatic residue blocks in the three-quarter region of the protein divided by the protein length

315. The total number of aromatic residue blocks from position 25% to 75% of the protein divided by the protein length

316. The total number of aromatic residue blocks in the second half of the protein divided by the protein length

317. The total number of aromatic residue blocks in the first half of the protein divided by the total number of aromatic blocks of the protein

318. The total number of aromatic residue blocks in the three-quarter region of the protein divided by the total number of aromatic blocks of the protein

319. The total number of aromatic residue blocks from position 25% to 75% of the protein divided by the total number of aromatic blocks of the protein

320. The total number of aromatic residue blocks in the second half of the protein divided by the total number of aromatic blocks of the protein

321. The length of the maximum aromatic block in the first quarter of the protein

322. The median of the aromatic blocks in the first quarter of the protein

323. The mean of the aromatic blocks in the first quarter of the protein

324. The length of the maximum aromatic block in the second quarter of the protein

325. The median of the aromatic blocks in the second quarter of the protein
326. The mean of the aromatic blocks in the second quarter of the protein
327. The length of the maximum aromatic block in the third quarter of the protein
328. The median of the aromatic blocks in the third quarter of the protein
329. The mean of the aromatic blocks in the third quarter of the protein
330. The length of the maximum aromatic block in the fourth quarter of the protein
331. The median of the aromatic blocks in the fourth quarter of the protein
332. The mean of the aromatic blocks in the fourth quarter of the protein
333. The length of the maximum aromatic block of the protein
334. The median of the aromatic blocks of the protein
335. The mean of the aromatic blocks of the protein
336. The total number of charged residues in the sequence
337. The total number of charged residues divided by the length of the protein
338. The total number of charged residues in the first quarter of the sequence
339. The total number of charged residues in the second quarter of the sequence
340. The total number of charged residues in the third quarter of the sequence
341. The total number of charged residues in the fourth quarter of the sequence
342. The total number of charged residues in the first quarter of the sequence divided by the length of the protein
343. The total number of charged residues in the second quarter of the sequence divided by the length of the protein
344. The total number of charged residues in the third quarter of the sequence divided by the length of the protein
345. The total number of charged residues in the fourth quarter of the sequence divided by the length of the protein
346. The total number of charged residues in the first quarter of the sequence divided by the total number of charged blocks
347. The total number of charged residues in the first quarter of the sequence divided by the total number of charged blocks
348. The total number of charged residues in the first quarter of the sequence divided by the total number of charged blocks

349. The total number of charged residues in the first quarter of the sequence divided by the total number of charged blocks

350. The total number of charged residues in the first half of the protein

351. The total number of charged residues in the three-quarter region of the protein

352. The total number of charged residues from position 25% to 75% of the protein

353. The total number of charged residues in the second half of the protein

354. The total number of charged residues in the first half of the protein divided by the protein length

355. The total number of charged residues in the three-quarter region of the protein divided by the protein length

356. The total number of charged residues from position 25% to 75% of the protein divided by the protein length

357. The total number of charged residues in the second half of the protein divided by the protein length

358. The total number of charged residues in the first half of the protein divided by the total number of charged residues in the protein

359. The total number of charged residues in the three-quarter region of the protein divided by the total number of charged residues in the protein

360. The total number of charged residues from position 25% to 75% of the protein divided by the total number of charged residues in the protein

361. The total number of charged residues in the second half of the protein divided by the total number of charged residues in the protein

362. The total number of charged residue blocks in the protein

363. The total number of charged residue blocks in the first quarter of the protein

364. The total number of charged residue blocks in the second quarter of the protein

365. The total number of charged residue blocks in the third quarter of the protein

366. The total number of charged residue blocks in the fourth quarter of the protein

367. The total number of charged residue blocks in the first quarter of the protein divided by the protein length

368. The total number of charged residue blocks in the second quarter of the protein divided by the protein length

369. The total number of charged residue blocks in the third quarter of the protein divided by the protein length
370. The total number of charged residue blocks in the fourth quarter of the protein divided by the protein length
371. The total number of charged residue blocks in the first quarter of the protein divided by the total number of charged blocks of the protein
372. The total number of charged residue blocks in the second quarter of the protein divided by the total number of charged blocks of the protein
373. The total number of charged residue blocks in the third quarter of the protein divided by the total number of charged blocks of the protein
374. The total number of charged residue blocks in the fourth quarter of the protein divided by the total number of charged blocks of the protein
375. The total number of charged residue blocks in the first half of the protein
376. The total number of charged residue blocks in the three-quarter region of the protein
377. The total number of charged residue blocks from position 25% to 75% of the protein
378. The total number of charged residue blocks in the second half of the protein
379. The total number of charged residue blocks in the first half of the protein divided by the protein length
380. The total number of charged residue blocks in the three-quarter region of the protein divided by the protein length
381. The total number of charged residue blocks from position 25% to 75% of the protein divided by the protein length
382. The total number of charged residue blocks in the second half of the protein divided by the protein length
383. The total number of charged residue blocks in the first half of the protein divided by the total number of charged blocks of the protein
384. The total number of charged residue blocks in the three-quarter region of the protein divided by the total number of charged blocks of the protein
385. The total number of charged residue blocks from position 25% to 75% of the protein divided by the total number of charged blocks of the protein
386. The total number of charged residue blocks in the second half of the protein divided by the total number of charged blocks of the protein
387. The length of the maximum charged block in the first quarter of the protein
388. The median of the charged blocks in the first quarter of the protein
389. The mean of the charged blocks in the first quarter of the protein

390. The length of the maximum charged block in the second quarter of the protein
391. The median of the charged blocks in the second quarter of the protein
392. The mean of the charged blocks in the second quarter of the protein
393. The length of the maximum charged block in the third quarter of the protein
394. The median of the charged blocks in the third quarter of the protein
395. The mean of the charged blocks in the third quarter of the protein
396. The length of the maximum charged block in the fourth quarter of the protein
397. The median of the charged blocks in the fourth quarter of the protein
398. The mean of the charged blocks in the fourth quarter of the protein
399. The length of the maximum charged block of the protein
400. The median of the charged blocks of the protein
401. The mean of the charged blocks of the protein
402. The total number of tiny residues in the sequence
403. The total number of tiny residues divided by the length of the protein
404. The total number of tiny residues in the first quarter of the sequence
405. The total number of tiny residues in the second quarter of the sequence
406. The total number of tiny residues in the third quarter of the sequence
407. The total number of tiny residues in the fourth quarter of the sequence
408. The total number of tiny residues in the first quarter of the sequence divided by the length of the protein
409. The total number of tiny residues in the second quarter of the sequence divided by the length of the protein
410. The total number of tiny residues in the third quarter of the sequence divided by the length of the protein
411. The total number of tiny residues in the fourth quarter of the sequence divided by the length of the protein
412. The total number of tiny residues in the first quarter of the sequence divided by the total number of tiny blocks
413. The total number of tiny residues in the first quarter of the sequence divided by the total number of tiny blocks

414. The total number of tiny residues in the first quarter of the sequence divided by the total number of tiny blocks

415. The total number of tiny residues in the first quarter of the sequence divided by the total number of tiny blocks

416. The total number of tiny residues in the first half of the protein

417. The total number of tiny residues in the three-quarter region of the protein

418. The total number of tiny residues from position 25% to 75% of the protein

419. The total number of tiny residues in the second half of the protein

420. The total number of tiny residues in the first half of the protein divided by the protein length

421. The total number of tiny residues in the three-quarter region of the protein divided by the protein length

422. The total number of tiny residues from position 25% to 75% of the protein divided by the protein length

423. The total number of tiny residues in the second half of the protein divided by the protein length

424. The total number of tiny residues in the first half of the protein divided by the total number of tiny residues in the protein

425. The total number of tiny residues in the three-quarter region of the protein divided by the total number of tiny residues in the protein

426. The total number of tiny residues from position 25% to 75% of the protein divided by the total number of tiny residues in the protein

427. The total number of tiny residues in the second half of the protein divided by the total number of tiny residues in the protein

428. The total number of tiny residue blocks in the protein

429. The total number of tiny residue blocks in the first quarter of the protein

430. The total number of tiny residue blocks in the second quarter of the protein

431. The total number of tiny residue blocks in the third quarter of the protein

432. The total number of tiny residue blocks in the fourth quarter of the protein

433. The total number of tiny residue blocks in the first quarter of the protein divided by the protein length

434. The total number of tiny residue blocks in the second quarter of the protein divided by the protein length

435. The total number of tiny residue blocks in the third quarter of the protein divided by the protein length

436. The total number of tiny residue blocks in the fourth quarter of the protein divided by the protein length

437. The total number of tiny residue blocks in the first quarter of the protein divided by the total number of tiny blocks of the protein

438. The total number of tiny residue blocks in the second quarter of the protein divided by the total number of tiny blocks of the protein

439. The total number of tiny residue blocks in the third quarter of the protein divided by the total number of tiny blocks of the protein

440. The total number of tiny residue blocks in the fourth quarter of the protein divided by the total number of tiny blocks of the protein

441. The total number of tiny residue blocks in the first half of the protein

442. The total number of tiny residue blocks in the three-quarter region of the protein

443. The total number of tiny residue blocks from position 25% to 75% of the protein

444. The total number of tiny residue blocks in the second half of the protein

445. The total number of tiny residue blocks in the first half of the protein divided by the protein length

446. The total number of tiny residue blocks in the three-quarter region of the protein divided by the protein length

447. The total number of tiny residue blocks from position 25% to 75% of the protein divided by the protein length

448. The total number of tiny residue blocks in the second half of the protein divided by the protein length

449. The total number of tiny residue blocks in the first half of the protein divided by the total number of tiny blocks of the protein

450. The total number of tiny residue blocks in the three-quarter region of the protein divided by the total number of tiny blocks of the protein

451. The total number of tiny residue blocks from position 25% to 75% of the protein divided by the total number of tiny blocks of the protein

452. The total number of tiny residue blocks in the second half of the protein divided by the total number of tiny blocks of the protein

453. The length of the maximum tiny block in the first quarter of the protein

454. The median of the tiny blocks in the first quarter of the protein

455. The mean of the tiny blocks in the first quarter of the protein

456. The length of the maximum tiny block in the second quarter of the protein

457. The median of the tiny blocks in the second quarter of the protein
458. The mean of the tiny blocks in the second quarter of the protein
459. The length of the maximum tiny block in the third quarter of the protein
460. The median of the tiny blocks in the third quarter of the protein
461. The mean of the tiny blocks in the third quarter of the protein
462. The length of the maximum tiny block in the fourth quarter of the protein
463. The median of the tiny blocks in the fourth quarter of the protein
464. The mean of the tiny blocks in the fourth quarter of the protein
465. The length of the maximum tiny block of the protein
466. The median of the tiny blocks of the protein
467. The mean of the tiny blocks of the protein
468. The total number of diverse residues in the sequence
469. The total number of diverse residues divided by the length of the protein
470. The total number of diverse residues in the first quarter of the sequence
471. The total number of diverse residues in the second quarter of the sequence
472. The total number of diverse residues in the third quarter of the sequence
473. The total number of diverse residues in the fourth quarter of the sequence
474. The total number of diverse residues in the first quarter of the sequence divided by the length of the protein
475. The total number of diverse residues in the second quarter of the sequence divided by the length of the protein
476. The total number of diverse residues in the third quarter of the sequence divided by the length of the protein
477. The total number of diverse residues in the fourth quarter of the sequence divided by the length of the protein
478. The total number of diverse residues in the first quarter of the sequence divided by the total number of diverse blocks
479. The total number of diverse residues in the first quarter of the sequence divided by the total number of diverse blocks
480. The total number of diverse residues in the first quarter of the sequence divided by the total number of diverse blocks
481. The total number of diverse residues in the first quarter of the sequence divided by the total number of diverse blocks
482. The total number of diverse residues in the first half of the protein

483. The total number of diverse residues in the three-quarter region of the protein

484. The total number of diverse residues from position 25% to 75% of the protein

485. The total number of diverse residues in the second half of the protein

486. The total number of diverse residues in the first half of the protein divided by the protein length

487. The total number of diverse residues in the three-quarter region of the protein divided by the protein length

488. The total number of diverse residues from position 25% to 75% of the protein divided by the protein length

489. The total number of diverse residues in the second half of the protein divided by the protein length

490. The total number of diverse residues in the first half of the protein divided by the total number of diverse residues in the protein

491. The total number of diverse residues in the three-quarter region of the protein divided by the total number of diverse residues in the protein

492. The total number of diverse residues from position 25% to 75% of the protein divided by the total number of diverse residues in the protein

493. The total number of diverse residues in the second half of the protein divided by the total number of diverse residues in the protein

494. The total number of diverse residue blocks in the protein

495. The total number of diverse residue blocks in the first quarter of the protein

496. The total number of diverse residue blocks in the second quarter of the protein

497. The total number of diverse residue blocks in the third quarter of the protein

498. The total number of diverse residue blocks in the fourth quarter of the protein

499. The total number of diverse residue blocks in the first quarter of the protein divided by the protein length

500. The total number of diverse residue blocks in the second quarter of the protein divided by the protein length

501. The total number of diverse residue blocks in the third quarter of the protein divided by the protein length

502. The total number of diverse residue blocks in the fourth quarter of the protein divided by the protein length

503. The total number of diverse residue blocks in the first quarter of the protein divided by the total number of diverse blocks of the protein

504. The total number of diverse residue blocks in the second quarter of the protein divided by the total number of diverse blocks of the protein

505. The total number of diverse residue blocks in the third quarter of the protein divided by the total number of diverse blocks of the protein

506. The total number of diverse residue blocks in the fourth quarter of the protein divided by the total number of diverse blocks of the protein

507. The total number of diverse residue blocks in the first half of the protein

508. The total number of diverse residue blocks in the three-quarter region of the protein

509. The total number of diverse residue blocks from position 25% to 75% of the protein

510. The total number of diverse residue blocks in the second half of the protein

511. The total number of diverse residue blocks in the first half of the protein divided by the protein length

512. The total number of diverse residue blocks in the three-quarter region of the protein divided by the protein length

513. The total number of diverse residue blocks from position 25% to 75% of the protein divided by the protein length

514. The total number of diverse residue blocks in the second half of the protein divided by the protein length

515. The total number of diverse residue blocks in the first half of the protein divided by the total number of diverse blocks of the protein

516. The total number of diverse residue blocks in the three-quarter region of the protein divided by the total number of diverse blocks of the protein

517. The total number of diverse residue blocks from position 25% to 75% of the protein divided by the total number of diverse blocks of the protein

518. The total number of diverse residue blocks in the second half of the protein divided by the total number of diverse blocks of the protein

519. The length of the maximum diverse block in the first quarter of the protein

520. The median of the diverse blocks in the first quarter of the protein

521. The mean of the diverse blocks in the first quarter of the protein

522. The length of the maximum diverse block in the second quarter of the protein

523. The median of the diverse blocks in the second quarter of the protein

524. The mean of the diverse blocks in the second quarter of the protein
525. The length of the maximum diverse block in the third quarter of the protein
526. The median of the diverse blocks in the third quarter of the protein
527. The mean of the diverse blocks in the third quarter of the protein
528. The length of the maximum diverse block in the fourth quarter of the protein
529. The median of the diverse blocks in the fourth quarter of the protein
530. The mean of the diverse blocks in the fourth quarter of the protein
531. The length of the maximum diverse block of the protein
532. The median of the diverse blocks of the protein
533. The mean of the diverse blocks of the protein
534. The total number of positively charged residues in the sequence
535. The total number of positively charged residues divided by the length of the protein
536. The total number of positively charged residues in the first quarter of the sequence
537. The total number of positively charged residues in the second quarter of the sequence
538. The total number of positively charged residues in the third quarter of the sequence
539. The total number of positively charged residues in the fourth quarter of the sequence
540. The total number of positively charged residues in the first quarter of the sequence divided by the length of the protein
541. The total number of positively charged residues in the second quarter of the sequence divided by the length of the protein
542. The total number of positively charged residues in the third quarter of the sequence divided by the length of the protein
543. The total number of positively charged residues in the fourth quarter of the sequence divided by the length of the protein
544. The total number of positively charged residues in the first quarter of the sequence divided by the total number of positively charged blocks
545. The total number of positively charged residues in the first quarter of the sequence divided by the total number of positively charged blocks

546. The total number of positively charged residues in the first quarter of the sequence divided by the total number of positively charged blocks

547. The total number of positively charged residues in the first quarter of the sequence divided by the total number of positively charged blocks

548. The total number of positively charged residues in the first half of the protein

549. The total number of positively charged residues in the three-quarter region of the protein

550. The total number of positively charged residues from position 25% to 75% of the protein

551. The total number of positively charged residues in the second half of the protein

552. The total number of positively charged residues in the first half of the protein divided by the protein length

553. The total number of positively charged residues in the three-quarter region of the protein divided by the protein length

554. The total number of positively charged residues from position 25% to 75% of the protein divided by the protein length

555. The total number of positively charged residues in the second half of the protein divided by the protein length

556. The total number of positively charged residues in the first half of the protein divided by the total number of positively charged residues in the protein

557. The total number of positively charged residues in the three-quarter region of the protein divided by the total number of positively charged residues in the protein

558. The total number of positively charged residues from position 25% to 75% of the protein divided by the total number of positively charged residues in the protein

559. The total number of positively charged residues in the second half of the protein divided by the total number of positively charged residues in the protein

560. The total number of positively charged residue blocks in the protein

561. The total number of positively charged residue blocks in the first quarter of the protein

562. The total number of positively charged residue blocks in the second quarter of the protein

563. The total number of positively charged residue blocks in the third quarter of the protein

564. The total number of positively charged residue blocks in the fourth quarter of the protein

565. The total number of positively charged residue blocks in the first quarter of the protein divided by the protein length

566. The total number of positively charged residue blocks in the second quarter of the protein divided by the protein length

567. The total number of positively charged residue blocks in the third quarter of the protein divided by the protein length

568. The total number of positively charged residue blocks in the fourth quarter of the protein divided by the protein length

569. The total number of positively charged residue blocks in the first quarter of the protein divided by the total number of positively charged blocks of the protein

570. The total number of positively charged residue blocks in the second quarter of the protein divided by the total number of positively charged blocks of the protein

571. The total number of positively charged residue blocks in the third quarter of the protein divided by the total number of positively charged blocks of the protein

572. The total number of positively charged residue blocks in the fourth quarter of the protein divided by the total number of positively charged blocks of the protein

573. The total number of positively charged residue blocks in the first half of the protein

574. The total number of positively charged residue blocks in the three-quarter region of the protein

575. The total number of positively charged residue blocks from position 25% to 75% of the protein

576. The total number of positively charged residue blocks in the second half of the protein

577. The total number of positively charged residue blocks in the first half of the protein divided by the protein length

578. The total number of positively charged residue blocks in the three-quarter region of the protein divided by the protein length

579. The total number of positively charged residue blocks from position 25% to 75% of the protein divided by the protein length

580. The total number of positively charged residue blocks in the second half of the protein divided by the protein length

581. The total number of positively charged residue blocks in the first half of the protein divided by the total number of positively charged blocks of the protein

582. The total number of positively charged residue blocks in the three-quarter region of the protein divided by the total number of positively charged blocks of the protein

583. The total number of positively charged residue blocks from position 25% to 75% of the protein divided by the total number of positively charged blocks of the protein

584. The total number of positively charged residue blocks in the second half of the protein divided by the total number of positively charged blocks of the protein

585. The length of the maximum positively charged block in the first quarter of the protein

586. The median of the positively charged blocks in the first quarter of the protein

587. The mean of the positively charged blocks in the first quarter of the protein

588. The length of the maximum positively charged block in the second quarter of the protein

589. The median of the positively charged blocks in the second quarter of the protein

590. The mean of the positively charged blocks in the second quarter of the protein

591. The length of the maximum positively charged block in the third quarter of the protein

592. The median of the positively charged blocks in the third quarter of the protein

593. The mean of the positively charged blocks in the third quarter of the protein

594. The length of the maximum positively charged block in the fourth quarter of the protein

595. The median of the positively charged blocks in the fourth quarter of the protein

596. The mean of the positively charged blocks in the fourth quarter of the protein

597. The length of the maximum positively charged block of the protein

598. The median of the positively charged blocks of the protein

599. The mean of the positively charged blocks of the protein

600. The total number of negatively charged residues in the sequence

601. The total number of negatively charged residues divided by the length of the protein

602. The total number of negatively charged residues in the first quarter of the sequence

603. The total number of negatively charged residues in the second quarter of the sequence

604. The total number of negatively charged residues in the third quarter of the sequence

605. The total number of negatively charged residues in the fourth quarter of the sequence

606. The total number of negatively charged residues in the first quarter of the sequence divided by the length of the protein

607. The total number of negatively charged residues in the second quarter of the sequence divided by the length of the protein

608. The total number of negatively charged residues in the third quarter of the sequence divided by the length of the protein

609. The total number of negatively charged residues in the fourth quarter of the sequence divided by the length of the protein

610. The total number of negatively charged residues in the first quarter of the sequence divided by the total number of negatively charged blocks

611. The total number of negatively charged residues in the first quarter of the sequence divided by the total number of negatively charged blocks

612. The total number of negatively charged residues in the first quarter of the sequence divided by the total number of negatively charged blocks

613. The total number of negatively charged residues in the first quarter of the sequence divided by the total number of negatively charged blocks

614. The total number of negatively charged residues in the first half of the protein

615. The total number of negatively charged residues in the three-quarter region of the protein

616. The total number of negatively charged residues from position 25% to 75% of the protein

617. The total number of negatively charged residues in the second half of the protein

618. The total number of negatively charged residues in the first half of the protein divided by the protein length

619. The total number of negatively charged residues in the three-quarter region of the protein divided by the protein length

620. The total number of negatively charged residues from position 25% to 75% of the protein divided by the protein length

621. The total number of negatively charged residues in the second half of the protein divided by the protein length

622. The total number of negatively charged residues in the first half of the protein divided by the total number of negatively charged residues in the protein

623. The total number of negatively charged residues in the three-quarter region of the protein divided by the total number of negatively charged residues in the protein

624. The total number of negatively charged residues from position 25% to 75% of the protein divided by the total number of negatively charged residues in the protein

625. The total number of negatively charged residues in the second half of the protein divided by the total number of negatively charged residues in the protein

626. The total number of negatively charged residue blocks in the protein

627. The total number of negatively charged residue blocks in the first quarter of the protein

628. The total number of negatively charged residue blocks in the second quarter of the protein

629. The total number of negatively charged residue blocks in the third quarter of the protein

630. The total number of negatively charged residue blocks in the fourth quarter of the protein

631. The total number of negatively charged residue blocks in the first quarter of the protein divided by the protein length

632. The total number of negatively charged residue blocks in the second quarter of the protein divided by the protein length

633. The total number of negatively charged residue blocks in the third quarter of the protein divided by the protein length

634. The total number of negatively charged residue blocks in the fourth quarter of the protein divided by the protein length

635. The total number of negatively charged residue blocks in the first quarter of the protein divided by the total number of negatively charged blocks of the protein

636. The total number of negatively charged residue blocks in the second quarter of the protein divided by the total number of negatively charged blocks of the protein

637. The total number of negatively charged residue blocks in the third quarter of the protein divided by the total number of negatively charged blocks of the protein

638. The total number of negatively charged residue blocks in the fourth quarter of the protein divided by the total number of negatively charged blocks of the protein

639. The total number of negatively charged residue blocks in the first half of the protein

640. The total number of negatively charged residue blocks in the three-quarter region of the protein

641. The total number of negatively charged residue blocks from position 25% to 75% of the protein

642. The total number of negatively charged residue blocks in the second half of the protein

643. The total number of negatively charged residue blocks in the first half of the protein divided by the protein length

644. The total number of negatively charged residue blocks in the three-quarter region of the protein divided by the protein length

645. The total number of negatively charged residue blocks from position 25% to 75% of the protein divided by the protein length

646. The total number of negatively charged residue blocks in the second half of the protein divided by the protein length

647. The total number of negatively charged residue blocks in the first half of the protein divided by the total number of negatively charged blocks of the protein

648. The total number of negatively charged residue blocks in the three-quarter region of the protein divided by the total number of negatively charged blocks of the protein

649. The total number of negatively charged residue blocks from position 25% to 75% of the protein divided by the total number of negatively charged blocks of the protein

650. The total number of negatively charged residue blocks in the second half of the protein divided by the total number of negatively charged blocks of the protein

651. The length of the maximum negatively charged block in the first quarter of the protein

652. The median of the negatively charged blocks in the first quarter of the protein

653. The mean of the negatively charged blocks in the first quarter of the protein

654. The length of the maximum negatively charged block in the second quarter of the protein

655. The median of the negatively charged blocks in the second quarter of the protein

656. The mean of the negatively charged blocks in the second quarter of the protein

657. The length of the maximum negatively charged block in the third quarter of the protein

658. The median of the negatively charged blocks in the third quarter of the protein

659. The mean of the negatively charged blocks in the third quarter of the protein

660. The length of the maximum negatively charged block in the fourth quarter of the protein

661. The median of the negatively charged blocks in the fourth quarter of the protein

662. The mean of the negatively charged blocks in the fourth quarter of the protein

663. The length of the maximum negatively charged block of the protein

664. The median of the negatively charged blocks of the protein

665. The mean of the negatively charged blocks of the protein

666. The total number of polar residues in the sequence

667. The total number of polar residues divided by the length of the protein

668. The total number of polar residues in the first quarter of the sequence

669. The total number of polar residues in the second quarter of the sequence

670. The total number of polar residues in the third quarter of the sequence

671. The total number of polar residues in the fourth quarter of the sequence

672. The total number of polar residues in the first quarter of the sequence divided by the length of the protein

673. The total number of polar residues in the second quarter of the sequence divided by the length of the protein

674. The total number of polar residues in the third quarter of the sequence divided by the length of the protein

675. The total number of polar residues in the fourth quarter of the sequence divided by the length of the protein

676. The total number of polar residues in the first quarter of the sequence divided by the total number of polar blocks

677. The total number of polar residues in the first quarter of the sequence divided by the total number of polar blocks

678. The total number of polar residues in the first quarter of the sequence divided by the total number of polar blocks

679. The total number of polar residues in the first quarter of the sequence divided by the total number of polar blocks

680. The total number of polar residues in the first half of the protein

681. The total number of polar residues in the three-quarter region of the protein

682. The total number of polar residues from position 25% to 75% of the protein

683. The total number of polar residues in the second half of the protein

684. The total number of polar residues in the first half of the protein divided by the protein length

685. The total number of polar residues in the three-quarter region of the protein divided by the protein length

686. The total number of polar residues from position 25% to 75% of the protein divided by the protein length

687. The total number of polar residues in the second half of the protein divided by the protein length

688. The total number of polar residues in the first half of the protein divided by the total number of polar residues in the protein

689. The total number of polar residues in the three-quarter region of the protein divided by the total number of polar residues in the protein

690. The total number of polar residues from position 25% to 75% of the protein divided by the total number of polar residues in the protein

691. The total number of polar residues in the second half of the protein divided by the total number of polar residues in the protein

692. The total number of polar residue blocks in the protein

693. The total number of polar residue blocks in the first quarter of the protein

694. The total number of polar residue blocks in the second quarter of the protein

695. The total number of polar residue blocks in the third quarter of the protein
696. The total number of polar residue blocks in the fourth quarter of the protein
697. The total number of polar residue blocks in the first quarter of the protein divided by the protein length
698. The total number of polar residue blocks in the second quarter of the protein divided by the protein length
699. The total number of polar residue blocks in the third quarter of the protein divided by the protein length
700. The total number of polar residue blocks in the fourth quarter of the protein divided by the protein length
701. The total number of polar residue blocks in the first quarter of the protein divided by the total number of polar blocks of the protein
702. The total number of polar residue blocks in the second quarter of the protein divided by the total number of polar blocks of the protein
703. The total number of polar residue blocks in the third quarter of the protein divided by the total number of polar blocks of the protein
704. The total number of polar residue blocks in the fourth quarter of the protein divided by the total number of polar blocks of the protein
705. The total number of polar residue blocks in the first half of the protein
706. The total number of polar residue blocks in the three-quarter region of the protein
707. The total number of polar residue blocks from position 25% to 75% of the protein
708. The total number of polar residue blocks in the second half of the protein
709. The total number of polar residue blocks in the first half of the protein divided by the protein length
710. The total number of polar residue blocks in the three-quarter region of the protein divided by the protein length
711. The total number of polar residue blocks from position 25% to 75% of the protein divided by the protein length
712. The total number of polar residue blocks in the second half of the protein divided by the protein length
713. The total number of polar residue blocks in the first half of the protein divided by the total number of polar blocks of the protein
714. The total number of polar residue blocks in the three-quarter region of the protein divided by the total number of polar blocks of the protein

715. The total number of polar residue blocks from position 25% to 75% of the protein divided by the total number of polar blocks of the protein

716. The total number of polar residue blocks in the second half of the protein divided by the total number of polar blocks of the protein

717. The length of the maximum polar block in the first quarter of the protein

718. The median of the polar blocks in the first quarter of the protein

719. The mean of the polar blocks in the first quarter of the protein

720. The length of the maximum polar block in the second quarter of the protein

721. The median of the polar blocks in the second quarter of the protein

722. The mean of the polar blocks in the second quarter of the protein

723. The length of the maximum polar block in the third quarter of the protein

724. The median of the polar blocks in the third quarter of the protein

725. The mean of the polar blocks in the third quarter of the protein

726. The length of the maximum polar block in the fourth quarter of the protein

727. The median of the polar blocks in the fourth quarter of the protein

728. The mean of the polar blocks in the fourth quarter of the protein

729. The length of the maximum polar block of the protein

730. The median of the polar blocks of the protein

731. The mean of the polar blocks of the protein

732. The total number of amides residues in the sequence

733. The total number of amides residues divided by the length of the protein

734. The total number of amides residues in the first quarter of the sequence

735. The total number of amides residues in the second quarter of the sequence

736. The total number of amides residues in the third quarter of the sequence

737. The total number of amides residues in the fourth quarter of the sequence

738. The total number of amides residues in the first quarter of the sequence divided by the length of the protein

739. The total number of amides residues in the second quarter of the sequence divided by the length of the protein

740. The total number of amides residues in the third quarter of the sequence divided by the length of the protein

741. The total number of amides residues in the fourth quarter of the sequence divided by the length of the protein

742. The total number of amides residues in the first quarter of the sequence divided by the total number of amides blocks

743. The total number of amides residues in the first quarter of the sequence divided by the total number of amides blocks

744. The total number of amides residues in the first quarter of the sequence divided by the total number of amides blocks

745. The total number of amides residues in the first quarter of the sequence divided by the total number of amides blocks

746. The total number of amides residues in the first half of the protein

747. The total number of amides residues in the three-quarter region of the protein

748. The total number of amides residues from position 25% to 75% of the protein

749. The total number of amides residues in the second half of the protein

750. The total number of amides residues in the first half of the protein divided by the protein length

751. The total number of amides residues in the three-quarter region of the protein divided by the protein length

752. The total number of amides residues from position 25% to 75% of the protein divided by the protein length

753. The total number of amides residues in the second half of the protein divided by the protein length

754. The total number of amides residues in the first half of the protein divided by the total number of amides residues in the protein

755. The total number of amides residues in the three-quarter region of the protein divided by the total number of amides residues in the protein

756. The total number of amides residues from position 25% to 75% of the protein divided by the total number of amides residues in the protein

757. The total number of amides residues in the second half of the protein divided by the total number of amides residues in the protein

758. The total number of amides residue blocks in the protein

759. The total number of amides residue blocks in the first quarter of the protein

760. The total number of amides residue blocks in the second quarter of the protein

761. The total number of amides residue blocks in the third quarter of the protein
762. The total number of amides residue blocks in the fourth quarter of the protein
763. The total number of amides residue blocks in the first quarter of the protein divided by the protein length
764. The total number of amides residue blocks in the second quarter of the protein divided by the protein length
765. The total number of amides residue blocks in the third quarter of the protein divided by the protein length
766. The total number of amides residue blocks in the fourth quarter of the protein divided by the protein length
767. The total number of amides residue blocks in the first quarter of the protein divided by the total number of amides blocks of the protein
768. The total number of amides residue blocks in the second quarter of the protein divided by the total number of amides blocks of the protein
769. The total number of amides residue blocks in the third quarter of the protein divided by the total number of amides blocks of the protein
770. The total number of amides residue blocks in the fourth quarter of the protein divided by the total number of amides blocks of the protein
771. The total number of amides residue blocks in the first half of the protein
772. The total number of amides residue blocks in the three-quarter region of the protein
773. The total number of amides residue blocks from position 25% to 75% of the protein
774. The total number of amides residue blocks in the second half of the protein
775. The total number of amides residue blocks in the first half of the protein divided by the protein length
776. The total number of amides residue blocks in the three-quarter region of the protein divided by the protein length
777. The total number of amides residue blocks from position 25% to 75% of the protein divided by the protein length
778. The total number of amides residue blocks in the second half of the protein divided by the protein length
779. The total number of amides residue blocks in the first half of the protein divided by the total number of amides blocks of the protein
780. The total number of amides residue blocks in the three-quarter region of the protein divided by the total number of amides blocks of the protein

781. The total number of amides residue blocks from position 25% to 75% of the protein divided by the total number of amides blocks of the protein

782. The total number of amides residue blocks in the second half of the protein divided by the total number of amides blocks of the protein

783. The length of the maximum amides block in the first quarter of the protein

784. The median of the amides blocks in the first quarter of the protein

785. The mean of the amides blocks in the first quarter of the protein

786. The length of the maximum amides block in the second quarter of the protein

787. The median of the amides blocks in the second quarter of the protein

788. The mean of the amides blocks in the second quarter of the protein

789. The length of the maximum amides block in the third quarter of the protein

790. The median of the amides blocks in the third quarter of the protein

791. The mean of the amides blocks in the third quarter of the protein

792. The length of the maximum amides block in the fourth quarter of the protein

793. The median of the amides blocks in the fourth quarter of the protein

794. The mean of the amides blocks in the fourth quarter of the protein

795. The length of the maximum amides block of the protein

796. The median of the amides blocks of the protein

797. The mean of the amides blocks of the protein

798. The total number of alanine residues in the sequence

799. The total number of alanine residues divided by the length of the protein

800. The total number of alanine residues in the first quarter of the sequence

801. The total number of alanine residues in the second quarter of the sequence

802. The total number of alanine residues in the third quarter of the sequence

803. The total number of alanine residues in the fourth quarter of the sequence

804. The total number of alanine residues in the first quarter of the sequence divided by the length of the protein

805. The total number of alanine residues in the second quarter of the sequence divided by the length of the protein

806. The total number of alanine residues in the third quarter of the sequence divided by the length of the protein

807. The total number of alanine residues in the fourth quarter of the sequence divided by the length of the protein

808. The total number of alanine residues in the first quarter of the sequence divided by the total number of alanine blocks

809. The total number of alanine residues in the first quarter of the sequence divided by the total number of alanine blocks

810. The total number of alanine residues in the first quarter of the sequence divided by the total number of alanine blocks

811. The total number of alanine residues in the first quarter of the sequence divided by the total number of alanine blocks

812. The total number of alanine residues in the first half of the protein

813. The total number of alanine residues in the three-quarter region of the protein

814. The total number of alanine residues from position 25% to 75% of the protein

815. The total number of alanine residues in the second half of the protein

816. The total number of alanine residues in the first half of the protein divided by the protein length

817. The total number of alanine residues in the three-quarter region of the protein divided by the protein length

818. The total number of alanine residues from position 25% to 75% of the protein divided by the protein length

819. The total number of alanine residues in the second half of the protein divided by the protein length

820. The total number of alanine residues in the first half of the protein divided by the total number of alanine residues in the protein

821. The total number of alanine residues in the three-quarter region of the protein divided by the total number of alanine residues in the protein

822. The total number of alanine residues from position 25% to 75% of the protein divided by the total number of alanine residues in the protein

823. The total number of alanine residues in the second half of the protein divided by the total number of alanine residues in the protein

824. The total number of alanine residue blocks in the protein

825. The total number of alanine residue blocks in the first quarter of the protein

826. The total number of alanine residue blocks in the second quarter of the protein

827. The total number of alanine residue blocks in the third quarter of the protein
828. The total number of alanine residue blocks in the fourth quarter of the protein
829. The total number of alanine residue blocks in the first quarter of the protein divided by the protein length
830. The total number of alanine residue blocks in the second quarter of the protein divided by the protein length
831. The total number of alanine residue blocks in the third quarter of the protein divided by the protein length
832. The total number of alanine residue blocks in the fourth quarter of the protein divided by the protein length
833. The total number of alanine residue blocks in the first quarter of the protein divided by the total number of alanine blocks of the protein
834. The total number of alanine residue blocks in the second quarter of the protein divided by the total number of alanine blocks of the protein
835. The total number of alanine residue blocks in the third quarter of the protein divided by the total number of alanine blocks of the protein
836. The total number of alanine residue blocks in the fourth quarter of the protein divided by the total number of alanine blocks of the protein
837. The total number of alanine residue blocks in the first half of the protein
838. The total number of alanine residue blocks in the three-quarter region of the protein
839. The total number of alanine residue blocks from position 25% to 75% of the protein
840. The total number of alanine residue blocks in the second half of the protein
841. The total number of alanine residue blocks in the first half of the protein divided by the protein length
842. The total number of alanine residue blocks in the three-quarter region of the protein divided by the protein length
843. The total number of alanine residue blocks from position 25% to 75% of the protein divided by the protein length
844. The total number of alanine residue blocks in the second half of the protein divided by the protein length
845. The total number of alanine residue blocks in the first half of the protein divided by the total number of alanine blocks of the protein
846. The total number of alanine residue blocks in the three-quarter region of the protein divided by the total number of alanine blocks of the protein

847. The total number of alanine residue blocks from position 25% to 75% of the protein divided by the total number of alanine blocks of the protein

848. The total number of alanine residue blocks in the second half of the protein divided by the total number of alanine blocks of the protein

849. The length of the maximum alanine block in the first quarter of the protein

850. The median of the alanine blocks in the first quarter of the protein

851. The mean of the alanine blocks in the first quarter of the protein

852. The length of the maximum alanine block in the second quarter of the protein

853. The median of the alanine blocks in the second quarter of the protein

854. The mean of the alanine blocks in the second quarter of the protein

855. The length of the maximum alanine block in the third quarter of the protein

856. The median of the alanine blocks in the third quarter of the protein

857. The mean of the alanine blocks in the third quarter of the protein

858. The length of the maximum alanine block in the fourth quarter of the protein

859. The median of the alanine blocks in the fourth quarter of the protein

860. The mean of the alanine blocks in the fourth quarter of the protein

861. The length of the maximum alanine block of the protein

862. The median of the alanine blocks of the protein

863. The mean of the alanine blocks of the protein

864. The total number of cysteine residues in the sequence

865. The total number of cysteine residues divided by the length of the protein

866. The total number of cysteine residues in the first quarter of the sequence

867. The total number of cysteine residues in the second quarter of the sequence

868. The total number of cysteine residues in the third quarter of the sequence

869. The total number of cysteine residues in the fourth quarter of the sequence

870. The total number of cysteine residues in the first quarter of the sequence divided by the length of the protein

871. The total number of cysteine residues in the second quarter of the sequence divided by the length of the protein

872. The total number of cysteine residues in the third quarter of the sequence divided by the length of the protein

873. The total number of cysteine residues in the fourth quarter of the sequence divided by the length of the protein

874. The total number of cysteine residues in the first quarter of the sequence divided by the total number of cysteine blocks

875. The total number of cysteine residues in the first quarter of the sequence divided by the total number of cysteine blocks

876. The total number of cysteine residues in the first quarter of the sequence divided by the total number of cysteine blocks

877. The total number of cysteine residues in the first quarter of the sequence divided by the total number of cysteine blocks

878. The total number of cysteine residues in the first half of the protein

879. The total number of cysteine residues in the three-quarter region of the protein

880. The total number of cysteine residues from position 25% to 75% of the protein

881. The total number of cysteine residues in the second half of the protein

882. The total number of cysteine residues in the first half of the protein divided by the protein length

883. The total number of cysteine residues in the three-quarter region of the protein divided by the protein length

884. The total number of cysteine residues from position 25% to 75% of the protein divided by the protein length

885. The total number of cysteine residues in the second half of the protein divided by the protein length

886. The total number of cysteine residues in the first half of the protein divided by the total number of cysteine residues in the protein

887. The total number of cysteine residues in the three-quarter region of the protein divided by the total number of cysteine residues in the protein

888. The total number of cysteine residues from position 25% to 75% of the protein divided by the total number of cysteine residues in the protein

889. The total number of cysteine residues in the second half of the protein divided by the total number of cysteine residues in the protein

890. The total number of cysteine residue blocks in the protein

891. The total number of cysteine residue blocks in the first quarter of the protein

892. The total number of cysteine residue blocks in the second quarter of the protein

893. The total number of cysteine residue blocks in the third quarter of the protein

894. The total number of cysteine residue blocks in the fourth quarter of the protein

895. The total number of cysteine residue blocks in the first quarter of the protein divided by the protein length

896. The total number of cysteine residue blocks in the second quarter of the protein divided by the protein length

897. The total number of cysteine residue blocks in the third quarter of the protein divided by the protein length

898. The total number of cysteine residue blocks in the fourth quarter of the protein divided by the protein length

899. The total number of cysteine residue blocks in the first quarter of the protein divided by the total number of cysteine blocks of the protein

900. The total number of cysteine residue blocks in the second quarter of the protein divided by the total number of cysteine blocks of the protein

901. The total number of cysteine residue blocks in the third quarter of the protein divided by the total number of cysteine blocks of the protein

902. The total number of cysteine residue blocks in the fourth quarter of the protein divided by the total number of cysteine blocks of the protein

903. The total number of cysteine residue blocks in the first half of the protein

904. The total number of cysteine residue blocks in the three-quarter region of the protein

905. The total number of cysteine residue blocks from position 25% to 75% of the protein

906. The total number of cysteine residue blocks in the second half of the protein

907. The total number of cysteine residue blocks in the first half of the protein divided by the protein length

908. The total number of cysteine residue blocks in the three-quarter region of the protein divided by the protein length

909. The total number of cysteine residue blocks from position 25% to 75% of the protein divided by the protein length

910. The total number of cysteine residue blocks in the second half of the protein divided by the protein length

911. The total number of cysteine residue blocks in the first half of the protein divided by the total number of cysteine blocks of the protein

912. The total number of cysteine residue blocks in the three-quarter region of the protein divided by the total number of cysteine blocks of the protein

913. The total number of cysteine residue blocks from position 25% to 75% of the protein divided by the total number of cysteine blocks of the protein

914. The total number of cysteine residue blocks in the second half of the protein divided by the total number of cysteine blocks of the protein

915. The length of the maximum cysteine block in the first quarter of the protein

916. The median of the cysteine blocks in the first quarter of the protein

917. The mean of the cysteine blocks in the first quarter of the protein

918. The length of the maximum cysteine block in the second quarter of the protein

919. The median of the cysteine blocks in the second quarter of the protein

920. The mean of the cysteine blocks in the second quarter of the protein

921. The length of the maximum cysteine block in the third quarter of the protein

922. The median of the cysteine blocks in the third quarter of the protein

923. The mean of the cysteine blocks in the third quarter of the protein

924. The length of the maximum cysteine block in the fourth quarter of the protein

925. The median of the cysteine blocks in the fourth quarter of the protein

926. The mean of the cysteine blocks in the fourth quarter of the protein

927. The length of the maximum cysteine block of the protein

928. The median of the cysteine blocks of the protein

929. The mean of the cysteine blocks of the protein

930. The total number of aspartic acid residues in the sequence

931. The total number of aspartic acid residues divided by the length of the protein

932. The total number of aspartic acid residues in the first quarter of the sequence

933. The total number of aspartic acid residues in the second quarter of the sequence

934. The total number of aspartic acid residues in the third quarter of the sequence

935. The total number of aspartic acid residues in the fourth quarter of the sequence

936. The total number of aspartic acid residues in the first quarter of the sequence divided by the length of the protein

937. The total number of aspartic acid residues in the second quarter of the sequence divided by the length of the protein

938. The total number of aspartic acid residues in the third quarter of the sequence divided by the length of the protein

939. The total number of aspartic acid residues in the fourth quarter of the sequence divided by the length of the protein

940. The total number of aspartic acid residues in the first quarter of the sequence divided by the total number of aspartic acid blocks

941. The total number of aspartic acid residues in the first quarter of the sequence divided by the total number of aspartic acid blocks

942. The total number of aspartic acid residues in the first quarter of the sequence divided by the total number of aspartic acid blocks

943. The total number of aspartic acid residues in the first quarter of the sequence divided by the total number of aspartic acid blocks

944. The total number of aspartic acid residues in the first half of the protein

945. The total number of aspartic acid residues in the three-quarter region of the protein

946. The total number of aspartic acid residues from position 25% to 75% of the protein

947. The total number of aspartic acid residues in the second half of the protein

948. The total number of aspartic acid residues in the first half of the protein divided by the protein length

949. The total number of aspartic acid residues in the three-quarter region of the protein divided by the protein length

950. The total number of aspartic acid residues from position 25% to 75% of the protein divided by the protein length

951. The total number of aspartic acid residues in the second half of the protein divided by the protein length

952. The total number of aspartic acid residues in the first half of the protein divided by the total number of aspartic acid residues in the protein

953. The total number of aspartic acid residues in the three-quarter region of the protein divided by the total number of aspartic acid residues in the protein

954. The total number of aspartic acid residues from position 25% to 75% of the protein divided by the total number of aspartic acid residues in the protein

955. The total number of aspartic acid residues in the second half of the protein divided by the total number of aspartic acid residues in the protein

956. The total number of aspartic acid residue blocks in the protein

957. The total number of aspartic acid residue blocks in the first quarter of the protein

958. The total number of aspartic acid residue blocks in the second quarter of the protein

959. The total number of aspartic acid residue blocks in the third quarter of the protein

960. The total number of aspartic acid residue blocks in the fourth quarter of the protein

961. The total number of aspartic acid residue blocks in the first quarter of the protein divided by the protein length

962. The total number of aspartic acid residue blocks in the second quarter of the protein divided by the protein length

963. The total number of aspartic acid residue blocks in the third quarter of the protein divided by the protein length

964. The total number of aspartic acid residue blocks in the fourth quarter of the protein divided by the protein length

965. The total number of aspartic acid residue blocks in the first quarter of the protein divided by the total number of aspartic acid blocks of the protein

966. The total number of aspartic acid residue blocks in the second quarter of the protein divided by the total number of aspartic acid blocks of the protein

967. The total number of aspartic acid residue blocks in the third quarter of the protein divided by the total number of aspartic acid blocks of the protein

968. The total number of aspartic acid residue blocks in the fourth quarter of the protein divided by the total number of aspartic acid blocks of the protein

969. The total number of aspartic acid residue blocks in the first half of the protein

970. The total number of aspartic acid residue blocks in the three-quarter region of the protein

971. The total number of aspartic acid residue blocks from position 25% to 75% of the protein

972. The total number of aspartic acid residue blocks in the second half of the protein
973. The total number of aspartic acid residue blocks in the first half of the protein divided by the protein length
974. The total number of aspartic acid residue blocks in the three-quarter region of the protein divided by the protein length
975. The total number of aspartic acid residue blocks from position 25% to 75% of the protein divided by the protein length
976. The total number of aspartic acid residue blocks in the second half of the protein divided by the protein length
977. The total number of aspartic acid residue blocks in the first half of the protein divided by the total number of aspartic acid blocks of the protein
978. The total number of aspartic acid residue blocks in the three-quarter region of the protein divided by the total number of aspartic acid blocks of the protein
979. The total number of aspartic acid residue blocks from position 25% to 75% of the protein divided by the total number of aspartic acid blocks of the protein
980. The total number of aspartic acid residue blocks in the second half of the protein divided by the total number of aspartic acid blocks of the protein
981. The length of the maximum aspartic acid block in the first quarter of the protein
982. The median of the aspartic acid blocks in the first quarter of the protein
983. The mean of the aspartic acid blocks in the first quarter of the protein
984. The length of the maximum aspartic acid block in the second quarter of the protein
985. The median of the aspartic acid blocks in the second quarter of the protein
986. The mean of the aspartic acid blocks in the second quarter of the protein
987. The length of the maximum aspartic acid block in the third quarter of the protein
988. The median of the aspartic acid blocks in the third quarter of the protein
989. The mean of the aspartic acid blocks in the third quarter of the protein
990. The length of the maximum aspartic acid block in the fourth quarter of the protein
991. The median of the aspartic acid blocks in the fourth quarter of the protein
992. The mean of the aspartic acid blocks in the fourth quarter of the protein

993. The length of the maximum aspartic acid block of the protein
994. The median of the aspartic acid blocks of the protein
995. The mean of the aspartic acid blocks of the protein
996. The total number of glutamic acid residues in the sequence
997. The total number of glutamic acid residues divided by the length of the protein
998. The total number of glutamic acid residues in the first quarter of the sequence
999. The total number of glutamic acid residues in the second quarter of the sequence
1000. The total number of glutamic acid residues in the third quarter of the sequence
1001. The total number of glutamic acid residues in the fourth quarter of the sequence
1002. The total number of glutamic acid residues in the first quarter of the sequence divided by the length of the protein
1003. The total number of glutamic acid residues in the second quarter of the sequence divided by the length of the protein
1004. The total number of glutamic acid residues in the third quarter of the sequence divided by the length of the protein
1005. The total number of glutamic acid residues in the fourth quarter of the sequence divided by the length of the protein
1006. The total number of glutamic acid residues in the first quarter of the sequence divided by the total number of glutamic acid blocks
1007. The total number of glutamic acid residues in the first quarter of the sequence divided by the total number of glutamic acid blocks
1008. The total number of glutamic acid residues in the first quarter of the sequence divided by the total number of glutamic acid blocks
1009. The total number of glutamic acid residues in the first quarter of the sequence divided by the total number of glutamic acid blocks
1010. The total number of glutamic acid residues in the first half of the protein
1011. The total number of glutamic acid residues in the three-quarter region of the protein
1012. The total number of glutamic acid residues from position 25% to 75% of the protein
1013. The total number of glutamic acid residues in the second half of the protein

1014. The total number of glutamic acid residues in the first half of the protein divided by the protein length

1015. The total number of glutamic acid residues in the three-quarter region of the protein divided by the protein length

1016. The total number of glutamic acid residues from position 25% to 75% of the protein divided by the protein length

1017. The total number of glutamic acid residues in the second half of the protein divided by the protein length

1018. The total number of glutamic acid residues in the first half of the protein divided by the total number of glutamic acid residues in the protein

1019. The total number of glutamic acid residues in the three-quarter region of the protein divided by the total number of glutamic acid residues in the protein

1020. The total number of glutamic acid residues from position 25% to 75% of the protein divided by the total number of glutamic acid residues in the protein

1021. The total number of glutamic acid residues in the second half of the protein divided by the total number of glutamic acid residues in the protein

1022. The total number of glutamic acid residue blocks in the protein

1023. The total number of glutamic acid residue blocks in the first quarter of the protein

1024. The total number of glutamic acid residue blocks in the second quarter of the protein

1025. The total number of glutamic acid residue blocks in the third quarter of the protein

1026. The total number of glutamic acid residue blocks in the fourth quarter of the protein

1027. The total number of glutamic acid residue blocks in the first quarter of the protein divided by the protein length

1028. The total number of glutamic acid residue blocks in the second quarter of the protein divided by the protein length

1029. The total number of glutamic acid residue blocks in the third quarter of the protein divided by the protein length

1030. The total number of glutamic acid residue blocks in the fourth quarter of the protein divided by the protein length

1031. The total number of glutamic acid residue blocks in the first quarter of the protein divided by the total number of glutamic acid blocks of the protein

1032. The total number of glutamic acid residue blocks in the second quarter of the protein divided by the total number of glutamic acid blocks of the protein

1033. The total number of glutamic acid residue blocks in the third quarter of the protein divided by the total number of glutamic acid blocks of the protein

1034. The total number of glutamic acid residue blocks in the fourth quarter of the protein divided by the total number of glutamic acid blocks of the protein

1035. The total number of glutamic acid residue blocks in the first half of the protein

1036. The total number of glutamic acid residue blocks in the three-quarter region of the protein

1037. The total number of glutamic acid residue blocks from position 25% to 75% of the protein

1038. The total number of glutamic acid residue blocks in the second half of the protein

1039. The total number of glutamic acid residue blocks in the first half of the protein divided by the protein length

1040. The total number of glutamic acid residue blocks in the three-quarter region of the protein divided by the protein length

1041. The total number of glutamic acid residue blocks from position 25% to 75% of the protein divided by the protein length

1042. The total number of glutamic acid residue blocks in the second half of the protein divided by the protein length

1043. The total number of glutamic acid residue blocks in the first half of the protein divided by the total number of glutamic acid blocks of the protein

1044. The total number of glutamic acid residue blocks in the three-quarter region of the protein divided by the total number of glutamic acid blocks of the protein

1045. The total number of glutamic acid residue blocks from position 25% to 75% of the protein divided by the total number of glutamic acid blocks of the protein

1046. The total number of glutamic acid residue blocks in the second half of the protein divided by the total number of glutamic acid blocks of the protein

1047. The length of the maximum glutamic acid block in the first quarter of the protein

1048. The median of the glutamic acid blocks in the first quarter of the protein

1049. The mean of the glutamic acid blocks in the first quarter of the protein

1050. The length of the maximum glutamic acid block in the second quarter of the protein

1051. The median of the glutamic acid blocks in the second quarter of the protein

1052. The mean of the glutamic acid blocks in the second quarter of the protein

1053. The length of the maximum glutamic acid block in the third quarter of the protein

1054. The median of the glutamic acid blocks in the third quarter of the protein

1055. The mean of the glutamic acid blocks in the third quarter of the protein

1056. The length of the maximum glutamic acid block in the fourth quarter of the protein

1057. The median of the glutamic acid blocks in the fourth quarter of the protein

1058. The mean of the glutamic acid blocks in the fourth quarter of the protein

1059. The length of the maximum glutamic acid block of the protein

1060. The median of the glutamic acid blocks of the protein

1061. The mean of the glutamic acid blocks of the protein

1062. The total number of phenylalanine residues in the sequence

1063. The total number of phenylalanine residues divided by the length of the protein

1064. The total number of phenylalanine residues in the first quarter of the sequence

1065. The total number of phenylalanine residues in the second quarter of the sequence

1066. The total number of phenylalanine residues in the third quarter of the sequence

1067. The total number of phenylalanine residues in the fourth quarter of the sequence

1068. The total number of phenylalanine residues in the first quarter of the sequence divided by the length of the protein

1069. The total number of phenylalanine residues in the second quarter of the sequence divided by the length of the protein

1070. The total number of phenylalanine residues in the third quarter of the sequence divided by the length of the protein

1071. The total number of phenylalanine residues in the fourth quarter of the sequence divided by the length of the protein

1072. The total number of phenylalanine residues in the first quarter of the sequence divided by the total number of phenylalanine blocks

1073. The total number of phenylalanine residues in the first quarter of the sequence divided by the total number of phenylalanine blocks

1074. The total number of phenylalanine residues in the first quarter of the sequence divided by the total number of phenylalanine blocks

1075. The total number of phenylalanine residues in the first quarter of the sequence divided by the total number of phenylalanine blocks

1076. The total number of phenylalanine residues in the first half of the protein

1077. The total number of phenylalanine residues in the three-quarter region of the protein

1078. The total number of phenylalanine residues from position 25% to 75% of the protein

1079. The total number of phenylalanine residues in the second half of the protein

1080. The total number of phenylalanine residues in the first half of the protein divided by the protein length

1081. The total number of phenylalanine residues in the three-quarter region of the protein divided by the protein length

1082. The total number of phenylalanine residues from position 25% to 75% of the protein divided by the protein length

1083. The total number of phenylalanine residues in the second half of the protein divided by the protein length

1084. The total number of phenylalanine residues in the first half of the protein divided by the total number of phenylalanine residues in the protein

1085. The total number of phenylalanine residues in the three-quarter region of the protein divided by the total number of phenylalanine residues in the protein

1086. The total number of phenylalanine residues from position 25% to 75% of the protein divided by the total number of phenylalanine residues in the protein

1087. The total number of phenylalanine residues in the second half of the protein divided by the total number of phenylalanine residues in the protein

1088. The total number of phenylalanine residue blocks in the protein

1089. The total number of phenylalanine residue blocks in the first quarter of the protein

1090. The total number of phenylalanine residue blocks in the second quarter of the protein

1091. The total number of phenylalanine residue blocks in the third quarter of the protein

1092. The total number of phenylalanine residue blocks in the fourth quarter of the protein

1093. The total number of phenylalanine residue blocks in the first quarter of the protein divided by the protein length

1094. The total number of phenylalanine residue blocks in the second quarter of the protein divided by the protein length

1095. The total number of phenylalanine residue blocks in the third quarter of the protein divided by the protein length

1096. The total number of phenylalanine residue blocks in the fourth quarter of the protein divided by the protein length

1097. The total number of phenylalanine residue blocks in the first quarter of the protein divided by the total number of phenylalanine blocks of the protein

1098. The total number of phenylalanine residue blocks in the second quarter of the protein divided by the total number of phenylalanine blocks of the protein

1099. The total number of phenylalanine residue blocks in the third quarter of the protein divided by the total number of phenylalanine blocks of the protein

1100. The total number of phenylalanine residue blocks in the fourth quarter of the protein divided by the total number of phenylalanine blocks of the protein

1101. The total number of phenylalanine residue blocks in the first half of the protein

1102. The total number of phenylalanine residue blocks in the three-quarter region of the protein

1103. The total number of phenylalanine residue blocks from position 25% to 75% of the protein

1104. The total number of phenylalanine residue blocks in the second half of the protein

1105. The total number of phenylalanine residue blocks in the first half of the protein divided by the protein length

1106. The total number of phenylalanine residue blocks in the three-quarter region of the protein divided by the protein length

1107. The total number of phenylalanine residue blocks from position 25% to 75% of the protein divided by the protein length

1108. The total number of phenylalanine residue blocks in the second half of the protein divided by the protein length

1109. The total number of phenylalanine residue blocks in the first half of the protein divided by the total number of phenylalanine blocks of the protein

1110. The total number of phenylalanine residue blocks in the three-quarter region of the protein divided by the total number of phenylalanine blocks of the protein

1111. The total number of phenylalanine residue blocks from position 25% to 75% of the protein divided by the total number of phenylalanine blocks of the protein

1112. The total number of phenylalanine residue blocks in the second half of the protein divided by the total number of phenylalanine blocks of the protein

1113. The length of the maximum phenylalanine block in the first quarter of the protein

1114. The median of the phenylalanine blocks in the first quarter of the protein

1115. The mean of the phenylalanine blocks in the first quarter of the protein

1116. The length of the maximum phenylalanine block in the second quarter of the protein

1117. The median of the phenylalanine blocks in the second quarter of the protein

1118. The mean of the phenylalanine blocks in the second quarter of the protein

1119. The length of the maximum phenylalanine block in the third quarter of the protein

1120. The median of the phenylalanine blocks in the third quarter of the protein

1121. The mean of the phenylalanine blocks in the third quarter of the protein

1122. The length of the maximum phenylalanine block in the fourth quarter of the protein

1123. The median of the phenylalanine blocks in the fourth quarter of the protein

1124. The mean of the phenylalanine blocks in the fourth quarter of the protein

1125. The length of the maximum phenylalanine block of the protein

1126. The median of the phenylalanine blocks of the protein

1127. The mean of the phenylalanine blocks of the protein

1128. The total number of glycine residues in the sequence

1129. The total number of glycine residues divided by the length of the protein

1130. The total number of glycine residues in the first quarter of the sequence

1131. The total number of glycine residues in the second quarter of the sequence

1132. The total number of glycine residues in the third quarter of the sequence

1133. The total number of glycine residues in the fourth quarter of the sequence

1134. The total number of glycine residues in the first quarter of the sequence divided by the length of the protein

1135. The total number of glycine residues in the second quarter of the sequence divided by the length of the protein

1136. The total number of glycine residues in the third quarter of the sequence divided by the length of the protein

1137. The total number of glycine residues in the fourth quarter of the sequence divided by the length of the protein

1138. The total number of glycine residues in the first quarter of the sequence divided by the total number of glycine blocks

1139. The total number of glycine residues in the first quarter of the sequence divided by the total number of glycine blocks

1140. The total number of glycine residues in the first quarter of the sequence divided by the total number of glycine blocks

1141. The total number of glycine residues in the first quarter of the sequence divided by the total number of glycine blocks

1142. The total number of glycine residues in the first half of the protein

1143. The total number of glycine residues in the three-quarter region of the protein

1144. The total number of glycine residues from position 25% to 75% of the protein

1145. The total number of glycine residues in the second half of the protein

1146. The total number of glycine residues in the first half of the protein divided by the protein length

1147. The total number of glycine residues in the three-quarter region of the protein divided by the protein length

1148. The total number of glycine residues from position 25% to 75% of the protein divided by the protein length

1149. The total number of glycine residues in the second half of the protein divided by the protein length

1150. The total number of glycine residues in the first half of the protein divided by the total number of glycine residues in the protein

1151. The total number of glycine residues in the three-quarter region of the protein divided by the total number of glycine residues in the protein

1152. The total number of glycine residues from position 25% to 75% of the protein divided by the total number of glycine residues in the protein

1153. The total number of glycine residues in the second half of the protein divided by the total number of glycine residues in the protein

1154. The total number of glycine residue blocks in the protein

1155. The total number of glycine residue blocks in the first quarter of the protein

1156. The total number of glycine residue blocks in the second quarter of the protein

1157. The total number of glycine residue blocks in the third quarter of the protein

1158. The total number of glycine residue blocks in the fourth quarter of the protein

1159. The total number of glycine residue blocks in the first quarter of the protein divided by the protein length

1160. The total number of glycine residue blocks in the second quarter of the protein divided by the protein length

1161. The total number of glycine residue blocks in the third quarter of the protein divided by the protein length

1162. The total number of glycine residue blocks in the fourth quarter of the protein divided by the protein length

1163. The total number of glycine residue blocks in the first quarter of the protein divided by the total number of glycine blocks of the protein

1164. The total number of glycine residue blocks in the second quarter of the protein divided by the total number of glycine blocks of the protein

1165. The total number of glycine residue blocks in the third quarter of the protein divided by the total number of glycine blocks of the protein

1166. The total number of glycine residue blocks in the fourth quarter of the protein divided by the total number of glycine blocks of the protein

1167. The total number of glycine residue blocks in the first half of the protein

1168. The total number of glycine residue blocks in the three-quarter region of the protein

1169. The total number of glycine residue blocks from position 25% to 75% of the protein

1170. The total number of glycine residue blocks in the second half of the protein

1171. The total number of glycine residue blocks in the first half of the protein divided by the protein length

1172. The total number of glycine residue blocks in the three-quarter region of the protein divided by the protein length

1173. The total number of glycine residue blocks from position 25% to 75% of the protein divided by the protein length

1174. The total number of glycine residue blocks in the second half of the protein divided by the protein length

1175. The total number of glycine residue blocks in the first half of the protein divided by the total number of glycine blocks of the protein

1176. The total number of glycine residue blocks in the three-quarter region of the protein divided by the total number of glycine blocks of the protein

1177. The total number of glycine residue blocks from position 25% to 75% of the protein divided by the total number of glycine blocks of the protein

1178. The total number of glycine residue blocks in the second half of the protein divided by the total number of glycine blocks of the protein

1179. The length of the maximum glycine block in the first quarter of the protein

1180. The median of the glycine blocks in the first quarter of the protein

1181. The mean of the glycine blocks in the first quarter of the protein

1182. The length of the maximum glycine block in the second quarter of the protein

1183. The median of the glycine blocks in the second quarter of the protein

1184. The mean of the glycine blocks in the second quarter of the protein

1185. The length of the maximum glycine block in the third quarter of the protein

1186. The median of the glycine blocks in the third quarter of the protein

1187. The mean of the glycine blocks in the third quarter of the protein

1188. The length of the maximum glycine block in the fourth quarter of the protein

1189. The median of the glycine blocks in the fourth quarter of the protein

1190. The mean of the glycine blocks in the fourth quarter of the protein

1191. The length of the maximum glycine block of the protein

1192. The median of the glycine blocks of the protein

1193. The mean of the glycine blocks of the protein

1194. The total number of histidine residues in the sequence

1195. The total number of histidine residues divided by the length of the protein

1196. The total number of histidine residues in the first quarter of the sequence

1197. The total number of histidine residues in the second quarter of the sequence

1198. The total number of histidine residues in the third quarter of the sequence

1199. The total number of histidine residues in the fourth quarter of the sequence

1200. The total number of histidine residues in the first quarter of the sequence divided by the length of the protein

1201. The total number of histidine residues in the second quarter of the sequence divided by the length of the protein

1202. The total number of histidine residues in the third quarter of the sequence divided by the length of the protein

1203. The total number of histidine residues in the fourth quarter of the sequence divided by the length of the protein

1204. The total number of histidine residues in the first quarter of the sequence divided by the total number of histidine blocks

1205. The total number of histidine residues in the first quarter of the sequence divided by the total number of histidine blocks

1206. The total number of histidine residues in the first quarter of the sequence divided by the total number of histidine blocks

1207. The total number of histidine residues in the first quarter of the sequence divided by the total number of histidine blocks

1208. The total number of histidine residues in the first half of the protein

1209. The total number of histidine residues in the three-quarter region of the protein

1210. The total number of histidine residues from position 25% to 75% of the protein

1211. The total number of histidine residues in the second half of the protein

1212. The total number of histidine residues in the first half of the protein divided by the protein length

1213. The total number of histidine residues in the three-quarter region of the protein divided by the protein length

1214. The total number of histidine residues from position 25% to 75% of the protein divided by the protein length

1215. The total number of histidine residues in the second half of the protein divided by the protein length

1216. The total number of histidine residues in the first half of the protein divided by the total number of histidine residues in the protein

1217. The total number of histidine residues in the three-quarter region of the protein divided by the total number of histidine residues in the protein

1218. The total number of histidine residues from position 25% to 75% of the protein divided by the total number of histidine residues in the protein

1219. The total number of histidine residues in the second half of the protein divided by the total number of histidine residues in the protein

1220. The total number of histidine residue blocks in the protein

1221. The total number of histidine residue blocks in the first quarter of the protein

1222. The total number of histidine residue blocks in the second quarter of the protein

1223. The total number of histidine residue blocks in the third quarter of the protein

1224. The total number of histidine residue blocks in the fourth quarter of the protein

1225. The total number of histidine residue blocks in the first quarter of the protein divided by the protein length

1226. The total number of histidine residue blocks in the second quarter of the protein divided by the protein length

1227. The total number of histidine residue blocks in the third quarter of the protein divided by the protein length

1228. The total number of histidine residue blocks in the fourth quarter of the protein divided by the protein length

1229. The total number of histidine residue blocks in the first quarter of the protein divided by the total number of histidine blocks of the protein

1230. The total number of histidine residue blocks in the second quarter of the protein divided by the total number of histidine blocks of the protein

1231. The total number of histidine residue blocks in the third quarter of the protein divided by the total number of histidine blocks of the protein

1232. The total number of histidine residue blocks in the fourth quarter of the protein divided by the total number of histidine blocks of the protein

1233. The total number of histidine residue blocks in the first half of the protein

1234. The total number of histidine residue blocks in the three-quarter region of the protein

1235. The total number of histidine residue blocks from position 25% to 75% of the protein

1236. The total number of histidine residue blocks in the second half of the protein

1237. The total number of histidine residue blocks in the first half of the protein divided by the protein length

1238. The total number of histidine residue blocks in the three-quarter region of the protein divided by the protein length

1239. The total number of histidine residue blocks from position 25% to 75% of the protein divided by the protein length

1240. The total number of histidine residue blocks in the second half of the protein divided by the protein length

1241. The total number of histidine residue blocks in the first half of the protein divided by the total number of histidine blocks of the protein

1242. The total number of histidine residue blocks in the three-quarter region of the protein divided by the total number of histidine blocks of the protein

1243. The total number of histidine residue blocks from position 25% to 75% of the protein divided by the total number of histidine blocks of the protein

1244. The total number of histidine residue blocks in the second half of the protein divided by the total number of histidine blocks of the protein

1245. The length of the maximum histidine block in the first quarter of the protein

1246. The median of the histidine blocks in the first quarter of the protein

1247. The mean of the histidine blocks in the first quarter of the protein

1248. The length of the maximum histidine block in the second quarter of the protein

1249. The median of the histidine blocks in the second quarter of the protein

1250. The mean of the histidine blocks in the second quarter of the protein

1251. The length of the maximum histidine block in the third quarter of the protein

1252. The median of the histidine blocks in the third quarter of the protein

1253. The mean of the histidine blocks in the third quarter of the protein

1254. The length of the maximum histidine block in the fourth quarter of the protein

1255. The median of the histidine blocks in the fourth quarter of the protein

1256. The mean of the histidine blocks in the fourth quarter of the protein

1257. The length of the maximum histidine block of the protein

1258. The median of the histidine blocks of the protein

1259. The mean of the histidine blocks of the protein

1260. The total number of lysine residues in the sequence
1261. The total number of lysine residues divided by the length of the protein
1262. The total number of lysine residues in the first quarter of the sequence
1263. The total number of lysine residues in the second quarter of the sequence
1264. The total number of lysine residues in the third quarter of the sequence
1265. The total number of lysine residues in the fourth quarter of the sequence
1266. The total number of lysine residues in the first quarter of the sequence divided by the length of the protein
1267. The total number of lysine residues in the second quarter of the sequence divided by the length of the protein
1268. The total number of lysine residues in the third quarter of the sequence divided by the length of the protein
1269. The total number of lysine residues in the fourth quarter of the sequence divided by the length of the protein
1270. The total number of lysine residues in the first quarter of the sequence divided by the total number of lysine blocks
1271. The total number of lysine residues in the first quarter of the sequence divided by the total number of lysine blocks
1272. The total number of lysine residues in the first quarter of the sequence divided by the total number of lysine blocks
1273. The total number of lysine residues in the first quarter of the sequence divided by the total number of lysine blocks
1274. The total number of lysine residues in the first half of the protein
1275. The total number of lysine residues in the three-quarter region of the protein
1276. The total number of lysine residues from position 25% to 75% of the protein
1277. The total number of lysine residues in the second half of the protein
1278. The total number of lysine residues in the first half of the protein divided by the protein length
1279. The total number of lysine residues in the three-quarter region of the protein divided by the protein length
1280. The total number of lysine residues from position 25% to 75% of the protein divided by the protein length
1281. The total number of lysine residues in the second half of the protein divided by the protein length

1282. The total number of lysine residues in the first half of the protein divided by the total number of lysine residues in the protein

1283. The total number of lysine residues in the three-quarter region of the protein divided by the total number of lysine residues in the protein

1284. The total number of lysine residues from position 25% to 75% of the protein divided by the total number of lysine residues in the protein

1285. The total number of lysine residues in the second half of the protein divided by the total number of lysine residues in the protein

1286. The total number of lysine residue blocks in the protein

1287. The total number of lysine residue blocks in the first quarter of the protein

1288. The total number of lysine residue blocks in the second quarter of the protein

1289. The total number of lysine residue blocks in the third quarter of the protein

1290. The total number of lysine residue blocks in the fourth quarter of the protein

1291. The total number of lysine residue blocks in the first quarter of the protein divided by the protein length

1292. The total number of lysine residue blocks in the second quarter of the protein divided by the protein length

1293. The total number of lysine residue blocks in the third quarter of the protein divided by the protein length

1294. The total number of lysine residue blocks in the fourth quarter of the protein divided by the protein length

1295. The total number of lysine residue blocks in the first quarter of the protein divided by the total number of lysine blocks of the protein

1296. The total number of lysine residue blocks in the second quarter of the protein divided by the total number of lysine blocks of the protein

1297. The total number of lysine residue blocks in the third quarter of the protein divided by the total number of lysine blocks of the protein

1298. The total number of lysine residue blocks in the fourth quarter of the protein divided by the total number of lysine blocks of the protein

1299. The total number of lysine residue blocks in the first half of the protein

1300. The total number of lysine residue blocks in the three-quarter region of the protein

1301. The total number of lysine residue blocks from position 25% to 75% of the protein

1302. The total number of lysine residue blocks in the second half of the protein

1303. The total number of lysine residue blocks in the first half of the protein divided by the protein length

1304. The total number of lysine residue blocks in the three-quarter region of the protein divided by the protein length

1305. The total number of lysine residue blocks from position 25% to 75% of the protein divided by the protein length

1306. The total number of lysine residue blocks in the second half of the protein divided by the protein length

1307. The total number of lysine residue blocks in the first half of the protein divided by the total number of lysine blocks of the protein

1308. The total number of lysine residue blocks in the three-quarter region of the protein divided by the total number of lysine blocks of the protein

1309. The total number of lysine residue blocks from position 25% to 75% of the protein divided by the total number of lysine blocks of the protein

1310. The total number of lysine residue blocks in the second half of the protein divided by the total number of lysine blocks of the protein

1311. The length of the maximum lysine block in the first quarter of the protein

1312. The median of the lysine blocks in the first quarter of the protein

1313. The mean of the lysine blocks in the first quarter of the protein

1314. The length of the maximum lysine block in the second quarter of the protein

1315. The median of the lysine blocks in the second quarter of the protein

1316. The mean of the lysine blocks in the second quarter of the protein

1317. The length of the maximum lysine block in the third quarter of the protein

1318. The median of the lysine blocks in the third quarter of the protein

1319. The mean of the lysine blocks in the third quarter of the protein

1320. The length of the maximum lysine block in the fourth quarter of the protein

1321. The median of the lysine blocks in the fourth quarter of the protein

1322. The mean of the lysine blocks in the fourth quarter of the protein

1323. The length of the maximum lysine block of the protein

1324. The median of the lysine blocks of the protein

1325. The mean of the lysine blocks of the protein

1326. The total number of isoleucine residues in the sequence

1327. The total number of isoleucine residues divided by the length of the protein

1328. The total number of isoleucine residues in the first quarter of the sequence

1329. The total number of isoleucine residues in the second quarter of the sequence

1330. The total number of isoleucine residues in the third quarter of the sequence

1331. The total number of isoleucine residues in the fourth quarter of the sequence

1332. The total number of isoleucine residues in the first quarter of the sequence divided by the length of the protein

1333. The total number of isoleucine residues in the second quarter of the sequence divided by the length of the protein

1334. The total number of isoleucine residues in the third quarter of the sequence divided by the length of the protein

1335. The total number of isoleucine residues in the fourth quarter of the sequence divided by the length of the protein

1336. The total number of isoleucine residues in the first quarter of the sequence divided by the total number of isoleucine blocks

1337. The total number of isoleucine residues in the first quarter of the sequence divided by the total number of isoleucine blocks

1338. The total number of isoleucine residues in the first quarter of the sequence divided by the total number of isoleucine blocks

1339. The total number of isoleucine residues in the first quarter of the sequence divided by the total number of isoleucine blocks

1340. The total number of isoleucine residues in the first half of the protein

1341. The total number of isoleucine residues in the three-quarter region of the protein

1342. The total number of isoleucine residues from position 25% to 75% of the protein

1343. The total number of isoleucine residues in the second half of the protein

1344. The total number of isoleucine residues in the first half of the protein divided by the protein length

1345. The total number of isoleucine residues in the three-quarter region of the protein divided by the protein length

1346. The total number of isoleucine residues from position 25% to 75% of the protein divided by the protein length

1347. The total number of isoleucine residues in the second half of the protein divided by the protein length

1348. The total number of isoleucine residues in the first half of the protein divided by the total number of isoleucine residues in the protein

1349. The total number of isoleucine residues in the three-quarter region of the protein divided by the total number of isoleucine residues in the protein

1350. The total number of isoleucine residues from position 25% to 75% of the protein divided by the total number of isoleucine residues in the protein

1351. The total number of isoleucine residues in the second half of the protein divided by the total number of isoleucine residues in the protein

1352. The total number of isoleucine residue blocks in the protein

1353. The total number of isoleucine residue blocks in the first quarter of the protein

1354. The total number of isoleucine residue blocks in the second quarter of the protein

1355. The total number of isoleucine residue blocks in the third quarter of the protein

1356. The total number of isoleucine residue blocks in the fourth quarter of the protein

1357. The total number of isoleucine residue blocks in the first quarter of the protein divided by the protein length

1358. The total number of isoleucine residue blocks in the second quarter of the protein divided by the protein length

1359. The total number of isoleucine residue blocks in the third quarter of the protein divided by the protein length

1360. The total number of isoleucine residue blocks in the fourth quarter of the protein divided by the protein length

1361. The total number of isoleucine residue blocks in the first quarter of the protein divided by the total number of isoleucine blocks of the protein

1362. The total number of isoleucine residue blocks in the second quarter of the protein divided by the total number of isoleucine blocks of the protein

1363. The total number of isoleucine residue blocks in the third quarter of the protein divided by the total number of isoleucine blocks of the protein

1364. The total number of isoleucine residue blocks in the fourth quarter of the protein divided by the total number of isoleucine blocks of the protein

1365. The total number of isoleucine residue blocks in the first half of the protein

1366. The total number of isoleucine residue blocks in the three-quarter region of the protein

1367. The total number of isoleucine residue blocks from position 25% to 75% of the protein

1368. The total number of isoleucine residue blocks in the second half of the protein

1369. The total number of isoleucine residue blocks in the first half of the protein divided by the protein length

1370. The total number of isoleucine residue blocks in the three-quarter region of the protein divided by the protein length

1371. The total number of isoleucine residue blocks from position 25% to 75% of the protein divided by the protein length

1372. The total number of isoleucine residue blocks in the second half of the protein divided by the protein length

1373. The total number of isoleucine residue blocks in the first half of the protein divided by the total number of isoleucine blocks of the protein

1374. The total number of isoleucine residue blocks in the three-quarter region of the protein divided by the total number of isoleucine blocks of the protein

1375. The total number of isoleucine residue blocks from position 25% to 75% of the protein divided by the total number of isoleucine blocks of the protein

1376. The total number of isoleucine residue blocks in the second half of the protein divided by the total number of isoleucine blocks of the protein

1377. The length of the maximum isoleucine block in the first quarter of the protein

1378. The median of the isoleucine blocks in the first quarter of the protein

1379. The mean of the isoleucine blocks in the first quarter of the protein

1380. The length of the maximum isoleucine block in the second quarter of the protein

1381. The median of the isoleucine blocks in the second quarter of the protein

1382. The mean of the isoleucine blocks in the second quarter of the protein

1383. The length of the maximum isoleucine block in the third quarter of the protein

1384. The median of the isoleucine blocks in the third quarter of the protein

1385. The mean of the isoleucine blocks in the third quarter of the protein

1386. The length of the maximum isoleucine block in the fourth quarter of the protein

1387. The median of the isoleucine blocks in the fourth quarter of the protein

1388. The mean of the isoleucine blocks in the fourth quarter of the protein

1389. The length of the maximum isoleucine block of the protein

1390. The median of the isoleucine blocks of the protein

1391. The mean of the isoleucine blocks of the protein

1392. The total number of leucine residues in the sequence

1393. The total number of leucine residues divided by the length of the protein

1394. The total number of leucine residues in the first quarter of the sequence

1395. The total number of leucine residues in the second quarter of the sequence

1396. The total number of leucine residues in the third quarter of the sequence

1397. The total number of leucine residues in the fourth quarter of the sequence

1398. The total number of leucine residues in the first quarter of the sequence divided by the length of the protein

1399. The total number of leucine residues in the second quarter of the sequence divided by the length of the protein

1400. The total number of leucine residues in the third quarter of the sequence divided by the length of the protein

1401. The total number of leucine residues in the fourth quarter of the sequence divided by the length of the protein

1402. The total number of leucine residues in the first quarter of the sequence divided by the total number of leucine blocks

1403. The total number of leucine residues in the first quarter of the sequence divided by the total number of leucine blocks

1404. The total number of leucine residues in the first quarter of the sequence divided by the total number of leucine blocks

1405. The total number of leucine residues in the first quarter of the sequence divided by the total number of leucine blocks

1406. The total number of leucine residues in the first half of the protein

1407. The total number of leucine residues in the three-quarter region of the protein

1408. The total number of leucine residues from position 25% to 75% of the protein

1409. The total number of leucine residues in the second half of the protein

1410. The total number of leucine residues in the first half of the protein divided by the protein length

1411. The total number of leucine residues in the three-quarter region of the protein divided by the protein length

1412. The total number of leucine residues from position 25% to 75% of the protein divided by the protein length

1413. The total number of leucine residues in the second half of the protein divided by the protein length

1414. The total number of leucine residues in the first half of the protein divided by the total number of leucine residues in the protein

1415. The total number of leucine residues in the three-quarter region of the protein divided by the total number of leucine residues in the protein

1416. The total number of leucine residues from position 25% to 75% of the protein divided by the total number of leucine residues in the protein

1417. The total number of leucine residues in the second half of the protein divided by the total number of leucine residues in the protein

1418. The total number of leucine residue blocks in the protein

1419. The total number of leucine residue blocks in the first quarter of the protein

1420. The total number of leucine residue blocks in the second quarter of the protein

1421. The total number of leucine residue blocks in the third quarter of the protein

1422. The total number of leucine residue blocks in the fourth quarter of the protein

1423. The total number of leucine residue blocks in the first quarter of the protein divided by the protein length

1424. The total number of leucine residue blocks in the second quarter of the protein divided by the protein length

1425. The total number of leucine residue blocks in the third quarter of the protein divided by the protein length

1426. The total number of leucine residue blocks in the fourth quarter of the protein divided by the protein length

1427. The total number of leucine residue blocks in the first quarter of the protein divided by the total number of leucine blocks of the protein

1428. The total number of leucine residue blocks in the second quarter of the protein divided by the total number of leucine blocks of the protein

1429. The total number of leucine residue blocks in the third quarter of the protein divided by the total number of leucine blocks of the protein

1430. The total number of leucine residue blocks in the fourth quarter of the protein divided by the total number of leucine blocks of the protein

1431. The total number of leucine residue blocks in the first half of the protein

1432. The total number of leucine residue blocks in the three-quarter region of the protein

1433. The total number of leucine residue blocks from position 25% to 75% of the protein

1434. The total number of leucine residue blocks in the second half of the protein

1435. The total number of leucine residue blocks in the first half of the protein divided by the protein length

1436. The total number of leucine residue blocks in the three-quarter region of the protein divided by the protein length

1437. The total number of leucine residue blocks from position 25% to 75% of the protein divided by the protein length

1438. The total number of leucine residue blocks in the second half of the protein divided by the protein length

1439. The total number of leucine residue blocks in the first half of the protein divided by the total number of leucine blocks of the protein

1440. The total number of leucine residue blocks in the three-quarter region of the protein divided by the total number of leucine blocks of the protein

1441. The total number of leucine residue blocks from position 25% to 75% of the protein divided by the total number of leucine blocks of the protein

1442. The total number of leucine residue blocks in the second half of the protein divided by the total number of leucine blocks of the protein

1443. The length of the maximum leucine block in the first quarter of the protein

1444. The median of the leucine blocks in the first quarter of the protein

1445. The mean of the leucine blocks in the first quarter of the protein

1446. The length of the maximum leucine block in the second quarter of the protein

1447. The median of the leucine blocks in the second quarter of the protein

1448. The mean of the leucine blocks in the second quarter of the protein

1449. The length of the maximum leucine block in the third quarter of the protein

1450. The median of the leucine blocks in the third quarter of the protein

1451. The mean of the leucine blocks in the third quarter of the protein

1452. The length of the maximum leucine block in the fourth quarter of the protein

1453. The median of the leucine blocks in the fourth quarter of the protein

1454. The mean of the leucine blocks in the fourth quarter of the protein

1455. The length of the maximum leucine block of the protein

1456. The median of the leucine blocks of the protein

1457. The mean of the leucine blocks of the protein

1458. The total number of methionine residues in the sequence

1459. The total number of methionine residues divided by the length of the protein

1460. The total number of methionine residues in the first quarter of the sequence

1461. The total number of methionine residues in the second quarter of the sequence

1462. The total number of methionine residues in the third quarter of the sequence

1463. The total number of methionine residues in the fourth quarter of the sequence

1464. The total number of methionine residues in the first quarter of the sequence divided by the length of the protein

1465. The total number of methionine residues in the second quarter of the sequence divided by the length of the protein

1466. The total number of methionine residues in the third quarter of the sequence divided by the length of the protein

1467. The total number of methionine residues in the fourth quarter of the sequence divided by the length of the protein

1468. The total number of methionine residues in the first quarter of the sequence divided by the total number of methionine blocks

1469. The total number of methionine residues in the first quarter of the sequence divided by the total number of methionine blocks

1470. The total number of methionine residues in the first quarter of the sequence divided by the total number of methionine blocks

1471. The total number of methionine residues in the first quarter of the sequence divided by the total number of methionine blocks

1472. The total number of methionine residues in the first half of the protein

1473. The total number of methionine residues in the three-quarter region of the protein

1474. The total number of methionine residues from position 25% to 75% of the protein

1475. The total number of methionine residues in the second half of the protein

1476. The total number of methionine residues in the first half of the protein divided by the protein length

1477. The total number of methionine residues in the three-quarter region of the protein divided by the protein length

1478. The total number of methionine residues from position 25% to 75% of the protein divided by the protein length

1479. The total number of methionine residues in the second half of the protein divided by the protein length

1480. The total number of methionine residues in the first half of the protein divided by the total number of methionine residues in the protein

1481. The total number of methionine residues in the three-quarter region of the protein divided by the total number of methionine residues in the protein

1482. The total number of methionine residues from position 25% to 75% of the protein divided by the total number of methionine residues in the protein

1483. The total number of methionine residues in the second half of the protein divided by the total number of methionine residues in the protein

1484. The total number of methionine residue blocks in the protein

1485. The total number of methionine residue blocks in the first quarter of the protein

1486. The total number of methionine residue blocks in the second quarter of the protein

1487. The total number of methionine residue blocks in the third quarter of the protein

1488. The total number of methionine residue blocks in the fourth quarter of the protein

1489. The total number of methionine residue blocks in the first quarter of the protein divided by the protein length

1490. The total number of methionine residue blocks in the second quarter of the protein divided by the protein length

1491. The total number of methionine residue blocks in the third quarter of the protein divided by the protein length

1492. The total number of methionine residue blocks in the fourth quarter of the protein divided by the protein length

1493. The total number of methionine residue blocks in the first quarter of the protein divided by the total number of methionine blocks of the protein

1494. The total number of methionine residue blocks in the second quarter of the protein divided by the total number of methionine blocks of the protein

1495. The total number of methionine residue blocks in the third quarter of the protein divided by the total number of methionine blocks of the protein

1496. The total number of methionine residue blocks in the fourth quarter of the protein divided by the total number of methionine blocks of the protein

1497. The total number of methionine residue blocks in the first half of the protein

1498. The total number of methionine residue blocks in the three-quarter region of the protein

1499. The total number of methionine residue blocks from position 25% to 75% of the protein

1500. The total number of methionine residue blocks in the second half of the protein

1501. The total number of methionine residue blocks in the first half of the protein divided by the protein length

1502. The total number of methionine residue blocks in the three-quarter region of the protein divided by the protein length

1503. The total number of methionine residue blocks from position 25% to 75% of the protein divided by the protein length

1504. The total number of methionine residue blocks in the second half of the protein divided by the protein length

1505. The total number of methionine residue blocks in the first half of the protein divided by the total number of methionine blocks of the protein

1506. The total number of methionine residue blocks in the three-quarter region of the protein divided by the total number of methionine blocks of the protein

1507. The total number of methionine residue blocks from position 25% to 75% of the protein divided by the total number of methionine blocks of the protein

1508. The total number of methionine residue blocks in the second half of the protein divided by the total number of methionine blocks of the protein

1509. The length of the maximum methionine block in the first quarter of the protein

1510. The median of the methionine blocks in the first quarter of the protein

1511. The mean of the methionine blocks in the first quarter of the protein

1512. The length of the maximum methionine block in the second quarter of the protein

1513. The median of the methionine blocks in the second quarter of the protein

1514. The mean of the methionine blocks in the second quarter of the protein

1515. The length of the maximum methionine block in the third quarter of the protein

1516. The median of the methionine blocks in the third quarter of the protein

1517. The mean of the methionine blocks in the third quarter of the protein

1518. The length of the maximum methionine block in the fourth quarter of the protein

1519. The median of the methionine blocks in the fourth quarter of the protein

1520. The mean of the methionine blocks in the fourth quarter of the protein

1521. The length of the maximum methionine block of the protein

1522. The median of the methionine blocks of the protein

1523. The mean of the methionine blocks of the protein

1524. The total number of asparagine residues in the sequence

1525. The total number of asparagine residues divided by the length of the protein

1526. The total number of asparagine residues in the first quarter of the sequence

1527. The total number of asparagine residues in the second quarter of the sequence

1528. The total number of asparagine residues in the third quarter of the sequence

1529. The total number of asparagine residues in the fourth quarter of the sequence

1530. The total number of asparagine residues in the first quarter of the sequence divided by the length of the protein

1531. The total number of asparagine residues in the second quarter of the sequence divided by the length of the protein

1532. The total number of asparagine residues in the third quarter of the sequence divided by the length of the protein

1533. The total number of asparagine residues in the fourth quarter of the sequence divided by the length of the protein

1534. The total number of asparagine residues in the first quarter of the sequence divided by the total number of asparagine blocks

1535. The total number of asparagine residues in the first quarter of the sequence divided by the total number of asparagine blocks

1536. The total number of asparagine residues in the first quarter of the sequence divided by the total number of asparagine blocks

1537. The total number of asparagine residues in the first quarter of the sequence divided by the total number of asparagine blocks

1538. The total number of asparagine residues in the first half of the protein

1539. The total number of asparagine residues in the three-quarter region of the protein

1540. The total number of asparagine residues from position 25% to 75% of the protein

1541. The total number of asparagine residues in the second half of the protein

1542. The total number of asparagine residues in the first half of the protein divided by the protein length

1543. The total number of asparagine residues in the three-quarter region of the protein divided by the protein length

1544. The total number of asparagine residues from position 25% to 75% of the protein divided by the protein length

1545. The total number of asparagine residues in the second half of the protein divided by the protein length

1546. The total number of asparagine residues in the first half of the protein divided by the total number of asparagine residues in the protein

1547. The total number of asparagine residues in the three-quarter region of the protein divided by the total number of asparagine residues in the protein

1548. The total number of asparagine residues from position 25% to 75% of the protein divided by the total number of asparagine residues in the protein

1549. The total number of asparagine residues in the second half of the protein divided by the total number of asparagine residues in the protein

1550. The total number of asparagine residue blocks in the protein

1551. The total number of asparagine residue blocks in the first quarter of the protein

1552. The total number of asparagine residue blocks in the second quarter of the protein

1553. The total number of asparagine residue blocks in the third quarter of the protein

1554. The total number of asparagine residue blocks in the fourth quarter of the protein

1555. The total number of asparagine residue blocks in the first quarter of the protein divided by the protein length

1556. The total number of asparagine residue blocks in the second quarter of the protein divided by the protein length

1557. The total number of asparagine residue blocks in the third quarter of the protein divided by the protein length

1558. The total number of asparagine residue blocks in the fourth quarter of the protein divided by the protein length

1559. The total number of asparagine residue blocks in the first quarter of the protein divided by the total number of asparagine blocks of the protein

1560. The total number of asparagine residue blocks in the second quarter of the protein divided by the total number of asparagine blocks of the protein

1561. The total number of asparagine residue blocks in the third quarter of the protein divided by the total number of asparagine blocks of the protein

1562. The total number of asparagine residue blocks in the fourth quarter of the protein divided by the total number of asparagine blocks of the protein

1563. The total number of asparagine residue blocks in the first half of the protein

1564. The total number of asparagine residue blocks in the three-quarter region of the protein

1565. The total number of asparagine residue blocks from position 25% to 75% of the protein

1566. The total number of asparagine residue blocks in the second half of the protein

1567. The total number of asparagine residue blocks in the first half of the protein divided by the protein length

1568. The total number of asparagine residue blocks in the three-quarter region of the protein divided by the protein length

1569. The total number of asparagine residue blocks from position 25% to 75% of the protein divided by the protein length

1570. The total number of asparagine residue blocks in the second half of the protein divided by the protein length

1571. The total number of asparagine residue blocks in the first half of the protein divided by the total number of asparagine blocks of the protein

1572. The total number of asparagine residue blocks in the three-quarter region of the protein divided by the total number of asparagine blocks of the protein

1573. The total number of asparagine residue blocks from position 25% to 75% of the protein divided by the total number of asparagine blocks of the protein

1574. The total number of asparagine residue blocks in the second half of the protein divided by the total number of asparagine blocks of the protein

1575. The length of the maximum asparagine block in the first quarter of the protein

1576. The median of the asparagine blocks in the first quarter of the protein

1577. The mean of the asparagine blocks in the first quarter of the protein

1578. The length of the maximum asparagine block in the second quarter of the protein

1579. The median of the asparagine blocks in the second quarter of the protein

1580. The mean of the asparagine blocks in the second quarter of the protein

1581. The length of the maximum asparagine block in the third quarter of the protein

1582. The median of the asparagine blocks in the third quarter of the protein

1583. The mean of the asparagine blocks in the third quarter of the protein

1584. The length of the maximum asparagine block in the fourth quarter of the protein

1585. The median of the asparagine blocks in the fourth quarter of the protein

1586. The mean of the asparagine blocks in the fourth quarter of the protein

1587. The length of the maximum asparagine block of the protein

1588. The median of the asparagine blocks of the protein

1589. The mean of the asparagine blocks of the protein

1590. The total number of proline residues in the sequence

1591. The total number of proline residues divided by the length of the protein

1592. The total number of proline residues in the first quarter of the sequence

1593. The total number of proline residues in the second quarter of the sequence

1594. The total number of proline residues in the third quarter of the sequence

1595. The total number of proline residues in the fourth quarter of the sequence

1596. The total number of proline residues in the first quarter of the sequence divided by the length of the protein

1597. The total number of proline residues in the second quarter of the sequence divided by the length of the protein

1598. The total number of proline residues in the third quarter of the sequence divided by the length of the protein

1599. The total number of proline residues in the fourth quarter of the sequence divided by the length of the protein

1600. The total number of proline residues in the first quarter of the sequence divided by the total number of proline blocks

1601. The total number of proline residues in the first quarter of the sequence divided by the total number of proline blocks

1602. The total number of proline residues in the first quarter of the sequence divided by the total number of proline blocks

1603. The total number of proline residues in the first quarter of the sequence divided by the total number of proline blocks

1604. The total number of proline residues in the first half of the protein

1605. The total number of proline residues in the three-quarter region of the protein

1606. The total number of proline residues from position 25% to 75% of the protein

1607. The total number of proline residues in the second half of the protein

1608. The total number of proline residues in the first half of the protein divided by the protein length

1609. The total number of proline residues in the three-quarter region of the protein divided by the protein length

1610. The total number of proline residues from position 25% to 75% of the protein divided by the protein length

1611. The total number of proline residues in the second half of the protein divided by the protein length

1612. The total number of proline residues in the first half of the protein divided by the total number of proline residues in the protein

1613. The total number of proline residues in the three-quarter region of the protein divided by the total number of proline residues in the protein

1614. The total number of proline residues from position 25% to 75% of the protein divided by the total number of proline residues in the protein

1615. The total number of proline residues in the second half of the protein divided by the total number of proline residues in the protein

1616. The total number of proline residue blocks in the protein

1617. The total number of proline residue blocks in the first quarter of the protein

1618. The total number of proline residue blocks in the second quarter of the protein

1619. The total number of proline residue blocks in the third quarter of the protein

1620. The total number of proline residue blocks in the fourth quarter of the protein

1621. The total number of proline residue blocks in the first quarter of the protein divided by the protein length

1622. The total number of proline residue blocks in the second quarter of the protein divided by the protein length

1623. The total number of proline residue blocks in the third quarter of the protein divided by the protein length

1624. The total number of proline residue blocks in the fourth quarter of the protein divided by the protein length

1625. The total number of proline residue blocks in the first quarter of the protein divided by the total number of proline blocks of the protein

1626. The total number of proline residue blocks in the second quarter of the protein divided by the total number of proline blocks of the protein

1627. The total number of proline residue blocks in the third quarter of the protein divided by the total number of proline blocks of the protein

1628. The total number of proline residue blocks in the fourth quarter of the protein divided by the total number of proline blocks of the protein

1629. The total number of proline residue blocks in the first half of the protein

1630. The total number of proline residue blocks in the three-quarter region of the protein

1631. The total number of proline residue blocks from position 25% to 75% of the protein

1632. The total number of proline residue blocks in the second half of the protein

1633. The total number of proline residue blocks in the first half of the protein divided by the protein length

1634. The total number of proline residue blocks in the three-quarter region of the protein divided by the protein length

1635. The total number of proline residue blocks from position 25% to 75% of the protein divided by the protein length

1636. The total number of proline residue blocks in the second half of the protein divided by the protein length

1637. The total number of proline residue blocks in the first half of the protein divided by the total number of proline blocks of the protein

1638. The total number of proline residue blocks in the three-quarter region of the protein divided by the total number of proline blocks of the protein

1639. The total number of proline residue blocks from position 25% to 75% of the protein divided by the total number of proline blocks of the protein

1640. The total number of proline residue blocks in the second half of the protein divided by the total number of proline blocks of the protein

1641. The length of the maximum proline block in the first quarter of the protein

1642. The median of the proline blocks in the first quarter of the protein

1643. The mean of the proline blocks in the first quarter of the protein

1644. The length of the maximum proline block in the second quarter of the protein

1645. The median of the proline blocks in the second quarter of the protein

1646. The mean of the proline blocks in the second quarter of the protein

1647. The length of the maximum proline block in the third quarter of the protein

1648. The median of the proline blocks in the third quarter of the protein

1649. The mean of the proline blocks in the third quarter of the protein

1650. The length of the maximum proline block in the fourth quarter of the protein

1651. The median of the proline blocks in the fourth quarter of the protein

1652. The mean of the proline blocks in the fourth quarter of the protein

1653. The length of the maximum proline block of the protein

1654. The median of the proline blocks of the protein

1655. The mean of the proline blocks of the protein

1656. The total number of glutamine residues in the sequence

1657. The total number of glutamine residues divided by the length of the protein

1658. The total number of glutamine residues in the first quarter of the sequence

1659. The total number of glutamine residues in the second quarter of the sequence

1660. The total number of glutamine residues in the third quarter of the sequence

1661. The total number of glutamine residues in the fourth quarter of the sequence

1662. The total number of glutamine residues in the first quarter of the sequence divided by the length of the protein

1663. The total number of glutamine residues in the second quarter of the sequence divided by the length of the protein

1664. The total number of glutamine residues in the third quarter of the sequence divided by the length of the protein

1665. The total number of glutamine residues in the fourth quarter of the sequence divided by the length of the protein

1666. The total number of glutamine residues in the first quarter of the sequence divided by the total number of glutamine blocks

1667. The total number of glutamine residues in the first quarter of the sequence divided by the total number of glutamine blocks

1668. The total number of glutamine residues in the first quarter of the sequence divided by the total number of glutamine blocks

1669. The total number of glutamine residues in the first quarter of the sequence divided by the total number of glutamine blocks

1670. The total number of glutamine residues in the first half of the protein

1671. The total number of glutamine residues in the three-quarter region of the protein

1672. The total number of glutamine residues from position 25% to 75% of the protein

1673. The total number of glutamine residues in the second half of the protein

1674. The total number of glutamine residues in the first half of the protein divided by the protein length

1675. The total number of glutamine residues in the three-quarter region of the protein divided by the protein length

1676. The total number of glutamine residues from position 25% to 75% of the protein divided by the protein length

1677. The total number of glutamine residues in the second half of the protein divided by the protein length

1678. The total number of glutamine residues in the first half of the protein divided by the total number of glutamine residues in the protein

1679. The total number of glutamine residues in the three-quarter region of the protein divided by the total number of glutamine residues in the protein

1680. The total number of glutamine residues from position 25% to 75% of the protein divided by the total number of glutamine residues in the protein

1681. The total number of glutamine residues in the second half of the protein divided by the total number of glutamine residues in the protein

1682. The total number of glutamine residue blocks in the protein

1683. The total number of glutamine residue blocks in the first quarter of the protein

1684. The total number of glutamine residue blocks in the second quarter of the protein

1685. The total number of glutamine residue blocks in the third quarter of the protein

1686. The total number of glutamine residue blocks in the fourth quarter of the protein

1687. The total number of glutamine residue blocks in the first quarter of the protein divided by the protein length

1688. The total number of glutamine residue blocks in the second quarter of the protein divided by the protein length

1689. The total number of glutamine residue blocks in the third quarter of the protein divided by the protein length

1690. The total number of glutamine residue blocks in the fourth quarter of the protein divided by the protein length

1691. The total number of glutamine residue blocks in the first quarter of the protein divided by the total number of glutamine blocks of the protein

1692. The total number of glutamine residue blocks in the second quarter of the protein divided by the total number of glutamine blocks of the protein

1693. The total number of glutamine residue blocks in the third quarter of the protein divided by the total number of glutamine blocks of the protein

1694. The total number of glutamine residue blocks in the fourth quarter of the protein divided by the total number of glutamine blocks of the protein

1695. The total number of glutamine residue blocks in the first half of the protein

1696. The total number of glutamine residue blocks in the three-quarter region of the protein

1697. The total number of glutamine residue blocks from position 25% to 75% of the protein

1698. The total number of glutamine residue blocks in the second half of the protein

1699. The total number of glutamine residue blocks in the first half of the protein divided by the protein length

1700. The total number of glutamine residue blocks in the three-quarter region of the protein divided by the protein length

1701. The total number of glutamine residue blocks from position 25% to 75% of the protein divided by the protein length

1702. The total number of glutamine residue blocks in the second half of the protein divided by the protein length

1703. The total number of glutamine residue blocks in the first half of the protein divided by the total number of glutamine blocks of the protein

1704. The total number of glutamine residue blocks in the three-quarter region of the protein divided by the total number of glutamine blocks of the protein

1705. The total number of glutamine residue blocks from position 25% to 75% of the protein divided by the total number of glutamine blocks of the protein

1706. The total number of glutamine residue blocks in the second half of the protein divided by the total number of glutamine blocks of the protein

1707. The length of the maximum glutamine block in the first quarter of the protein

1708. The median of the glutamine blocks in the first quarter of the protein

1709. The mean of the glutamine blocks in the first quarter of the protein

1710. The length of the maximum glutamine block in the second quarter of the protein

1711. The median of the glutamine blocks in the second quarter of the protein

1712. The mean of the glutamine blocks in the second quarter of the protein

1713. The length of the maximum glutamine block in the third quarter of the protein

1714. The median of the glutamine blocks in the third quarter of the protein

1715. The mean of the glutamine blocks in the third quarter of the protein

1716. The length of the maximum glutamine block in the fourth quarter of the protein

1717. The median of the glutamine blocks in the fourth quarter of the protein

1718. The mean of the glutamine blocks in the fourth quarter of the protein

1719. The length of the maximum glutamine block of the protein

1720. The median of the glutamine blocks of the protein

1721. The mean of the glutamine blocks of the protein

1722. The total number of arginine residues in the sequence

1723. The total number of arginine residues divided by the length of the protein

1724. The total number of arginine residues in the first quarter of the sequence

1725. The total number of arginine residues in the second quarter of the sequence

1726. The total number of arginine residues in the third quarter of the sequence

1727. The total number of arginine residues in the fourth quarter of the sequence

1728. The total number of arginine residues in the first quarter of the sequence divided by the length of the protein

1729. The total number of arginine residues in the second quarter of the sequence divided by the length of the protein

1730. The total number of arginine residues in the third quarter of the sequence divided by the length of the protein

1731. The total number of arginine residues in the fourth quarter of the sequence divided by the length of the protein

1732. The total number of arginine residues in the first quarter of the sequence divided by the total number of arginine blocks

1733. The total number of arginine residues in the first quarter of the sequence divided by the total number of arginine blocks

1734. The total number of arginine residues in the first quarter of the sequence divided by the total number of arginine blocks

1735. The total number of arginine residues in the first quarter of the sequence divided by the total number of arginine blocks

1736. The total number of arginine residues in the first half of the protein

1737. The total number of arginine residues in the three-quarter region of the protein

1738. The total number of arginine residues from position 25% to 75% of the protein

1739. The total number of arginine residues in the second half of the protein

1740. The total number of arginine residues in the first half of the protein divided by the protein length

1741. The total number of arginine residues in the three-quarter region of the protein divided by the protein length

1742. The total number of arginine residues from position 25% to 75% of the protein divided by the protein length

1743. The total number of arginine residues in the second half of the protein divided by the protein length

1744. The total number of arginine residues in the first half of the protein divided by the total number of arginine residues in the protein

1745. The total number of arginine residues in the three-quarter region of the protein divided by the total number of arginine residues in the protein

1746. The total number of arginine residues from position 25% to 75% of the protein divided by the total number of arginine residues in the protein

1747. The total number of arginine residues in the second half of the protein divided by the total number of arginine residues in the protein

1748. The total number of arginine residue blocks in the protein

1749. The total number of arginine residue blocks in the first quarter of the protein

1750. The total number of arginine residue blocks in the second quarter of the protein

1751. The total number of arginine residue blocks in the third quarter of the protein

1752. The total number of arginine residue blocks in the fourth quarter of the protein

1753. The total number of arginine residue blocks in the first quarter of the protein divided by the protein length

1754. The total number of arginine residue blocks in the second quarter of the protein divided by the protein length

1755. The total number of arginine residue blocks in the third quarter of the protein divided by the protein length

1756. The total number of arginine residue blocks in the fourth quarter of the protein divided by the protein length

1757. The total number of arginine residue blocks in the first quarter of the protein divided by the total number of arginine blocks of the protein

1758. The total number of arginine residue blocks in the second quarter of the protein divided by the total number of arginine blocks of the protein

1759. The total number of arginine residue blocks in the third quarter of the protein divided by the total number of arginine blocks of the protein

1760. The total number of arginine residue blocks in the fourth quarter of the protein divided by the total number of arginine blocks of the protein

1761. The total number of arginine residue blocks in the first half of the protein

1762. The total number of arginine residue blocks in the three-quarter region of the protein

1763. The total number of arginine residue blocks from position 25% to 75% of the protein

1764. The total number of arginine residue blocks in the second half of the protein

1765. The total number of arginine residue blocks in the first half of the protein divided by the protein length

1766. The total number of arginine residue blocks in the three-quarter region of the protein divided by the protein length

1767. The total number of arginine residue blocks from position 25% to 75% of the protein divided by the protein length

1768. The total number of arginine residue blocks in the second half of the protein divided by the protein length

1769. The total number of arginine residue blocks in the first half of the protein divided by the total number of arginine blocks of the protein

1770. The total number of arginine residue blocks in the three-quarter region of the protein divided by the total number of arginine blocks of the protein

1771. The total number of arginine residue blocks from position 25% to 75% of the protein divided by the total number of arginine blocks of the protein

1772. The total number of arginine residue blocks in the second half of the protein divided by the total number of arginine blocks of the protein

1773. The length of the maximum arginine block in the first quarter of the protein

1774. The median of the arginine blocks in the first quarter of the protein

1775. The mean of the arginine blocks in the first quarter of the protein

1776. The length of the maximum arginine block in the second quarter of the protein

1777. The median of the arginine blocks in the second quarter of the protein

1778. The mean of the arginine blocks in the second quarter of the protein

1779. The length of the maximum arginine block in the third quarter of the protein

1780. The median of the arginine blocks in the third quarter of the protein

1781. The mean of the arginine blocks in the third quarter of the protein

1782. The length of the maximum arginine block in the fourth quarter of the protein

1783. The median of the arginine blocks in the fourth quarter of the protein

1784. The mean of the arginine blocks in the fourth quarter of the protein

1785. The length of the maximum arginine block of the protein

1786. The median of the arginine blocks of the protein

1787. The mean of the arginine blocks of the protein

1788. The total number of serine residues in the sequence

1789. The total number of serine residues divided by the length of the protein

1790. The total number of serine residues in the first quarter of the sequence

1791. The total number of serine residues in the second quarter of the sequence

1792. The total number of serine residues in the third quarter of the sequence

1793. The total number of serine residues in the fourth quarter of the sequence

1794. The total number of serine residues in the first quarter of the sequence divided by the length of the protein

1795. The total number of serine residues in the second quarter of the sequence divided by the length of the protein

1796. The total number of serine residues in the third quarter of the sequence divided by the length of the protein

1797. The total number of serine residues in the fourth quarter of the sequence divided by the length of the protein

1798. The total number of serine residues in the first quarter of the sequence divided by the total number of serine blocks

1799. The total number of serine residues in the first quarter of the sequence divided by the total number of serine blocks

1800. The total number of serine residues in the first quarter of the sequence divided by the total number of serine blocks

1801. The total number of serine residues in the first quarter of the sequence divided by the total number of serine blocks

1802. The total number of serine residues in the first half of the protein

1803. The total number of serine residues in the three-quarter region of the protein

1804. The total number of serine residues from position 25% to 75% of the protein

1805. The total number of serine residues in the second half of the protein

1806. The total number of serine residues in the first half of the protein divided by the protein length

1807. The total number of serine residues in the three-quarter region of the protein divided by the protein length

1808. The total number of serine residues from position 25% to 75% of the protein divided by the protein length

1809. The total number of serine residues in the second half of the protein divided by the protein length

1810. The total number of serine residues in the first half of the protein divided by the total number of serine residues in the protein

1811. The total number of serine residues in the three-quarter region of the protein divided by the total number of serine residues in the protein

1812. The total number of serine residues from position 25% to 75% of the protein divided by the total number of serine residues in the protein

1813. The total number of serine residues in the second half of the protein divided by the total number of serine residues in the protein

1814. The total number of serine residue blocks in the protein

1815. The total number of serine residue blocks in the first quarter of the protein

1816. The total number of serine residue blocks in the second quarter of the protein

1817. The total number of serine residue blocks in the third quarter of the protein

1818. The total number of serine residue blocks in the fourth quarter of the protein

1819. The total number of serine residue blocks in the first quarter of the protein divided by the protein length

1820. The total number of serine residue blocks in the second quarter of the protein divided by the protein length

1821. The total number of serine residue blocks in the third quarter of the protein divided by the protein length

1822. The total number of serine residue blocks in the fourth quarter of the protein divided by the protein length

1823. The total number of serine residue blocks in the first quarter of the protein divided by the total number of serine blocks of the protein

1824. The total number of serine residue blocks in the second quarter of the protein divided by the total number of serine blocks of the protein

1825. The total number of serine residue blocks in the third quarter of the protein divided by the total number of serine blocks of the protein

1826. The total number of serine residue blocks in the fourth quarter of the protein divided by the total number of serine blocks of the protein

1827. The total number of serine residue blocks in the first half of the protein

1828. The total number of serine residue blocks in the three-quarter region of the protein

1829. The total number of serine residue blocks from position 25% to 75% of the protein

1830. The total number of serine residue blocks in the second half of the protein

1831. The total number of serine residue blocks in the first half of the protein divided by the protein length

1832. The total number of serine residue blocks in the three-quarter region of the protein divided by the protein length

1833. The total number of serine residue blocks from position 25% to 75% of the protein divided by the protein length

1834. The total number of serine residue blocks in the second half of the protein divided by the protein length

1835. The total number of serine residue blocks in the first half of the protein divided by the total number of serine blocks of the protein

1836. The total number of serine residue blocks in the three-quarter region of the protein divided by the total number of serine blocks of the protein

1837. The total number of serine residue blocks from position 25% to 75% of the protein divided by the total number of serine blocks of the protein

1838. The total number of serine residue blocks in the second half of the protein divided by the total number of serine blocks of the protein

1839. The length of the maximum serine block in the first quarter of the protein

1840. The median of the serine blocks in the first quarter of the protein

1841. The mean of the serine blocks in the first quarter of the protein

1842. The length of the maximum serine block in the second quarter of the protein

1843. The median of the serine blocks in the second quarter of the protein

1844. The mean of the serine blocks in the second quarter of the protein

1845. The length of the maximum serine block in the third quarter of the protein

1846. The median of the serine blocks in the third quarter of the protein

1847. The mean of the serine blocks in the third quarter of the protein

1848. The length of the maximum serine block in the fourth quarter of the protein

1849. The median of the serine blocks in the fourth quarter of the protein

1850. The mean of the serine blocks in the fourth quarter of the protein

1851. The length of the maximum serine block of the protein

1852. The median of the serine blocks of the protein

1853. The mean of the serine blocks of the protein

1854. The total number of threonine residues in the sequence

1855. The total number of threonine residues divided by the length of the protein

1856. The total number of threonine residues in the first quarter of the sequence

1857. The total number of threonine residues in the second quarter of the sequence

1858. The total number of threonine residues in the third quarter of the sequence

1859. The total number of threonine residues in the fourth quarter of the sequence

1860. The total number of threonine residues in the first quarter of the sequence divided by the length of the protein

1861. The total number of threonine residues in the second quarter of the sequence divided by the length of the protein

1862. The total number of threonine residues in the third quarter of the sequence divided by the length of the protein

1863. The total number of threonine residues in the fourth quarter of the sequence divided by the length of the protein

1864. The total number of threonine residues in the first quarter of the sequence divided by the total number of threonine blocks

1865. The total number of threonine residues in the first quarter of the sequence divided by the total number of threonine blocks

1866. The total number of threonine residues in the first quarter of the sequence divided by the total number of threonine blocks

1867. The total number of threonine residues in the first quarter of the sequence divided by the total number of threonine blocks

1868. The total number of threonine residues in the first half of the protein

1869. The total number of threonine residues in the three-quarter region of the protein

1870. The total number of threonine residues from position 25% to 75% of the protein

1871. The total number of threonine residues in the second half of the protein

1872. The total number of threonine residues in the first half of the protein divided by the protein length

1873. The total number of threonine residues in the three-quarter region of the protein divided by the protein length

1874. The total number of threonine residues from position 25% to 75% of the protein divided by the protein length

1875. The total number of threonine residues in the second half of the protein divided by the protein length

1876. The total number of threonine residues in the first half of the protein divided by the total number of threonine residues in the protein

1877. The total number of threonine residues in the three-quarter region of the protein divided by the total number of threonine residues in the protein

1878. The total number of threonine residues from position 25% to 75% of the protein divided by the total number of threonine residues in the protein

1879. The total number of threonine residues in the second half of the protein divided by the total number of threonine residues in the protein

1880. The total number of threonine residue blocks in the protein

1881. The total number of threonine residue blocks in the first quarter of the protein

1882. The total number of threonine residue blocks in the second quarter of the protein

1883. The total number of threonine residue blocks in the third quarter of the protein

1884. The total number of threonine residue blocks in the fourth quarter of the protein

1885. The total number of threonine residue blocks in the first quarter of the protein divided by the protein length

1886. The total number of threonine residue blocks in the second quarter of the protein divided by the protein length

1887. The total number of threonine residue blocks in the third quarter of the protein divided by the protein length

1888. The total number of threonine residue blocks in the fourth quarter of the protein divided by the protein length

1889. The total number of threonine residue blocks in the first quarter of the protein divided by the total number of threonine blocks of the protein

1890. The total number of threonine residue blocks in the second quarter of the protein divided by the total number of threonine blocks of the protein

1891. The total number of threonine residue blocks in the third quarter of the protein divided by the total number of threonine blocks of the protein

1892. The total number of threonine residue blocks in the fourth quarter of the protein divided by the total number of threonine blocks of the protein

1893. The total number of threonine residue blocks in the first half of the protein

1894. The total number of threonine residue blocks in the three-quarter region of the protein

1895. The total number of threonine residue blocks from position 25% to 75% of the protein

1896. The total number of threonine residue blocks in the second half of the protein

1897. The total number of threonine residue blocks in the first half of the protein divided by the protein length

1898. The total number of threonine residue blocks in the three-quarter region of the protein divided by the protein length

1899. The total number of threonine residue blocks from position 25% to 75% of the protein divided by the protein length

1900. The total number of threonine residue blocks in the second half of the protein divided by the protein length

1901. The total number of threonine residue blocks in the first half of the protein divided by the total number of threonine blocks of the protein

1902. The total number of threonine residue blocks in the three-quarter region of the protein divided by the total number of threonine blocks of the protein

1903. The total number of threonine residue blocks from position 25% to 75% of the protein divided by the total number of threonine blocks of the protein

1904. The total number of threonine residue blocks in the second half of the protein divided by the total number of threonine blocks of the protein

1905. The length of the maximum threonine block in the first quarter of the protein

1906. The median of the threonine blocks in the first quarter of the protein

1907. The mean of the threonine blocks in the first quarter of the protein

1908. The length of the maximum threonine block in the second quarter of the protein

1909. The median of the threonine blocks in the second quarter of the protein

1910. The mean of the threonine blocks in the second quarter of the protein

1911. The length of the maximum threonine block in the third quarter of the protein

1912. The median of the threonine blocks in the third quarter of the protein

1913. The mean of the threonine blocks in the third quarter of the protein

1914. The length of the maximum threonine block in the fourth quarter of the protein

1915. The median of the threonine blocks in the fourth quarter of the protein

1916. The mean of the threonine blocks in the fourth quarter of the protein

1917. The length of the maximum threonine block of the protein

1918. The median of the threonine blocks of the protein

1919. The mean of the threonine blocks of the protein

1920. The total number of valine residues in the sequence

1921. The total number of valine residues divided by the length of the protein

1922. The total number of valine residues in the first quarter of the sequence

1923. The total number of valine residues in the second quarter of the sequence

1924. The total number of valine residues in the third quarter of the sequence

1925. The total number of valine residues in the fourth quarter of the sequence

1926. The total number of valine residues in the first quarter of the sequence divided by the length of the protein

1927. The total number of valine residues in the second quarter of the sequence divided by the length of the protein

1928. The total number of valine residues in the third quarter of the sequence divided by the length of the protein

1929. The total number of valine residues in the fourth quarter of the sequence divided by the length of the protein

1930. The total number of valine residues in the first quarter of the sequence divided by the total number of valine blocks

1931. The total number of valine residues in the first quarter of the sequence divided by the total number of valine blocks

1932. The total number of valine residues in the first quarter of the sequence divided by the total number of valine blocks

1933. The total number of valine residues in the first quarter of the sequence divided by the total number of valine blocks

1934. The total number of valine residues in the first half of the protein

1935. The total number of valine residues in the three-quarter region of the protein

1936. The total number of valine residues from position 25% to 75% of the protein

1937. The total number of valine residues in the second half of the protein

1938. The total number of valine residues in the first half of the protein divided by the protein length

1939. The total number of valine residues in the three-quarter region of the protein divided by the protein length

1940. The total number of valine residues from position 25% to 75% of the protein divided by the protein length

1941. The total number of valine residues in the second half of the protein divided by the protein length

1942. The total number of valine residues in the first half of the protein divided by the total number of valine residues in the protein

1943. The total number of valine residues in the three-quarter region of the protein divided by the total number of valine residues in the protein

1944. The total number of valine residues from position 25% to 75% of the protein divided by the total number of valine residues in the protein

1945. The total number of valine residues in the second half of the protein divided by the total number of valine residues in the protein

1946. The total number of valine residue blocks in the protein

1947. The total number of valine residue blocks in the first quarter of the protein

1948. The total number of valine residue blocks in the second quarter of the protein

1949. The total number of valine residue blocks in the third quarter of the protein

1950. The total number of valine residue blocks in the fourth quarter of the protein

1951. The total number of valine residue blocks in the first quarter of the protein divided by the protein length

1952. The total number of valine residue blocks in the second quarter of the protein divided by the protein length

1953. The total number of valine residue blocks in the third quarter of the protein divided by the protein length

1954. The total number of valine residue blocks in the fourth quarter of the protein divided by the protein length

1955. The total number of valine residue blocks in the first quarter of the protein divided by the total number of valine blocks of the protein

1956. The total number of valine residue blocks in the second quarter of the protein divided by the total number of valine blocks of the protein

1957. The total number of valine residue blocks in the third quarter of the protein divided by the total number of valine blocks of the protein

1958. The total number of valine residue blocks in the fourth quarter of the protein divided by the total number of valine blocks of the protein

1959. The total number of valine residue blocks in the first half of the protein

1960. The total number of valine residue blocks in the three-quarter region of the protein

1961. The total number of valine residue blocks from position 25% to 75% of the protein

1962. The total number of valine residue blocks in the second half of the protein

1963. The total number of valine residue blocks in the first half of the protein divided by the protein length

1964. The total number of valine residue blocks in the three-quarter region of the protein divided by the protein length

1965. The total number of valine residue blocks from position 25% to 75% of the protein divided by the protein length

1966. The total number of valine residue blocks in the second half of the protein divided by the protein length

1967. The total number of valine residue blocks in the first half of the protein divided by the total number of valine blocks of the protein

1968. The total number of valine residue blocks in the three-quarter region of the protein divided by the total number of valine blocks of the protein

1969. The total number of valine residue blocks from position 25% to 75% of the protein divided by the total number of valine blocks of the protein

1970. The total number of valine residue blocks in the second half of the protein divided by the total number of valine blocks of the protein

1971. The length of the maximum valine block in the first quarter of the protein

1972. The median of the valine blocks in the first quarter of the protein

1973. The mean of the valine blocks in the first quarter of the protein

1974. The length of the maximum valine block in the second quarter of the protein

1975. The median of the valine blocks in the second quarter of the protein

1976. The mean of the valine blocks in the second quarter of the protein

1977. The length of the maximum valine block in the third quarter of the protein

1978. The median of the valine blocks in the third quarter of the protein

1979. The mean of the valine blocks in the third quarter of the protein

1980. The length of the maximum valine block in the fourth quarter of the protein

1981. The median of the valine blocks in the fourth quarter of the protein

1982. The mean of the valine blocks in the fourth quarter of the protein

1983. The length of the maximum valine block of the protein

1984. The median of the valine blocks of the protein

1985. The mean of the valine blocks of the protein

1986. The total number of tryptophan residues in the sequence

1987. The total number of tryptophan residues divided by the length of the protein

1988. The total number of tryptophan residues in the first quarter of the sequence

1989. The total number of tryptophan residues in the second quarter of the sequence

1990. The total number of tryptophan residues in the third quarter of the sequence

1991. The total number of tryptophan residues in the fourth quarter of the sequence

1992. The total number of tryptophan residues in the first quarter of the sequence divided by the length of the protein

1993. The total number of tryptophan residues in the second quarter of the sequence divided by the length of the protein

1994. The total number of tryptophan residues in the third quarter of the sequence divided by the length of the protein

1995. The total number of tryptophan residues in the fourth quarter of the sequence divided by the length of the protein

1996. The total number of tryptophan residues in the first quarter of the sequence divided by the total number of tryptophan blocks

1997. The total number of tryptophan residues in the first quarter of the sequence divided by the total number of tryptophan blocks

1998. The total number of tryptophan residues in the first quarter of the sequence divided by the total number of tryptophan blocks

1999. The total number of tryptophan residues in the first quarter of the sequence divided by the total number of tryptophan blocks

2000. The total number of tryptophan residues in the first half of the protein

2001. The total number of tryptophan residues in the three-quarter region of the protein

2002. The total number of tryptophan residues from position 25% to 75% of the protein

2003. The total number of tryptophan residues in the second half of the protein

2004. The total number of tryptophan residues in the first half of the protein divided by the protein length

2005. The total number of tryptophan residues in the three-quarter region of the protein divided by the protein length

2006. The total number of tryptophan residues from position 25% to 75% of the protein divided by the protein length

2007. The total number of tryptophan residues in the second half of the protein divided by the protein length

2008. The total number of tryptophan residues in the first half of the protein divided by the total number of tryptophan residues in the protein

2009. The total number of tryptophan residues in the three-quarter region of the protein divided by the total number of tryptophan residues in the protein

2010. The total number of tryptophan residues from position 25% to 75% of the protein divided by the total number of tryptophan residues in the protein

2011. The total number of tryptophan residues in the second half of the protein divided by the total number of tryptophan residues in the protein

2012. The total number of tryptophan residue blocks in the protein

2013. The total number of tryptophan residue blocks in the first quarter of the protein

2014. The total number of tryptophan residue blocks in the second quarter of the protein

2015. The total number of tryptophan residue blocks in the third quarter of the protein

2016. The total number of tryptophan residue blocks in the fourth quarter of the protein

2017. The total number of tryptophan residue blocks in the first quarter of the protein divided by the protein length

2018. The total number of tryptophan residue blocks in the second quarter of the protein divided by the protein length

2019. The total number of tryptophan residue blocks in the third quarter of the protein divided by the protein length

2020. The total number of tryptophan residue blocks in the fourth quarter of the protein divided by the protein length

2021. The total number of tryptophan residue blocks in the first quarter of the protein divided by the total number of tryptophan blocks of the protein

2022. The total number of tryptophan residue blocks in the second quarter of the protein divided by the total number of tryptophan blocks of the protein

2023. The total number of tryptophan residue blocks in the third quarter of the protein divided by the total number of tryptophan blocks of the protein

2024. The total number of tryptophan residue blocks in the fourth quarter of the protein divided by the total number of tryptophan blocks of the protein

2025. The total number of tryptophan residue blocks in the first half of the protein

2026. The total number of tryptophan residue blocks in the three-quarter region of the protein

2027. The total number of tryptophan residue blocks from position 25% to 75% of the protein

2028. The total number of tryptophan residue blocks in the second half of the protein

2029. The total number of tryptophan residue blocks in the first half of the protein divided by the protein length

2030. The total number of tryptophan residue blocks in the three-quarter region of the protein divided by the protein length

2031. The total number of tryptophan residue blocks from position 25% to 75% of the protein divided by the protein length

2032. The total number of tryptophan residue blocks in the second half of the protein divided by the protein length

2033. The total number of tryptophan residue blocks in the first half of the protein divided by the total number of tryptophan blocks of the protein

2034. The total number of tryptophan residue blocks in the three-quarter region of the protein divided by the total number of tryptophan blocks of the protein

2035. The total number of tryptophan residue blocks from position 25% to 75% of the protein divided by the total number of tryptophan blocks of the protein

2036. The total number of tryptophan residue blocks in the second half of the protein divided by the total number of tryptophan blocks of the protein

2037. The length of the maximum tryptophan block in the first quarter of the protein

2038. The median of the tryptophan blocks in the first quarter of the protein

2039. The mean of the tryptophan blocks in the first quarter of the protein

2040. The length of the maximum tryptophan block in the second quarter of the protein

2041. The median of the tryptophan blocks in the second quarter of the protein

2042. The mean of the tryptophan blocks in the second quarter of the protein

2043. The length of the maximum tryptophan block in the third quarter of the protein

2044. The median of the tryptophan blocks in the third quarter of the protein

2045. The mean of the tryptophan blocks in the third quarter of the protein

2046. The length of the maximum tryptophan block in the fourth quarter of the protein

2047. The median of the tryptophan blocks in the fourth quarter of the protein

2048. The mean of the tryptophan blocks in the fourth quarter of the protein

2049. The length of the maximum tryptophan block of the protein
2050. The median of the tryptophan blocks of the protein
2051. The mean of the tryptophan blocks of the protein
2052. The total number of tyrosine residues in the sequence
2053. The total number of tyrosine residues divided by the length of the protein
2054. The total number of tyrosine residues in the first quarter of the sequence
2055. The total number of tyrosine residues in the second quarter of the sequence
2056. The total number of tyrosine residues in the third quarter of the sequence
2057. The total number of tyrosine residues in the fourth quarter of the sequence
2058. The total number of tyrosine residues in the first quarter of the sequence divided by the length of the protein
2059. The total number of tyrosine residues in the second quarter of the sequence divided by the length of the protein
2060. The total number of tyrosine residues in the third quarter of the sequence divided by the length of the protein
2061. The total number of tyrosine residues in the fourth quarter of the sequence divided by the length of the protein
2062. The total number of tyrosine residues in the first quarter of the sequence divided by the total number of tyrosine blocks
2063. The total number of tyrosine residues in the first quarter of the sequence divided by the total number of tyrosine blocks
2064. The total number of tyrosine residues in the first quarter of the sequence divided by the total number of tyrosine blocks
2065. The total number of tyrosine residues in the first quarter of the sequence divided by the total number of tyrosine blocks
2066. The total number of tyrosine residues in the first half of the protein
2067. The total number of tyrosine residues in the three-quarter region of the protein
2068. The total number of tyrosine residues from position 25% to 75% of the protein
2069. The total number of tyrosine residues in the second half of the protein
2070. The total number of tyrosine residues in the first half of the protein divided by the protein length
2071. The total number of tyrosine residues in the three-quarter region of the protein divided by the protein length

2072. The total number of tyrosine residues from position 25% to 75% of the protein divided by the protein length

2073. The total number of tyrosine residues in the second half of the protein divided by the protein length

2074. The total number of tyrosine residues in the first half of the protein divided by the total number of tyrosine residues in the protein

2075. The total number of tyrosine residues in the three-quarter region of the protein divided by the total number of tyrosine residues in the protein

2076. The total number of tyrosine residues from position 25% to 75% of the protein divided by the total number of tyrosine residues in the protein

2077. The total number of tyrosine residues in the second half of the protein divided by the total number of tyrosine residues in the protein

2078. The total number of tyrosine residue blocks in the protein

2079. The total number of tyrosine residue blocks in the first quarter of the protein

2080. The total number of tyrosine residue blocks in the second quarter of the protein

2081. The total number of tyrosine residue blocks in the third quarter of the protein

2082. The total number of tyrosine residue blocks in the fourth quarter of the protein

2083. The total number of tyrosine residue blocks in the first quarter of the protein divided by the protein length

2084. The total number of tyrosine residue blocks in the second quarter of the protein divided by the protein length

2085. The total number of tyrosine residue blocks in the third quarter of the protein divided by the protein length

2086. The total number of tyrosine residue blocks in the fourth quarter of the protein divided by the protein length

2087. The total number of tyrosine residue blocks in the first quarter of the protein divided by the total number of tyrosine blocks of the protein

2088. The total number of tyrosine residue blocks in the second quarter of the protein divided by the total number of tyrosine blocks of the protein

2089. The total number of tyrosine residue blocks in the third quarter of the protein divided by the total number of tyrosine blocks of the protein

2090. The total number of tyrosine residue blocks in the fourth quarter of the protein divided by the total number of tyrosine blocks of the protein

2091. The total number of tyrosine residue blocks in the first half of the protein

2092. The total number of tyrosine residue blocks in the three-quarter region of the protein

2093. The total number of tyrosine residue blocks from position 25% to 75% of the protein

2094. The total number of tyrosine residue blocks in the second half of the protein

2095. The total number of tyrosine residue blocks in the first half of the protein divided by the protein length

2096. The total number of tyrosine residue blocks in the three-quarter region of the protein divided by the protein length

2097. The total number of tyrosine residue blocks from position 25% to 75% of the protein divided by the protein length

2098. The total number of tyrosine residue blocks in the second half of the protein divided by the protein length

2099. The total number of tyrosine residue blocks in the first half of the protein divided by the total number of tyrosine blocks of the protein

2100. The total number of tyrosine residue blocks in the three-quarter region of the protein divided by the total number of tyrosine blocks of the protein

2101. The total number of tyrosine residue blocks from position 25% to 75% of the protein divided by the total number of tyrosine blocks of the protein

2102. The total number of tyrosine residue blocks in the second half of the protein divided by the total number of tyrosine blocks of the protein

2103. The length of the maximum tyrosine block in the first quarter of the protein

2104. The median of the tyrosine blocks in the first quarter of the protein

2105. The mean of the tyrosine blocks in the first quarter of the protein

2106. The length of the maximum tyrosine block in the second quarter of the protein

2107. The median of the tyrosine blocks in the second quarter of the protein

2108. The mean of the tyrosine blocks in the second quarter of the protein

2109. The length of the maximum tyrosine block in the third quarter of the protein

2110. The median of the tyrosine blocks in the third quarter of the protein

2111. The mean of the tyrosine blocks in the third quarter of the protein

2112. The length of the maximum tyrosine block in the fourth quarter of the protein

2113. The median of the tyrosine blocks in the fourth quarter of the protein

2114. The mean of the tyrosine blocks in the fourth quarter of the protein

2115. The length of the maximum tyrosine block of the protein

2116. The median of the tyrosine blocks of the protein

2117. The mean of the tyrosine blocks of the protein

2118. The total number of alpha residues in the sequence

2119. The total number of alpha residues divided by the length of the protein

2120. The total number of alpha residues in the first quarter of the sequence

2121. The total number of alpha residues in the second quarter of the sequence

2122. The total number of alpha residues in the third quarter of the sequence

2123. The total number of alpha residues in the fourth quarter of the sequence

2124. The total number of alpha residues in the first quarter of the sequence divided by the length of the protein

2125. The total number of alpha residues in the second quarter of the sequence divided by the length of the protein

2126. The total number of alpha residues in the third quarter of the sequence divided by the length of the protein

2127. The total number of alpha residues in the fourth quarter of the sequence divided by the length of the protein

2128. The total number of alpha residues in the first quarter of the sequence divided by the total number of alpha blocks

2129. The total number of alpha residues in the first quarter of the sequence divided by the total number of alpha blocks

2130. The total number of alpha residues in the first quarter of the sequence divided by the total number of alpha blocks

2131. The total number of alpha residues in the first quarter of the sequence divided by the total number of alpha blocks

2132. The total number of alpha residues in the first half of the protein

2133. The total number of alpha residues in the three-quarter region of the protein

2134. The total number of alpha residues from position 25% to 75% of the protein

2135. The total number of alpha residues in the second half of the protein

2136. The total number of alpha residues in the first half of the protein divided by the protein length

2137. The total number of alpha residues in the three-quarter region of the protein divided by the protein length

2138. The total number of alpha residues from position 25% to 75% of the protein divided by the protein length

2139. The total number of alpha residues in the second half of the protein divided by the protein length

2140. The total number of alpha residues in the first half of the protein divided by the total number of alpha residues in the protein

2141. The total number of alpha residues in the three-quarter region of the protein divided by the total number of alpha residues in the protein

2142. The total number of alpha residues from position 25% to 75% of the protein divided by the total number of alpha residues in the protein

2143. The total number of alpha residues in the second half of the protein divided by the total number of alpha residues in the protein

2144. The total number of alpha residue blocks in the protein

2145. The total number of alpha residue blocks in the first quarter of the protein

2146. The total number of alpha residue blocks in the second quarter of the protein

2147. The total number of alpha residue blocks in the third quarter of the protein

2148. The total number of alpha residue blocks in the fourth quarter of the protein

2149. The total number of alpha residue blocks in the first quarter of the protein divided by the protein length

2150. The total number of alpha residue blocks in the second quarter of the protein divided by the protein length

2151. The total number of alpha residue blocks in the third quarter of the protein divided by the protein length

2152. The total number of alpha residue blocks in the fourth quarter of the protein divided by the protein length

2153. The total number of alpha residue blocks in the first quarter of the protein divided by the total number of alpha blocks of the protein

2154. The total number of alpha residue blocks in the second quarter of the protein divided by the total number of alpha blocks of the protein

2155. The total number of alpha residue blocks in the third quarter of the protein divided by the total number of alpha blocks of the protein

2156. The total number of alpha residue blocks in the fourth quarter of the protein divided by the total number of alpha blocks of the protein

2157. The total number of alpha residue blocks in the first half of the protein

2158. The total number of alpha residue blocks in the three-quarter region of the protein

2159. The total number of alpha residue blocks from position 25% to 75% of the protein

2160. The total number of alpha residue blocks in the second half of the protein

2161. The total number of alpha residue blocks in the first half of the protein divided by the protein length

2162. The total number of alpha residue blocks in the three-quarter region of the protein divided by the protein length

2163. The total number of alpha residue blocks from position 25% to 75% of the protein divided by the protein length

2164. The total number of alpha residue blocks in the second half of the protein divided by the protein length

2165. The total number of alpha residue blocks in the first half of the protein divided by the total number of alpha blocks of the protein

2166. The total number of alpha residue blocks in the three-quarter region of the protein divided by the total number of alpha blocks of the protein

2167. The total number of alpha residue blocks from position 25% to 75% of the protein divided by the total number of alpha blocks of the protein

2168. The total number of alpha residue blocks in the second half of the protein divided by the total number of alpha blocks of the protein

2169. The length of the maximum alpha block in the first quarter of the protein

2170. The median of the alpha blocks in the first quarter of the protein

2171. The mean of the alpha blocks in the first quarter of the protein

2172. The length of the maximum alpha block in the second quarter of the protein

2173. The median of the alpha blocks in the second quarter of the protein

2174. The mean of the alpha blocks in the second quarter of the protein

2175. The length of the maximum alpha block in the third quarter of the protein

2176. The median of the alpha blocks in the third quarter of the protein

2177. The mean of the alpha blocks in the third quarter of the protein

2178. The length of the maximum alpha block in the fourth quarter of the protein

2179. The median of the alpha blocks in the fourth quarter of the protein

2180. The mean of the alpha blocks in the fourth quarter of the protein

2181. The length of the maximum alpha block of the protein

2182. The median of the alpha blocks of the protein

2183. The mean of the alpha blocks of the protein

2184. The total number of beta residues in the sequence

2185. The total number of beta residues divided by the length of the protein

2186. The total number of beta residues in the first quarter of the sequence

2187. The total number of beta residues in the second quarter of the sequence

2188. The total number of beta residues in the third quarter of the sequence

2189. The total number of beta residues in the fourth quarter of the sequence

2190. The total number of beta residues in the first quarter of the sequence divided by the length of the protein

2191. The total number of beta residues in the second quarter of the sequence divided by the length of the protein

2192. The total number of beta residues in the third quarter of the sequence divided by the length of the protein

2193. The total number of beta residues in the fourth quarter of the sequence divided by the length of the protein

2194. The total number of beta residues in the first quarter of the sequence divided by the total number of beta blocks

2195. The total number of beta residues in the first quarter of the sequence divided by the total number of beta blocks

2196. The total number of beta residues in the first quarter of the sequence divided by the total number of beta blocks

2197. The total number of beta residues in the first quarter of the sequence divided by the total number of beta blocks

2198. The total number of beta residues in the first half of the protein

2199. The total number of beta residues in the three-quarter region of the protein

2200. The total number of beta residues from position 25% to 75% of the protein

2201. The total number of beta residues in the second half of the protein

2202. The total number of beta residues in the first half of the protein divided by the protein length

2203. The total number of beta residues in the three-quarter region of the protein divided by the protein length

2204. The total number of beta residues from position 25% to 75% of the protein divided by the protein length

2205. The total number of beta residues in the second half of the protein divided by the protein length

2206. The total number of beta residues in the first half of the protein divided by the total number of beta residues in the protein

2207. The total number of beta residues in the three-quarter region of the protein divided by the total number of beta residues in the protein

2208. The total number of beta residues from position 25% to 75% of the protein divided by the total number of beta residues in the protein

2209. The total number of beta residues in the second half of the protein divided by the total number of beta residues in the protein

2210. The total number of beta residue blocks in the protein

2211. The total number of beta residue blocks in the first quarter of the protein

2212. The total number of beta residue blocks in the second quarter of the protein

2213. The total number of beta residue blocks in the third quarter of the protein

2214. The total number of beta residue blocks in the fourth quarter of the protein

2215. The total number of beta residue blocks in the first quarter of the protein divided by the protein length

2216. The total number of beta residue blocks in the second quarter of the protein divided by the protein length

2217. The total number of beta residue blocks in the third quarter of the protein divided by the protein length

2218. The total number of beta residue blocks in the fourth quarter of the protein divided by the protein length

2219. The total number of beta residue blocks in the first quarter of the protein divided by the total number of beta blocks of the protein

2220. The total number of beta residue blocks in the second quarter of the protein divided by the total number of beta blocks of the protein

2221. The total number of beta residue blocks in the third quarter of the protein divided by the total number of beta blocks of the protein

2222. The total number of beta residue blocks in the fourth quarter of the protein divided by the total number of beta blocks of the protein

2223. The total number of beta residue blocks in the first half of the protein

2224. The total number of beta residue blocks in the three-quarter region of the protein

2225. The total number of beta residue blocks from position 25% to 75% of the protein

2226. The total number of beta residue blocks in the second half of the protein

2227. The total number of beta residue blocks in the first half of the protein divided by the protein length

2228. The total number of beta residue blocks in the three-quarter region of the protein divided by the protein length

2229. The total number of beta residue blocks from position 25% to 75% of the protein divided by the protein length

2230. The total number of beta residue blocks in the second half of the protein divided by the protein length

2231. The total number of beta residue blocks in the first half of the protein divided by the total number of beta blocks of the protein

2232. The total number of beta residue blocks in the three-quarter region of the protein divided by the total number of beta blocks of the protein

2233. The total number of beta residue blocks from position 25% to 75% of the protein divided by the total number of beta blocks of the protein

2234. The total number of beta residue blocks in the second half of the protein divided by the total number of beta blocks of the protein

2235. The length of the maximum beta block in the first quarter of the protein

2236. The median of the beta blocks in the first quarter of the protein

2237. The mean of the beta blocks in the first quarter of the protein

2238. The length of the maximum beta block in the second quarter of the protein

2239. The median of the beta blocks in the second quarter of the protein

2240. The mean of the beta blocks in the second quarter of the protein

2241. The length of the maximum beta block in the third quarter of the protein

2242. The median of the beta blocks in the third quarter of the protein

2243. The mean of the beta blocks in the third quarter of the protein

2244. The length of the maximum beta block in the fourth quarter of the protein

2245. The median of the beta blocks in the fourth quarter of the protein

2246. The mean of the beta blocks in the fourth quarter of the protein

2247. The length of the maximum beta block of the protein

2248. The median of the beta blocks of the protein

2249. The mean of the beta blocks of the protein

2250. The total number of coil residues in the sequence

2251. The total number of coil residues divided by the length of the protein

2252. The total number of coil residues in the first quarter of the sequence

2253. The total number of coil residues in the second quarter of the sequence

2254. The total number of coil residues in the third quarter of the sequence

2255. The total number of coil residues in the fourth quarter of the sequence

2256. The total number of coil residues in the first quarter of the sequence divided by the length of the protein

2257. The total number of coil residues in the second quarter of the sequence divided by the length of the protein

2258. The total number of coil residues in the third quarter of the sequence divided by the length of the protein

2259. The total number of coil residues in the fourth quarter of the sequence divided by the length of the protein

2260. The total number of coil residues in the first quarter of the sequence divided by the total number of coil blocks

2261. The total number of coil residues in the first quarter of the sequence divided by the total number of coil blocks

2262. The total number of coil residues in the first quarter of the sequence divided by the total number of coil blocks

2263. The total number of coil residues in the first quarter of the sequence divided by the total number of coil blocks

2264. The total number of coil residues in the first half of the protein

2265. The total number of coil residues in the three-quarter region of the protein

2266. The total number of coil residues from position 25% to 75% of the protein

2267. The total number of coil residues in the second half of the protein

2268. The total number of coil residues in the first half of the protein divided by the protein length

2269. The total number of coil residues in the three-quarter region of the protein divided by the protein length

2270. The total number of coil residues from position 25% to 75% of the protein divided by the protein length

2271. The total number of coil residues in the second half of the protein divided by the protein length

2272. The total number of coil residues in the first half of the protein divided by the total number of coil residues in the protein

2273. The total number of coil residues in the three-quarter region of the protein divided by the total number of coil residues in the protein

2274. The total number of coil residues from position 25% to 75% of the protein divided by the total number of coil residues in the protein

2275. The total number of coil residues in the second half of the protein divided by the total number of coil residues in the protein

2276. The total number of coil residue blocks in the protein

2277. The total number of coil residue blocks in the first quarter of the protein

2278. The total number of coil residue blocks in the second quarter of the protein

2279. The total number of coil residue blocks in the third quarter of the protein

2280. The total number of coil residue blocks in the fourth quarter of the protein

2281. The total number of coil residue blocks in the first quarter of the protein divided by the protein length

2282. The total number of coil residue blocks in the second quarter of the protein divided by the protein length

2283. The total number of coil residue blocks in the third quarter of the protein divided by the protein length

2284. The total number of coil residue blocks in the fourth quarter of the protein divided by the protein length

2285. The total number of coil residue blocks in the first quarter of the protein divided by the total number of coil blocks of the protein

2286. The total number of coil residue blocks in the second quarter of the protein divided by the total number of coil blocks of the protein

2287. The total number of coil residue blocks in the third quarter of the protein divided by the total number of coil blocks of the protein

2288. The total number of coil residue blocks in the fourth quarter of the protein divided by the total number of coil blocks of the protein

2289. The total number of coil residue blocks in the first half of the protein

2290. The total number of coil residue blocks in the three-quarter region of the protein

2291. The total number of coil residue blocks from position 25% to 75% of the protein

2292. The total number of coil residue blocks in the second half of the protein

2293. The total number of coil residue blocks in the first half of the protein divided by the protein length

2294. The total number of coil residue blocks in the three-quarter region of the protein divided by the protein length

2295. The total number of coil residue blocks from position 25% to 75% of the protein divided by the protein length

2296. The total number of coil residue blocks in the second half of the protein divided by the protein length

2297. The total number of coil residue blocks in the first half of the protein divided by the total number of coil blocks of the protein

2298. The total number of coil residue blocks in the three-quarter region of the protein divided by the total number of coil blocks of the protein

2299. The total number of coil residue blocks from position 25% to 75% of the protein divided by the total number of coil blocks of the protein

2300. The total number of coil residue blocks in the second half of the protein divided by the total number of coil blocks of the protein

2301. The length of the maximum coil block in the first quarter of the protein

2302. The median of the coil blocks in the first quarter of the protein

2303. The mean of the coil blocks in the first quarter of the protein

2304. The length of the maximum coil block in the second quarter of the protein

2305. The median of the coil blocks in the second quarter of the protein

2306. The mean of the coil blocks in the second quarter of the protein

2307. The length of the maximum coil block in the third quarter of the protein

2308. The median of the coil blocks in the third quarter of the protein

2309. The mean of the coil blocks in the third quarter of the protein

2310. The length of the maximum coil block in the fourth quarter of the protein

2311. The median of the coil blocks in the fourth quarter of the protein

2312. The mean of the coil blocks in the fourth quarter of the protein

2313. The length of the maximum coil block of the protein

2314. The median of the coil blocks of the protein

2315. The mean of the coil blocks of the protein

2316. The total number of transmembrane helices residues in the sequence

2317. The total number of transmembrane helices residues divided by the length of the protein

2318. The total number of transmembrane helices residues in the first quarter of the sequence

2319. The total number of transmembrane helices residues in the second quarter of the sequence

2320. The total number of transmembrane helices residues in the third quarter of the sequence

2321. The total number of transmembrane helices residues in the fourth quarter of the sequence

2322. The total number of transmembrane helices residues in the first quarter of the sequence divided by the length of the protein

2323. The total number of transmembrane helices residues in the second quarter of the sequence divided by the length of the protein

2324. The total number of transmembrane helices residues in the third quarter of the sequence divided by the length of the protein

2325. The total number of transmembrane helices residues in the fourth quarter of the sequence divided by the length of the protein

2326. The total number of transmembrane helices residues in the first quarter of the sequence divided by the total number of transmembrane helices blocks

2327. The total number of transmembrane helices residues in the first quarter of the sequence divided by the total number of transmembrane helices blocks

2328. The total number of transmembrane helices residues in the first quarter of the sequence divided by the total number of transmembrane helices blocks

2329. The total number of transmembrane helices residues in the first quarter of the sequence divided by the total number of transmembrane helices blocks

2330. The total number of transmembrane helices residues in the first half of the protein

2331. The total number of transmembrane helices residues in the three-quarter region of the protein

2332. The total number of transmembrane helices residues from position 25% to 75% of the protein

2333. The total number of transmembrane helices residues in the second half of the protein

2334. The total number of transmembrane helices residues in the first half of the protein divided by the protein length

2335. The total number of transmembrane helices residues in the three-quarter region of the protein divided by the protein length

2336. The total number of transmembrane helices residues from position 25% to 75% of the protein divided by the protein length

2337. The total number of transmembrane helices residues in the second half of the protein divided by the protein length

2338. The total number of transmembrane helices residues in the first half of the protein divided by the total number of transmembrane helices residues in the protein

2339. The total number of transmembrane helices residues in the three-quarter region of the protein divided by the total number of transmembrane helices residues in the protein

2340. The total number of transmembrane helices residues from position 25% to 75% of the protein divided by the total number of transmembrane helices residues in the protein

2341. The total number of transmembrane helices residues in the second half of the protein divided by the total number of transmembrane helices residues in the protein

2342. The total number of transmembrane helices residue blocks in the protein

2343. The total number of transmembrane helices residue blocks in the first quarter of the protein

2344. The total number of transmembrane helices residue blocks in the second quarter of the protein

2345. The total number of transmembrane helices residue blocks in the third quarter of the protein

2346. The total number of transmembrane helices residue blocks in the fourth quarter of the protein

2347. The total number of transmembrane helices residue blocks in the first quarter of the protein divided by the protein length

2348. The total number of transmembrane helices residue blocks in the second quarter of the protein divided by the protein length

2349. The total number of transmembrane helices residue blocks in the third quarter of the protein divided by the protein length

2350. The total number of transmembrane helices residue blocks in the fourth quarter of the protein divided by the protein length

2351. The total number of transmembrane helices residue blocks in the first quarter of the protein divided by the total number of transmembrane helices blocks of the protein

2352. The total number of transmembrane helices residue blocks in the second quarter of the protein divided by the total number of transmembrane helices blocks of the protein

2353. The total number of transmembrane helices residue blocks in the third quarter of the protein divided by the total number of transmembrane helices blocks of the protein

2354. The total number of transmembrane helices residue blocks in the fourth quarter of the protein divided by the total number of transmembrane helices blocks of the protein

2355. The total number of transmembrane helices residue blocks in the first half of the protein

2356. The total number of transmembrane helices residue blocks in the three-quarter region of the protein

2357. The total number of transmembrane helices residue blocks from position 25% to 75% of the protein

2358. The total number of transmembrane helices residue blocks in the second half of the protein

2359. The total number of transmembrane helices residue blocks in the first half of the protein divided by the protein length

2360. The total number of transmembrane helices residue blocks in the three-quarter region of the protein divided by the protein length

2361. The total number of transmembrane helices residue blocks from position 25% to 75% of the protein divided by the protein length

2362. The total number of transmembrane helices residue blocks in the second half of the protein divided by the protein length

2363. The total number of transmembrane helices residue blocks in the first half of the protein divided by the total number of transmembrane helices blocks of the protein

2364. The total number of transmembrane helices residue blocks in the three-quarter region of the protein divided by the total number of transmembrane helices blocks of the protein

2365. The total number of transmembrane helices residue blocks from position 25% to 75% of the protein divided by the total number of transmembrane helices blocks of the protein

2366. The total number of transmembrane helices residue blocks in the second half of the protein divided by the total number of transmembrane helices blocks of the protein

2367. The length of the maximum transmembrane helices block in the first quarter of the protein

2368. The median of the transmembrane helices blocks in the first quarter of the protein

2369. The mean of the transmembrane helices blocks in the first quarter of the protein

2370. The length of the maximum transmembrane helices block in the second quarter of the protein

2371. The median of the transmembrane helices blocks in the second quarter of the protein

2372. The mean of the transmembrane helices blocks in the second quarter of the protein

2373. The length of the maximum transmembrane helices block in the third quarter of the protein

2374. The median of the transmembrane helices blocks in the third quarter of the protein

2375. The mean of the transmembrane helices blocks in the third quarter of the protein

2376. The length of the maximum transmembrane helices block in the fourth quarter of the protein

2377. The median of the transmembrane helices blocks in the fourth quarter of the protein

2378. The mean of the transmembrane helices blocks in the fourth quarter of the protein

2379. The length of the maximum transmembrane helices block of the protein

2380. The median of the transmembrane helices blocks of the protein

2381. The mean of the transmembrane helices blocks of the protein

2382. The total number of disordered loops or coil residues in the sequence

2383. The total number of disordered loops or coil residues divided by the length of the protein

2384. The total number of disordered loops or coil residues in the first quarter of the sequence

2385. The total number of disordered loops or coil residues in the second quarter of the sequence

2386. The total number of disordered loops or coil residues in the third quarter of the sequence

2387. The total number of disordered loops or coil residues in the fourth quarter of the sequence

2388. The total number of disordered loops or coil residues in the first quarter of the sequence divided by the length of the protein

2389. The total number of disordered loops or coil residues in the second quarter of the sequence divided by the length of the protein

2390. The total number of disordered loops or coil residues in the third quarter of the sequence divided by the length of the protein

2391. The total number of disordered loops or coil residues in the fourth quarter of the sequence divided by the length of the protein

2392. The total number of disordered loops or coil residues in the first quarter of the sequence divided by the total number of disordered loops or coil blocks

2393. The total number of disordered loops or coil residues in the first quarter of the sequence divided by the total number of disordered loops or coil blocks

2394. The total number of disordered loops or coil residues in the first quarter of the sequence divided by the total number of disordered loops or coil blocks

2395. The total number of disordered loops or coil residues in the first quarter of the sequence divided by the total number of disordered loops or coil blocks

2396. The total number of disordered loops or coil residues in the first half of the protein

2397. The total number of disordered loops or coil residues in the three-quarter region of the protein

2398. The total number of disordered loops or coil residues from position 25% to 75% of the protein

2399. The total number of disordered loops or coil residues in the second half of the protein

2400. The total number of disordered loops or coil residues in the first half of the protein divided by the protein length

2401. The total number of disordered loops or coil residues in the three-quarter region of the protein divided by the protein length

2402. The total number of disordered loops or coil residues from position 25% to 75% of the protein divided by the protein length

2403. The total number of disordered loops or coil residues in the second half of the protein divided by the protein length

2404. The total number of disordered loops or coil residues in the first half of the protein divided by the total number of disordered loops or coil residues in the protein

2405. The total number of disordered loops or coil residues in the three-quarter region of the protein divided by the total number of disordered loops or coil residues in the protein

2406. The total number of disordered loops or coil residues from position 25% to 75% of the protein divided by the total number of disordered loops or coil residues in the protein

2407. The total number of disordered loops or coil residues in the second half of the protein divided by the total number of disordered loops or coil residues in the protein

2408. The total number of disordered loops or coil residue blocks in the protein

2409. The total number of disordered loops or coil residue blocks in the first quarter of the protein

2410. The total number of disordered loops or coil residue blocks in the second quarter of the protein

2411. The total number of disordered loops or coil residue blocks in the third quarter of the protein

2412. The total number of disordered loops or coil residue blocks in the fourth quarter of the protein

2413. The total number of disordered loops or coil residue blocks in the first quarter of the protein divided by the protein length

2414. The total number of disordered loops or coil residue blocks in the second quarter of the protein divided by the protein length

2415. The total number of disordered loops or coil residue blocks in the third quarter of the protein divided by the protein length

2416. The total number of disordered loops or coil residue blocks in the fourth quarter of the protein divided by the protein length

2417. The total number of disordered loops or coil residue blocks in the first quarter of the protein divided by the total number of disordered loops or coil blocks of the protein

2418. The total number of disordered loops or coil residue blocks in the second quarter of the protein divided by the total number of disordered loops or coil blocks of the protein

2419. The total number of disordered loops or coil residue blocks in the third quarter of the protein divided by the total number of disordered loops or coil blocks of the protein

2420. The total number of disordered loops or coil residue blocks in the fourth quarter of the protein divided by the total number of disordered loops or coil blocks of the protein

2421. The total number of disordered loops or coil residue blocks in the first half of the protein

2422. The total number of disordered loops or coil residue blocks in the three-quarter region of the protein

2423. The total number of disordered loops or coil residue blocks from position 25% to 75% of the protein

2424. The total number of disordered loops or coil residue blocks in the second half of the protein

2425. The total number of disordered loops or coil residue blocks in the first half of the protein divided by the protein length

2426. The total number of disordered loops or coil residue blocks in the three-quarter region of the protein divided by the protein length

2427. The total number of disordered loops or coil residue blocks from position 25% to 75% of the protein divided by the protein length

2428. The total number of disordered loops or coil residue blocks in the second half of the protein divided by the protein length

2429. The total number of disordered loops or coil residue blocks in the first half of the protein divided by the total number of disordered loops or coil blocks of the protein

2430. The total number of disordered loops or coil residue blocks in the three-quarter region of the protein divided by the total number of disordered loops or coil blocks of the protein

2431. The total number of disordered loops or coil residue blocks from position 25% to 75% of the protein divided by the total number of disordered loops or coil blocks of the protein

2432. The total number of disordered loops or coil residue blocks in the second half of the protein divided by the total number of disordered loops or coil blocks of the protein

2433. The length of the maximum disordered loops or coil block in the first quarter of the protein

2434. The median of the disordered loops or coil blocks in the first quarter of the protein

2435. The mean of the disordered loops or coil blocks in the first quarter of the protein

2436. The length of the maximum disordered loops or coil block in the second quarter of the protein

2437. The median of the disordered loops or coil blocks in the second quarter of the protein

2438. The mean of the disordered loops or coil blocks in the second quarter of the protein

2439. The length of the maximum disordered loops or coil block in the third quarter of the protein

2440. The median of the disordered loops or coil blocks in the third quarter of the protein

2441. The mean of the disordered loops or coil blocks in the third quarter of the protein

2442. The length of the maximum disordered loops or coil block in the fourth quarter of the protein

2443. The median of the disordered loops or coil blocks in the fourth quarter of the protein

2444. The mean of the disordered loops or coil blocks in the fourth quarter of the protein

2445. The length of the maximum disordered loops or coil block of the protein

2446. The median of the disordered loops or coil blocks of the protein

2447. The mean of the disordered loops or coil blocks of the protein

2448. The total number of disordered hot loops residues in the sequence

2449. The total number of disordered hot loops residues divided by the length of the protein

2450. The total number of disordered hot loops residues in the first quarter of the sequence

2451. The total number of disordered hot loops residues in the second quarter of the sequence

2452. The total number of disordered hot loops residues in the third quarter of the sequence

2453. The total number of disordered hot loops residues in the fourth quarter of the sequence

2454. The total number of disordered hot loops residues in the first quarter of the sequence divided by the length of the protein

2455. The total number of disordered hot loops residues in the second quarter of the sequence divided by the length of the protein

2456. The total number of disordered hot loops residues in the third quarter of the sequence divided by the length of the protein

2457. The total number of disordered hot loops residues in the fourth quarter of the sequence divided by the length of the protein

2458. The total number of disordered hot loops residues in the first quarter of the sequence divided by the total number of disordered hot loops blocks

2459. The total number of disordered hot loops residues in the first quarter of the sequence divided by the total number of disordered hot loops blocks

2460. The total number of disordered hot loops residues in the first quarter of the sequence divided by the total number of disordered hot loops blocks

2461. The total number of disordered hot loops residues in the first quarter of the sequence divided by the total number of disordered hot loops blocks

2462. The total number of disordered hot loops residues in the first half of the protein

2463. The total number of disordered hot loops residues in the three-quarter region of the protein

2464. The total number of disordered hot loops residues from position 25% to 75% of the protein

2465. The total number of disordered hot loops residues in the second half of the protein

2466. The total number of disordered hot loops residues in the first half of the protein divided by the protein length

2467. The total number of disordered hot loops residues in the three-quarter region of the protein divided by the protein length

2468. The total number of disordered hot loops residues from position 25% to 75% of the protein divided by the protein length

2469. The total number of disordered hot loops residues in the second half of the protein divided by the protein length

2470. The total number of disordered hot loops residues in the first half of the protein divided by the total number of disordered hot loops residues in the protein

2471. The total number of disordered hot loops residues in the three-quarter region of the protein divided by the total number of disordered hot loops residues in the protein

2472. The total number of disordered hot loops residues from position 25% to 75% of the protein divided by the total number of disordered hot loops residues in the protein

2473. The total number of disordered hot loops residues in the second half of the protein divided by the total number of disordered hot loops residues in the protein

2474. The total number of disordered hot loops residue blocks in the protein

2475. The total number of disordered hot loops residue blocks in the first quarter of the protein

2476. The total number of disordered hot loops residue blocks in the second quarter of the protein

2477. The total number of disordered hot loops residue blocks in the third quarter of the protein

2478. The total number of disordered hot loops residue blocks in the fourth quarter of the protein

2479. The total number of disordered hot loops residue blocks in the first quarter of the protein divided by the protein length

2480. The total number of disordered hot loops residue blocks in the second quarter of the protein divided by the protein length

2481. The total number of disordered hot loops residue blocks in the third quarter of the protein divided by the protein length

2482. The total number of disordered hot loops residue blocks in the fourth quarter of the protein divided by the protein length

2483. The total number of disordered hot loops residue blocks in the first quarter of the protein divided by the total number of disordered hot loops blocks of the protein

2484. The total number of disordered hot loops residue blocks in the second quarter of the protein divided by the total number of disordered hot loops blocks of the protein

2485. The total number of disordered hot loops residue blocks in the third quarter of the protein divided by the total number of disordered hot loops blocks of the protein

2486. The total number of disordered hot loops residue blocks in the fourth quarter of the protein divided by the total number of disordered hot loops blocks of the protein

2487. The total number of disordered hot loops residue blocks in the first half of the protein

2488. The total number of disordered hot loops residue blocks in the three-quarter region of the protein

2489. The total number of disordered hot loops residue blocks from position 25% to 75% of the protein

2490. The total number of disordered hot loops residue blocks in the second half of the protein

2491. The total number of disordered hot loops residue blocks in the first half of the protein divided by the protein length

2492. The total number of disordered hot loops residue blocks in the three-quarter region of the protein divided by the protein length

2493. The total number of disordered hot loops residue blocks from position 25% to 75% of the protein divided by the protein length

2494. The total number of disordered hot loops residue blocks in the second half of the protein divided by the protein length

2495. The total number of disordered hot loops residue blocks in the first half of the protein divided by the total number of disordered hot loops blocks of the protein

2496. The total number of disordered hot loops residue blocks in the three-quarter region of the protein divided by the total number of disordered hot loops blocks of the protein

2497. The total number of disordered hot loops residue blocks from position 25% to 75% of the protein divided by the total number of disordered hot loops blocks of the protein

2498. The total number of disordered hot loops residue blocks in the second half of the protein divided by the total number of disordered hot loops blocks of the protein

2499. The length of the maximum disordered hot loops block in the first quarter of the protein

2500. The median of the disordered hot loops blocks in the first quarter of the protein

2501. The mean of the disordered hot loops blocks in the first quarter of the protein

2502. The length of the maximum disordered hot loops block in the second quarter of the protein

2503. The median of the disordered hot loops blocks in the second quarter of the protein

2504. The mean of the disordered hot loops blocks in the second quarter of the protein

2505. The length of the maximum disordered hot loops block in the third quarter of the protein

2506. The median of the disordered hot loops blocks in the third quarter of the protein

2507. The mean of the disordered hot loops blocks in the third quarter of the protein

2508. The length of the maximum disordered hot loops block in the fourth quarter of the protein

2509. The median of the disordered hot loops blocks in the fourth quarter of the protein

2510. The mean of the disordered hot loops blocks in the fourth quarter of the protein

2511. The length of the maximum disordered hot loops block of the protein

2512. The median of the disordered hot loops blocks of the protein

2513. The mean of the disordered hot loops blocks of the protein

2514. The total number of disordered (missing coordinates in X-Ray structure) residues in the sequence

2515. The total number of disordered (missing coordinates in X-Ray structure) residues divided by the length of the protein

2516. The total number of disordered (missing coordinates in X-Ray structure) residues in the first quarter of the sequence

2517. The total number of disordered (missing coordinates in X-Ray structure) residues in the second quarter of the sequence

2518. The total number of disordered (missing coordinates in X-Ray structure) residues in the third quarter of the sequence

2519. The total number of disordered (missing coordinates in X-Ray structure) residues in the fourth quarter of the sequence

2520. The total number of disordered (missing coordinates in X-Ray structure) residues in the first quarter of the sequence divided by the length of the protein

2521. The total number of disordered (missing coordinates in X-Ray structure) residues in the second quarter of the sequence divided by the length of the protein

2522. The total number of disordered (missing coordinates in X-Ray structure) residues in the third quarter of the sequence divided by the length of the protein

2523. The total number of disordered (missing coordinates in X-Ray structure) residues in the fourth quarter of the sequence divided by the length of the protein

2524. The total number of disordered (missing coordinates in X-Ray structure) residues in the first quarter of the sequence divided by the total number of disordered (missing coordinates in X-Ray structure) blocks

2525. The total number of disordered (missing coordinates in X-Ray structure) residues in the first quarter of the sequence divided by the total number of disordered (missing coordinates in X-Ray structure) blocks

2526. The total number of disordered (missing coordinates in X-Ray structure) residues in the first quarter of the sequence divided by the total number of disordered (missing coordinates in X-Ray structure) blocks

2527. The total number of disordered (missing coordinates in X-Ray structure) residues in the first quarter of the sequence divided by the total number of disordered (missing coordinates in X-Ray structure) blocks

2528. The total number of disordered (missing coordinates in X-Ray structure) residues in the first half of the protein

2529. The total number of disordered (missing coordinates in X-Ray structure) residues in the three-quarter region of the protein

2530. The total number of disordered (missing coordinates in X-Ray structure) residues from position 25% to 75% of the protein

2531. The total number of disordered (missing coordinates in X-Ray structure) residues in the second half of the protein

2532. The total number of disordered (missing coordinates in X-Ray structure) residues in the first half of the protein divided by the protein length

2533. The total number of disordered (missing coordinates in X-Ray structure) residues in the three-quarter region of the protein divided by the protein length

2534. The total number of disordered (missing coordinates in X-Ray structure) residues from position 25% to 75% of the protein divided by the protein length

2535. The total number of disordered (missing coordinates in X-Ray structure) residues in the second half of the protein divided by the protein length

2536. The total number of disordered (missing coordinates in X-Ray structure) residues in the first half of the protein divided by the total number of disordered (missing coordinates in X-Ray structure) residues in the protein

2537. The total number of disordered (missing coordinates in X-Ray structure) residues in the three-quarter region of the protein divided by the total number of disordered (missing coordinates in X-Ray structure) residues in the protein

2538. The total number of disordered (missing coordinates in X-Ray structure) residues from position 25% to 75% of the protein divided by the total number of disordered (missing coordinates in X-Ray structure) residues in the protein

2539. The total number of disordered (missing coordinates in X-Ray structure) residues in the second half of the protein divided by the total number of disordered (missing coordinates in X-Ray structure) residues in the protein

2540. The total number of disordered (missing coordinates in X-Ray structure) residue blocks in the protein

2541. The total number of disordered (missing coordinates in X-Ray structure) residue blocks in the first quarter of the protein

2542. The total number of disordered (missing coordinates in X-Ray structure) residue blocks in the second quarter of the protein

2543. The total number of disordered (missing coordinates in X-Ray structure) residue blocks in the third quarter of the protein

2544. The total number of disordered (missing coordinates in X-Ray structure) residue blocks in the fourth quarter of the protein

2545. The total number of disordered (missing coordinates in X-Ray structure) residue blocks in the first quarter of the protein divided by the protein length

2546. The total number of disordered (missing coordinates in X-Ray structure) residue blocks in the second quarter of the protein divided by the protein length

2547. The total number of disordered (missing coordinates in X-Ray structure) residue blocks in the third quarter of the protein divided by the protein length

2548. The total number of disordered (missing coordinates in X-Ray structure) residue blocks in the fourth quarter of the protein divided by the protein length

2549. The total number of disordered (missing coordinates in X-Ray structure) residue blocks in the first quarter of the protein divided by the total number of disordered (missing coordinates in X-Ray structure) blocks of the protein

2550. The total number of disordered (missing coordinates in X-Ray structure) residue blocks in the second quarter of the protein divided by the total number of disordered (missing coordinates in X-Ray structure) blocks of the protein

2551. The total number of disordered (missing coordinates in X-Ray structure) residue blocks in the third quarter of the protein divided by the total number of disordered (missing coordinates in X-Ray structure) blocks of the protein

2552. The total number of disordered (missing coordinates in X-Ray structure) residue blocks in the fourth quarter of the protein divided by the total number of disordered (missing coordinates in X-Ray structure) blocks of the protein

2553. The total number of disordered (missing coordinates in X-Ray structure) residue blocks in the first half of the protein

2554. The total number of disordered (missing coordinates in X-Ray structure) residue blocks in the three-quarter region of the protein

2555. The total number of disordered (missing coordinates in X-Ray structure) residue blocks from position 25% to 75% of the protein

2556. The total number of disordered (missing coordinates in X-Ray structure) residue blocks in the second half of the protein

2557. The total number of disordered (missing coordinates in X-Ray structure) residue blocks in the first half of the protein divided by the protein length

2558. The total number of disordered (missing coordinates in X-Ray structure) residue blocks in the three-quarter region of the protein divided by the protein length

2559. The total number of disordered (missing coordinates in X-Ray structure) residue blocks from position 25% to 75% of the protein divided by the protein length

2560. The total number of disordered (missing coordinates in X-Ray structure) residue blocks in the second half of the protein divided by the protein length

2561. The total number of disordered (missing coordinates in X-Ray structure) residue blocks in the first half of the protein divided by the total number of disordered (missing coordinates in X-Ray structure) blocks of the protein

2562. The total number of disordered (missing coordinates in X-Ray structure) residue blocks in the three-quarter region of the protein divided by the total number of disordered (missing coordinates in X-Ray structure) blocks of the protein

2563. The total number of disordered (missing coordinates in X-Ray structure) residue blocks from position 25% to 75% of the protein divided by the total number of disordered (missing coordinates in X-Ray structure) blocks of the protein

2564. The total number of disordered (missing coordinates in X-Ray structure) residue blocks in the second half of the protein divided by the total number of disordered (missing coordinates in X-Ray structure) blocks of the protein

2565. The length of the maximum disordered (missing coordinates in X-Ray structure) block in the first quarter of the protein

2566. The median of the disordered (missing coordinates in X-Ray structure) blocks in the first quarter of the protein

2567. The mean of the disordered (missing coordinates in X-Ray structure) blocks in the first quarter of the protein

2568. The length of the maximum disordered (missing coordinates in X-Ray structure) block in the second quarter of the protein

2569. The median of the disordered (missing coordinates in X-Ray structure) blocks in the second quarter of the protein

2570. The mean of the disordered (missing coordinates in X-Ray structure) blocks in the second quarter of the protein

2571. The length of the maximum disordered (missing coordinates in X-Ray structure) block in the third quarter of the protein

2572. The median of the disordered (missing coordinates in X-Ray structure) blocks in the third quarter of the protein

2573. The mean of the disordered (missing coordinates in X-Ray structure) blocks in the third quarter of the protein

2574. The length of the maximum disordered (missing coordinates in X-Ray structure) block in the fourth quarter of the protein

2575. The median of the disordered (missing coordinates in X-Ray structure) blocks in the fourth quarter of the protein

2576. The mean of the disordered (missing coordinates in X-Ray structure) blocks in the fourth quarter of the protein

2577. The length of the maximum disordered (missing coordinates in X-Ray structure) block of the protein

2578. The median of the disordered (missing coordinates in X-Ray structure) blocks of the protein

2579. The mean of the disordered (missing coordinates in X-Ray structure) blocks of the protein
